# Tree diversity and soil chemical properties drive the linkages between soil microbial community and ecosystem functioning

**DOI:** 10.1038/s43705-021-00040-0

**Published:** 2021-08-23

**Authors:** Rémy Beugnon, Jianqing Du, Simone Cesarz, Stephanie D. Jurburg, Zhe Pang, Bala Singavarapu, Tesfaye Wubet, Kai Xue, Yanfen Wang, Nico Eisenhauer

**Affiliations:** 1 German Centre for Integrative Biodiversity Research (iDiv) Halle-Jena-Leipzig, Puschstrasse 4, Leipzig, Germany; 2 Institute of Biology, Leipzig University, Puschstrasse 4, Leipzig, Germany; 3 Yanshan Earth Critical Zone and Surface Fluxes Research Station, College of Resources and Environment, University of Chinese Academy of Sciences, Beijing, China; 4 UFZ-Helmholtz Centre for Environmental Research, Department of Community Ecology, Theodor-Lieser-Str. 4, Halle (Saale), Germany; 5 Institute of Biology/Geobotany and Botanical Garden, Martin Luther University Halle-Wittenberg, Am Kirchtor, Halle, Germany; 6 CAS Center for Excellence in Tibetan Plateau Earth Sciences, Beijing, China

**Keywords:** Microbial ecology, Biodiversity

## Abstract

Microbial respiration is critical for soil carbon balance and ecosystem functioning. Previous studies suggest that plant diversity influences soil microbial communities and their respiration. Yet, the linkages between tree diversity, microbial biomass, microbial diversity, and microbial functioning have rarely been explored. In this study, we measured two microbial functions (microbial physiological potential, and microbial respiration), together with microbial biomass, microbial taxonomic and functional profiles, and soil chemical properties in a tree diversity experiment in South China, to disentangle how tree diversity affects microbial respiration through the modifications of the microbial community. Our analyses show a significant positive effect of tree diversity on microbial biomass (+25% from monocultures to 24-species plots), bacterial diversity (+12%), and physiological potential (+12%). In addition, microbial biomass and physiological potential, but not microbial diversity, were identified as the key drivers of microbial respiration. Although soil chemical properties strongly modulated soil microbial community, tree diversity increased soil microbial respiration by increasing microbial biomass rather than changing microbial taxonomic or functional diversity. Overall, our findings suggest a prevalence of microbial biomass over diversity in controlling soil carbon dynamics.

## Introduction

A thorough understanding of the soil carbon balance is essential to mitigate recent increases in atmospheric carbon concentrations and the resulting climate change [1–4]. Soil heterotrophic respiration is a critical process for the soil carbon balance and ecosystem functions such as climate regulation, nutrient cycling, and plant productivity [5, 6]. Microorganisms are the main contributors to soil heterotrophic respiration, and microbial respiration is tightly linked to microbial community properties [7–11]. In turn, soil microbes and their functioning are determined by the biotic and abiotic environmental conditions [12–14].

Microbial properties are strongly affected by the vegetation type [15] and its diversity [16, 17]. Consequently, plant community composition and diversity mediate microbial control over the soil carbon balance [16–20]. Plant diversity can increase litter and rhizosphere carbon inputs into the soil, thereby enhancing the quality and quantity of resources for the soil microbial community [21, 22]. This increase of rhizosphere carbon was shown to enhance soil carbon storage [19, 23] by increasing soil microbial biomass and activity [19, 24]. However, how plant diversity modulates the microbial community and how this affects soil carbon dynamics is not well understood. In addition, abiotic conditions, such as climate and soil chemical properties (soil carbon, nitrogen and phosphorus concentrations, pH, and humidity) also drive the assembly and functioning of soil microbial communities [12, 13, 25, 26]. For example, soil organic carbon content is generally correlated with microbial biomass and activity [19, 27], while nitrogen and phosphorus-limited soils exhibit reduced microbial biomass and microbial community diversity [28, 29]. Importantly, the effect of abiotic conditions on soil microbes greatly depends on which facet of the microbiota is assessed [30–32].

**Fig. 1:**
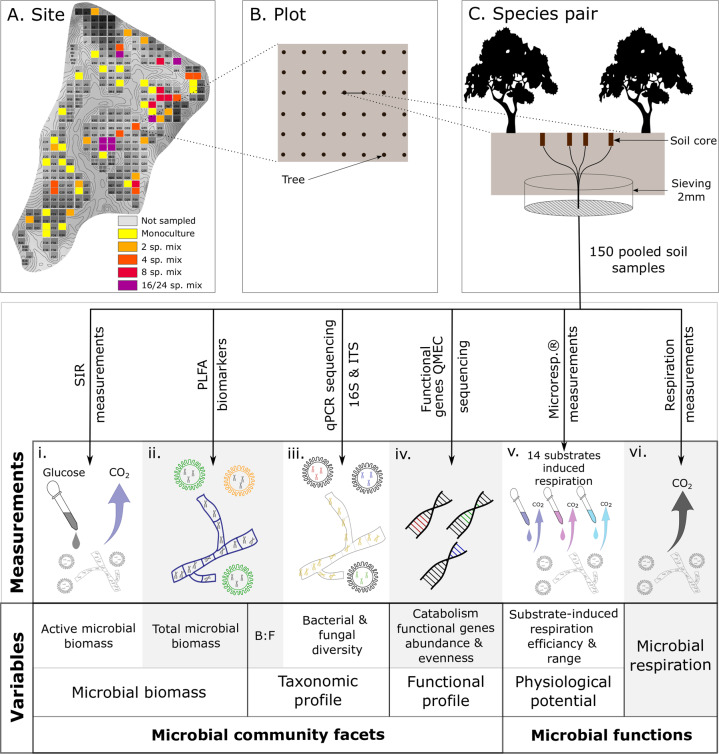
Sampling and measurement design. Sampling design: **A** Plot layout of the BEF China experimental platform (site A), **B** Plot tree planting grid pattern, **C** Soil core sampling design in tree species pairs, and treatment of samples. Measurements**:** (i) quantification of active microbial biomass by substrate-induced respiration method (i.e., SIR, Scheu et al. [55]), (ii) quantification of total microbial biomass and bacterial to fungal biomass ratio (B:F ratio) by measurement of soil microbial phospholipid fatty acids (PLFAs), (iii) qualification of microbial profile by qPCR sequencing of soil 16S and ITS sequences, (iv) quantification of functional genes related to carbon catabolism by quantitative microbial element cycling (QMEC, Zheng et al. [64]), (v) quantification of carbon dioxide released during 6 h after induction by a range of substrates using MicroResp.^®^ method (Campbell et al. [65]), (vi) quantification of soil microbial respiration by the O_2_-microcompensation method.

Soil microbial abundance, taxonomic and functional diversity can be assessed in terms of microbial biomass (i.e., through phospholipid fatty acid (PLFA) biomarkers or substrate-induced respiration measurements), taxonomic community composition and diversity (i.e., taxonomic profile through 16S rRNA gene and ITS amplicon sequencing or PLFA biomarker measurements), or potential functioning (i.e., functional profile through shotgun metagenomics or qPCR of functional genes), respectively (Fig. 1). Realized functions can be assessed by community level physiological profiling (i.e., physiological potential through MicroResp ^®^ measurements) or microbial respiration measurements (Fig. 1). For example, the taxonomic diversity of soil microbes generally correlates with functional diversity [33], but these relationships may decouple as results of microbial functional redundancy and the different sensitivities of microbial facets to environmental changes [30, 34, 35]. Alternatively, combining several measurements of the soil microbial community may provide a deeper understanding of soil microbial functioning; however, the different facets of soil microbial communities are rarely assessed together.

Taken together, soil microbial biomass, taxonomic and functional profiles are three key facets of the microbial community shown to be critical for microbial respiration [8, 36, 37], but they have not been studied together. Consequently, little is known about the potential correlations between these microbial facets, and their relationship to microbial functions [8, 36–38]. For example, microbial respiration is tightly linked to the total microbial biomass and the microbial taxonomic profile [7–11], but the microbial functional profile has been shown to be more relevant than the taxonomic profile to predict microbial realized functions [36, 38, 39]. Moreover, microbial respiration is strongly limited by the microbial physiological ability to process the available substrates [40, 41]. Therefore, the microbial physiological potential to process substrate is expected to be a powerful predictor of microbial respiration and functions [40, 42]. The physiological potential is believed to be dependent on the microbial biomass, as well as the taxonomic and functional profiles [42–45]. By predicting enzymatic activity [37, 39], the microbial functional profile is hypothesized to be more closely related to the physiological potential of the soil microbial community than microbial biomass or taxonomic profile. However, no study has tested the individual or combined ability of these different microbial facets to predict the microbial physiological potential. A better understanding of the relationship between microbial facets and realized microbial functions may facilitate the integration of soil microbial processes into soil carbon flux models [46–49].

To mechanistically understand tree diversity and soil chemical properties effects on microbial functions, we sampled a subtropical forest experiment in China [50], and explored the contribution of different facets of the microbial community to microbial functions by bringing these microbial facets and functions together in a common framework. This biome has the highest average net ecosystem productivity among Asian forests [51] and is thus ideal for the study of carbon cycling and its determinants. In 2018, we collected 150 samples in 52 plots from a tree diversity experiment established in 2009. Across a tree species richness gradient, we measured soil microbial respiration, biomass, taxonomic and functional profiles, and physiological potential, along with soil chemical properties (carbon, nitrogen, and phosphorus concentrations, soil humidity, and pH). We hypothesized that (H1) tree diversity would drive microbial community facets (microbial biomass, taxonomic and functional profile) and increase soil microbial functioning (microbial physiological potential and respiration); (H2) soil microbial biomass, taxonomic and functional profiles would be tightly correlated with each other and together drive microbial functions; (H3) microbial physiological potential would link microbial biomass, taxonomic and functional profiles to microbial respiration; and (H4) that environmental conditions (tree diversity and soil chemical properties) would co-determine soil respiration by modulating the microbial community facets.

## Materials and methods

Only key procedures are provided here, further details about the materials and methods are available in Supplementary S1.

### Study site, study design, and sampling

Our study site was located in south-east China in the Jiangxi province (29.08-29.11° N, 117.90–117.93° E). Sampling took place in BEF-China, a tree diversity experiment, including tree species mixture plots (1, 2, 4, 8, and 16 tree species per plot, Fig. 1) [50]. To account for the role of tree diversity and soil quality, we collected 150 soil samples across different levels of tree diversity randomly distributed in the landscape (Fig. 1, Supplementary S2). We sampled from mid-August to late-September 2018, before the litterfall season. To avoid spatio-temporal autocorrelation, the daily sample location was chosen randomly; and to control for the distance to the trees, each sample was extracted between a pair of trees. For each pair of trees, we extracted four soil cores (5 cm diameter; 10 cm depth), 5 and 20 cm away from the center point between the tree pair (Fig. 1). A composite sample was built from these four cores by homogenizing with a 2 mm sieve.

### Soil quality analyses

Soil moisture was measured from 25 g of soil by drying at 40 °C for two days. A subsample was used to measure soil pH in a 1:2.5 soil-water solution. In addition, to measure soil total organic carbon (TOC), total nitrogen (TN), and total phosphorus (TP), 200 g of soil were homogenized, ground with a ball mill, and sieved at 0.25 mm. Soil TOC was measured by a TOC Analyzer (Liqui TOC II; Elementar Analysensysteme GmbH, Hanau, Germany). Soil TN was measured on an auto-analyzer (SEAL Analytical GmbH, Norderstedt, Germany) using the Kjeldahl method [52]. Soil TP concentration was measured after wet digestion with H_2_SO_4_ and HClO_4_ by a UV–VIS spectrophotometer (UV2700, SHIMADZU, Japan). Carbon to nitrogen and carbon to phosphorus ratios were calculated as TOC:TN and TOC:TP, respectively.

### Soil microbial biomass

Microbial biomass was measured using PLFA analysis. PLFAs were extracted from 5 g of frozen soil following Frostegård et al. [53]. Biomarkers were assigned to microbial functional groups according to Ruess et al. [54], see Supplementary S3. Total microbial biomass was calculated as the sum of biomasses of all microbial groups. The ratio of bacteria to fungi (B:F) was calculated as the ratio of the sum of all bacterial biomass markers to the sum of all fungal biomass markers. Active microbial biomass was measured from 6 g of soil using the substrate-induced respiration method following Scheu et al. [55].

### Soil microbial taxonomic profile

Microbial DNA was extracted from freeze-dried soil samples using PowerSoil DNA Isolation Kit (MO BIO Laboratories Inc., Carlsbad, CA, United States). DNA concentrations were checked with a NanoDrop spectrophotometer (Thermo Fisher Scientific, Dreieich, Germany), and the extracts were adjusted to 10–15 ng/µl. The bacterial and fungal amplicon libraries were prepared following Schöps et al. [56] and Nawaz et al. [57].

Bioinformatic analysis was performed using the Quantitative Insights into Microbial Ecology—QIIME 2 2020.2 [58]. The forward and reverse reads were demultiplexed, primer sequences were trimmed, denoised, and grouped into Amplicon Sequence Variants (ASVs) using cut-adapt for chimera removal [59], via q2-cutadapt and DADA2 for non-target taxa removal [60], via q2-dada2. ASV tables were imported into R with the phyloseq package [61]. The fungal and bacterial ASVs were rarefied to 16,542 and 28,897 reads per sample, respectively. OTU richness, Shannon diversity, Pielou evenness, and Gini dominance indices were calculated using the microbiome package [62]. We inspected the correlations between these indices and focused our analyses on Shannon diversity index (Supplementary S4A).

### Soil microbial functional profile

DNA was extracted with the FastDNA Spin Kit for Soil (MP Biomedicals, USA) following the manufacturer’s instructions. DNA concentrations were checked with a NanoDrop spectrophotometer (Thermo Fisher Scientific, Dreieich, USA), and DNA concentrations were quantified with the QuantiFluor dsDNA kit (Promega, USA) and a microplate reader (SpectraMax M5, Molecular Devices). DNA was diluted to 50 ng/µl with sterile water and stored at −20 °C. Microbial functional genes coding for enzymes involved in carbon catabolism processes, which are central to soil carbon cycling [63], see Supplementary S5, were quantified following Zheng et al. [64] using a high-throughput quantitative-PCR-based chip (SmartChip Real-time PCR system, WaferGen Biosystems, Fremont, USA).

To compare abundance patterns across functional genes, we scaled each functional gene abundance between 0 and 1 across all samples using the z-transformation, and we summed the scaled abundance of functional genes related to carbon catabolism (i.e., “Cata”, Supplementary S5). To quantify the evenness of the functional gene abundances, the functional gene Pielou evenness was calculated using the R ‘diversity’ from the ‘vegan’ package (“FG evenness”).

### Soil microbial physiological potential

Microbial physiological potential indices were calculated from substrate-induced respiration assays using the MicroResp.^®^ method [65]. This method is used to assess the potential response of the living microbial community (i.e., active and dormant) to substrate addition. Fourteen substrates from three chemical classes (i.e., saccharides, amino-acid, and carboxylic acids) were selected to cover complementary biochemical pathways and to create a gradient of molecular weights (ranging from 89 to 221 g mol^−1^), and a gradient of carbon oxidation states (ranging from −2 to 3 e^−^, Supplementary S6). CO_2_ measurements were used to calculate substrate-induced respiration efficiency (i.e., “SIR efficiency”) and SIR response range (i.e., “SIR range”). SIR efficiency was calculated as the Pielou evenness (from R ‘diversity’ function package vegan) of the CO_2_ production of all substrates. SIR range was defined as the difference in CO_2_ production between oxalic acid and alanine, the two substrates on the upper and lower extremes of carbon oxidation. We performed sensitivity analyses to explore the effects of substrate selection on these indices, which showed that substrate selection did not alter our results and conclusions (Supplementary S6).

### Soil microbial respiration

Soil microbial respiration was measured on 6 g of fresh soil following Scheu et al. [55] without adding any substrate or water, thereby reflecting the actual respiration at the site. Active microbial biomass (with substrate addition) and microbial respiration (without substrate addition) were measured on the same sample and machine. To test the robustness of our results, all following analyses were run with and without active microbial biomass.

### Statistical analyses

All data handling and statistical analyses were performed using the R statistical software version 4.0.3, and all R scripts used for this study can be found in our GitHub repository (https://github.com/remybeugnon/Beugnon-Du_et_al_2021_Microbial_community_and_functions). All metrics inferred from soil measurements are summarized in the Supplementary S4. In order to avoid any model-fit deviation due to scale differences between variables, all explanatory variables were centered and divided by two standard deviations for our analyses using the R rescale function from the arm package. For each analysis, we compared the drivers’ effect sizes defined as the standardized estimate of a given variable in the model, where the response variable was centered and divided by two standard deviations.

#### Tree diversity effects on soil microbial community facets and functions

We used linear models and normal distribution assumptions to test the effects of tree species richness on soil microbial biomass (total and active microbial biomass), taxonomic profile (B:F ratio and Shannon diversity of bacterial and fungal communities), functional profile (catabolic functional gene abundance and evenness), physiological potential (SIR efficiency and range), and microbial respiration. Possible non-linear relations (i.e., quadratic, polynomial, and logarithmic relationships) were tested and are shown in Supplementary S7A. The linear relationships were chosen when the difference in AIC with the best model (i.e., model with the lowest AIC) was lower than four. All previous linear models were tested in R using the lm function, and statistical hypotheses of the following linear models were tested in Supplementary S7B using the model_check function from the performance package in R.

#### Relationships between soil microbial facets and microbial functions

We tested the correlations between the microbial community facets (soil microbial biomass, taxonomic and functional profiles) using Pearson correlation tests. We used linear multivariate models and normal distribution assumptions to test the effects of microbial biomass (total and active microbial biomass), taxonomic profile (B:F ratio and Shannon diversity of bacterial and fungal communities), and functional profile (catabolic functional gene abundance, and evenness) on soil microbial physiological potential (SIR efficiency and range), and soil microbial respiration. Explanatory variables (microbial biomasses, taxonomic and functional profile indices) were selected using forward and backward step selection based on AIC (i.e., R step function from stats package). A variance partitioning analysis was performed on the final set of variables to disentangle the effects of microbial biomass, taxonomic and functional profiles using the R varpart function from the vegan package. All previous linear multivariate models were tested in R using the lm function and statistical hypotheses of the following linear models were tested in Supplementary S8 using the model_check function from the performance package in R.

#### Cascading effects of the different soil microbial community facets on microbial physiological potential and microbial respiration

We tested the relationships between soil microbial biomass, taxonomic and functional profiles, physiological potential, and soil microbial respiration using a Structural Equation Modeling (SEM) framework. Microbial biomass, taxonomic and functional profiles were linked to each other by correlations, and their effects on physiological potential indices and soil microbial respiration were modeled with causal relations (directed paths). Our SEM was fitted using the R sem function from the lavaan package [66]. The model fit to our data and model quality were estimated using three complementary indices: (i) the root mean square error of approximation (RMSEA), (ii) the comparative fit index (CFI), and (iii) the standardized root mean squared residuals (SRMR). Model fits were considered acceptable when RMSEA < 0.10, CFI > 0.9 and SRMR < 0.08. All statistical hypotheses and complete outputs can be found in Supplementary S9 and S10.

#### Effects of tree species richness and soil quality on relationships between the soil microbial community and their functions

To test the effects of tree species richness and soil chemical properties on the relationship between the soil microbial community facets and microbial respiration, we added the causal effects of soil chemical properties and tree species richness on the variables of our previous SEM model. To assess which group of response variables (i.e., soil microbial biomass, taxonomic profile, functional profile, physiological potential, and microbial respiration) was the most affected by soil chemical properties and tree species richness, the effects of soil chemical properties and tree species richness on each response group were summarized by summing all the absolute standardized effects of soil quality or tree species richness on the given response group. Additionally, to assess the importance of each soil chemical property and tree species richness, we summed the absolute standardized effects of each soil chemical property and tree species richness. All statistical hypotheses and complete outputs can be found in Supplementary S9 and S11.

## Results

### Tree diversity enhances the soil microbial biomass, diversity and functions

**Fig. 2:**
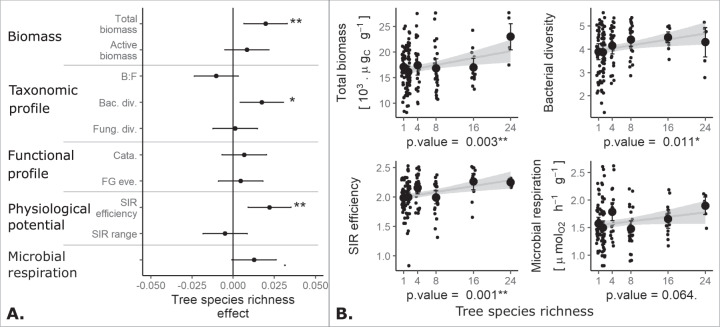
Tree species richness effects on soil microbial community facets and functions. **A** Effect of tree species richness on microbial biomass (i.e., “Total biomass” and “Active biomass”), taxonomic profile (i.e., bacteria to fungi ratio: “B:F”, bacteria Shannon diversity: “Bac. div.”, and fungi Shannon diversity: “Fung. div.”), functional profile (i.e., the abundance of catabolism functional genes: “Cata” and functional genes evenness: “FG eve.”), physiological potential (i.e., substrate-induced respiration efficiency: “SIR efficiency”, and substrate-induced respiration response range: “SIR range”), and microbial respiration. **B** Relations between tree species richness and total microbial biomass, bacteria Shannon diversity (i.e., “Bacteria diversity”), SIR efficiency, and microbial respiration. The significance levels were standardized across the panels (“.”*: p*-value < 0.1, “*”: *p*-value < 0.05, “**”: *p*-value < 0.01, and “***”: *p*-value < 0.001).

Our analyses showed that tree species richness enhanced soil microbial community properties and functions. Total microbial biomass and bacterial diversity increased significantly with tree species richness (total microbial biomass: estimate ± SE = 0.020 ± 0.007, *p*-value = 0.003; bacteria diversity: 0.017 ± 0.007, *p*-value = 0.011; Fig. 2). Tree species richness significantly increased soil microbial community substrate-induced respiration efficiency (SIR efficiency: 0.022 ± 0.007, *p*-value = 0.001) and tended to increase microbial respiration (0.013 ± 0.007, *p*-value = 0.064, Fig. 2). Notably, the tree diversity effect on total biomass and basal respiration were mostly driven by high values in 24-species tree communities for microbial biomass and lower variability for respiration (Fig. 2, Supplementary S7A).

### Soil microbial community facets are strongly correlated

**Fig. 3:**
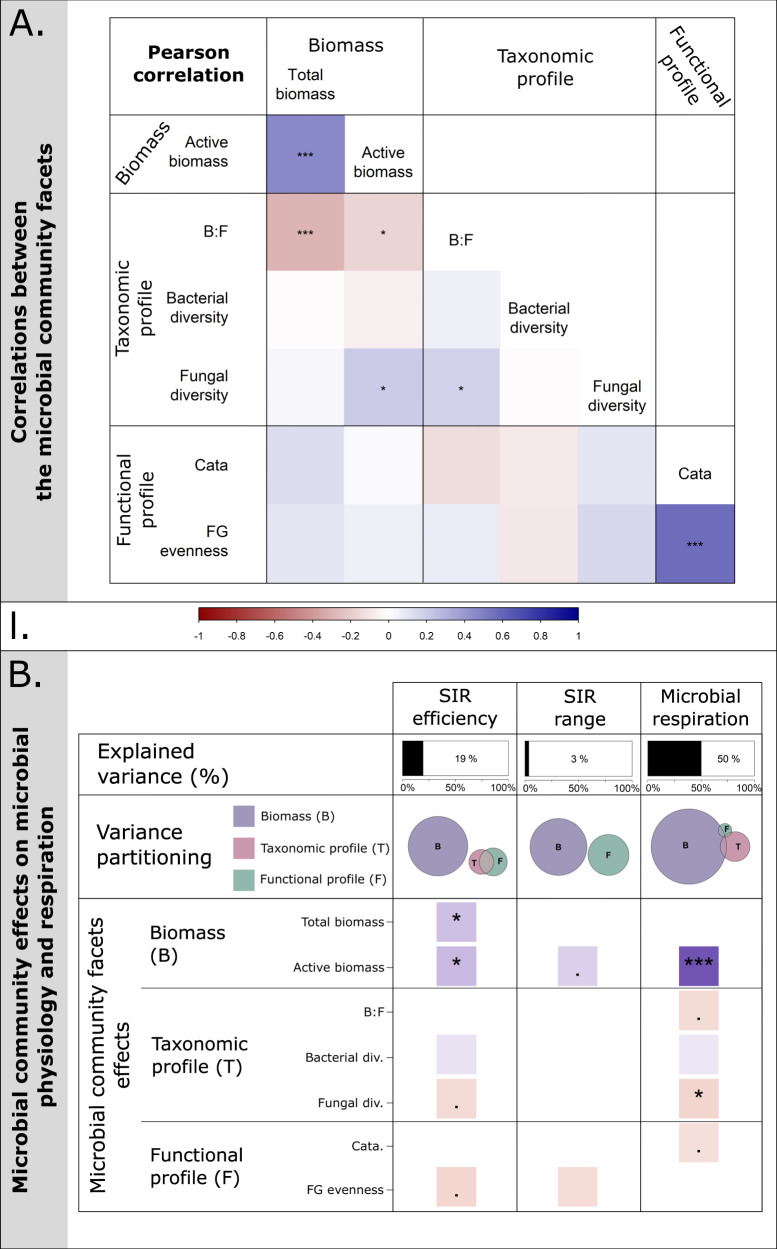
Correlation between soil microbial community facets and their effects on soil microbial function. Correlations between soil microbial community facets (**A**), and effect of soil microbial community facets on microbial functions (**B**). **A** Correlation matrix of soil microbial community facets: microbial biomass (i.e., “total biomass” and “active biomass”), taxonomic profile (i.e., bacteria to fungi ratio: “B:F”, bacteria Shannon diversity, and fungi Shannon diversity), functional profile (i.e., the abundance of catabolism functional genes: “Cata” and functional genes evenness: “FG evenness”). **B** Effects of microbial community facets on substrate-induced respiration efficiency and response range (i.e., “SIR efficiency” and “SIR range”, respectively), and microbial respiration. The explained variance (in %) of the model after model selection is displayed in the first row. The model variance partitioning between the different microbial facets (i.e., biomass, taxonomic and functional profile) is displayed in the second row. For each response variable (i.e., column), the circles are proportional to the part of explained variance and the intersects to the shared variance between two groups of variables. The last rows display the standardized effect sizes of the selected variables. The significance levels were standardized across the panels (*“.”: p*-value < 0.1., “*”: *p*-value < 0.05, “**”: *p*-value <0.01, and “***”: *p*-value < 0.001). l. Color scale. The colored bar represents both the correlation strength in **A** and the effect size of the microbial community facets in **B** both between −1 and 1.

We observed a positive correlation between total soil microbial biomass and active microbial biomass (Pearson correlation: cor = 0.45, *p*-value < 0.001), as well as a positive correlation between the functional profile variables (cor = 0.57, *p*-value < 0.001). In addition, the bacteria to fungi ratio (B:F) was negatively correlated to microbial biomass and the Shannon diversity of fungi (see Fig. 3A, and Supplementary S8), while the Shannon diversity of fungi was positively correlated to active microbial biomass (cor = 0.20, *p*-value = 0.014; Fig. 3A, Supplementary S8).

### Soil microbial community facets drive soil microbial functions

We tested the effects of soil microbial biomass and taxonomic and functional profile on microbial community physiological potential and respiration using linear models and AIC-based model selection. Soil microbial community facets explained up to 50% of the variance in microbial respiration, but only 19% and 4% of the variance in SIR efficiency and range, respectively (Fig. 3B). For all microbial functions, microbial biomass was the main driver by explaining up to 43% of microbial respiration, 14% of SIR efficiency, and 2% of substrate-induced respiration response range (Fig. 3B, Supplementary S8). Together, microbial taxonomic and functional profile only explained a small part of the variance in microbial respiration (taxonomic profile: 6% and functional profile: <1%, Supplementary S8), substrate-induced respiration efficiency (taxonomic profile: 1% and functional profile: 2%, Supplementary S8), and substrate-induced respiration response range (functional profile: 1%, Supplementary S8). Active microbial biomass effects on microbial functions were consistent by increasing all functions (Fig. 3B, Supplementary S8).

### Soil microbial facets interact in mediating microbial respiration

**Fig. 4:**
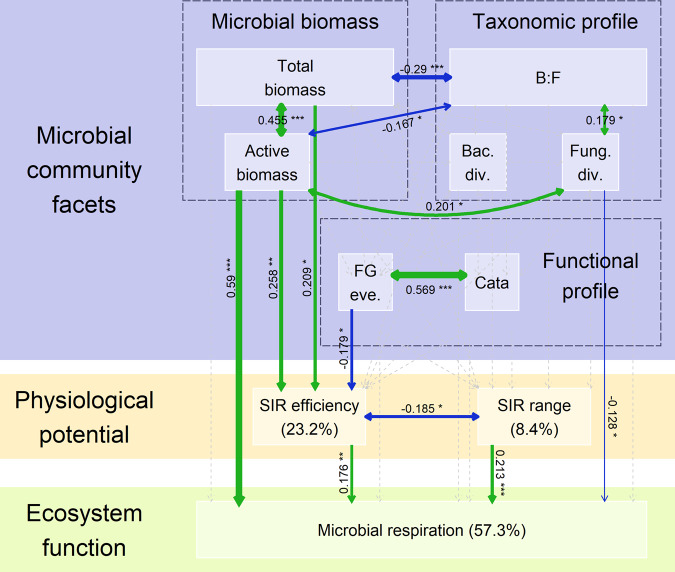
Structural equation model based on the effects of microbial community facets (i.e., microbial biomass: “Total biomass” and active microbial biomass, “Active biomass”; and, taxonomic profile: bacteria to fungi ratio, “B:F”; bacterial and fungal Shannon diversity, “Bac. div.” and “Fung. div.” respectively), genetic profile (i.e., carbon catabolism functional genes abundance: “Cata”, and evenness: “FG eve.”), and physiological potential (i.e., substrate-induced respiration efficiency and response range: “SIR efficiency” and “SIR range”) on ecosystem function (i.e., “Microbial respiration”). Correlations between nodes are drawn with double-headed arrows, while causal relations were drawn with one-way arrows and are based on hypotheses explained in the main text; arrow widths are sized by the absolute effect size. Green and blue arrows stand for positive and negative relations between nodes, respectively, and significant relations between nodes are drawn with full lines, while non-significant relations are displayed with dashed lines, and the significance levels were standardized (“.”: *p*-value < 0.1., “*”: *p*-value < 0.05, “**”: *p*-value <0.01, and “***”: *p*-value < 0.001). For each endogenous variable (i.e., response variable), the part of variance explained (*R*^2^, in %) was added after the variable name.

We tested the combined effects of soil microbial biomass, taxonomic and functional profiles on microbial physiological potential and respiration using an SEM framework. The addition of microbial physiological potentials (“*R*^2^ with”) improved the variance explained of microbial respiration compared to the model considering microbial biomass and taxonomic and functional profile only (*R*^2^_with_ = 57% in Fig. 4 vs. *R*^2^_without_ = 50% in Fig. 3B). There were combined positive effects of microbial biomass, fungal diversity, and physiological potential on microbial respiration (active microbial biomass effect: estimate ± SE = 0.590 ± 0.060, *p*-value <  0.001; fungi diversity: 0.128 ± 0.058, *p*-value = 0.027; SIR efficiency: 0.176 ± 0.062, *p*-value = 0.005; SIR range: 0.213 ± 0.057, *p*-value < 0.001, Fig. 4, Supplementary S10). Soil microbial physiological potential, especially SIR efficiency, was strongly affected by soil microbial biomass and functional profile (total microbial biomass effect: 0.209 ± 0.083, *p*-value = 0.012; active microbial biomass: 0.258 ± 0.082, *p*-value = 0.002; and functional genes evenness: −0.179 ± 0.089, *p*-value = 0.045, Fig. 4, Supplementary S10).

The total effect size (i.e., sum of effects) of soil microbial biomass on microbial respiration was 0.672 (direct effect = 0.590, indirect effect = 0.082), while the total effect size of microbial taxonomic profile was 0.128 (only direct effect = 0.128), that of functional profile 0.031 (only indirect = 0.031), and that of physiological potential was 0.389 (only direct effects). Overall, we observed a strong effect of microbial biomass (i.e., a quantity-related measure, total effect: 0.672), but minor to neutral effects of microbial diversity (i.e., diversity measures, total effect of taxonomic and functional diversity: 0.159).

### Soil quality shapes the relationship between the soil microbial community and microbial functions

**Fig. 5:**
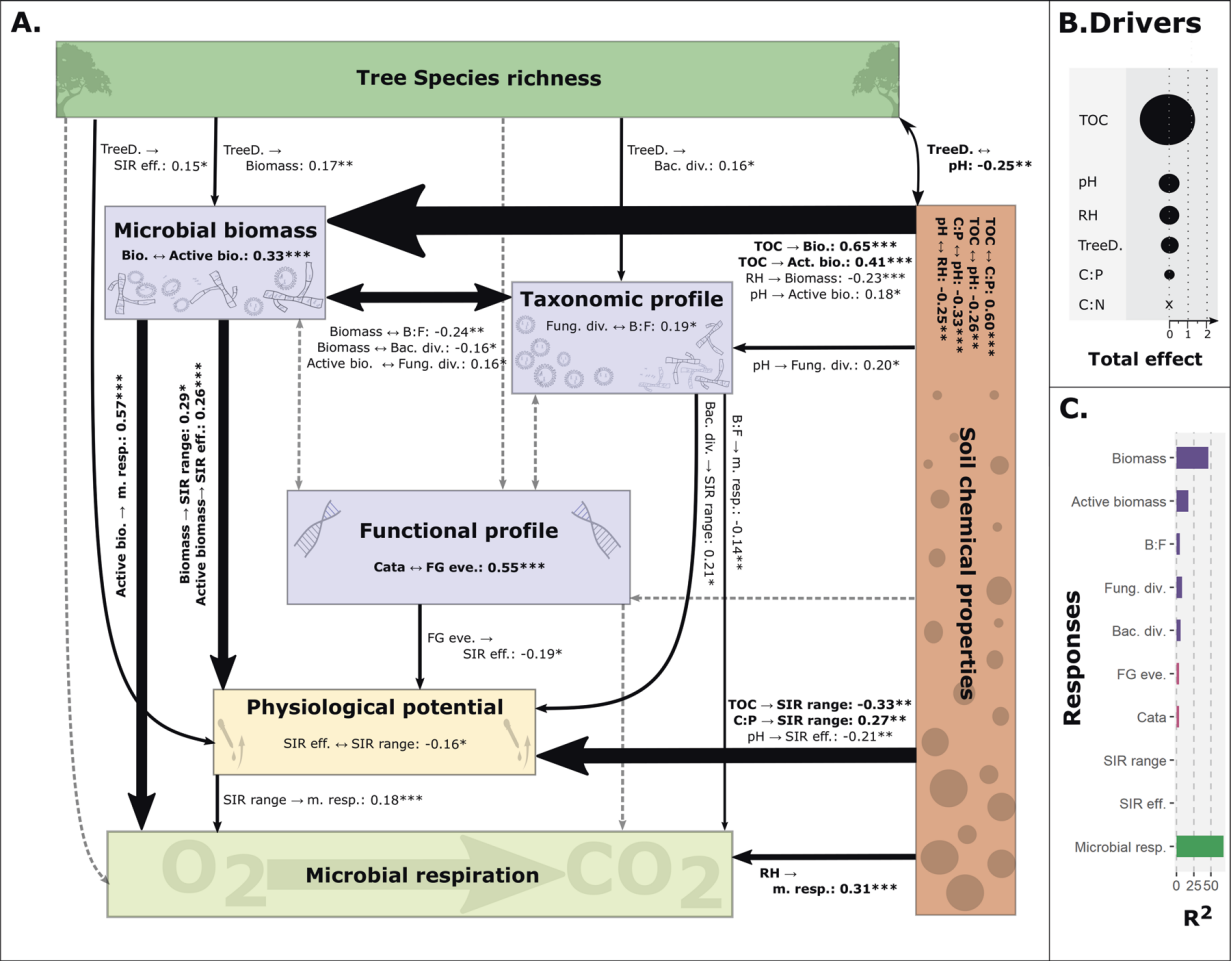
Structural equation model based on the effects of soil chemical properties and tree species richness on microbial community—ecosystem functioning linkages. **A** Structural equation model summary. Each node represents a group of variables, and each arrow summarizes all the significant effects between all the variables of two nodes. Correlations between nodes are drawn with double-headed arrows, while causal relations are drawn with simple arrows; arrow widths are sized by the sum of the absolute standardized effect size of significant relations between all variables of the two nodes. When no significant relations were found between any variables of two nodes, the arrows are drawn with dashed lines. Significant relationships between variables were specified in the figure (“.”: *p*-value < 0.1., “*”: *p*-value < 0.05, “**”: *p*-value <0.01, and “***”: *p*-value < 0.001). **B** Total effects of soil chemical properties and tree diversity (“Drivers”) on soil microbial facets and functions. The total effect size of the exogenous variables (i.e., tree species richness: “TreeD”, total organic carbon: “TOC”, soil pH: “pH”, soil relative humidity: “RH”, soil carbon to phosphorus ratio: “C:P”, and soil carbon to nitrogen ratio: “C:N”) on the microbial community facets (i.e., total microbial biomass: “Bio”, active microbial biomass: “Active bio.”, bacterial and fungal Shannon diversity: “Bac. div” and “Fung. div.”, bacteria to fungi ratio: “B:F”, catabolism functional genes abundance and evenness: “Cata” and “FG eve.”) et functions (substrate-induced respiration efficiency and response range: “SIR eff.” and “SIR range”, and microbial respiration: “m. resp.”) are shown by circles sized according to the sum of absolute standardized effect sizes. **C** Model explanatory power. *R*^2^ values of response variables (*y*-axis) for the model are displayed on the *x*-axis. See Supplementary S11 for more details.

The addition of tree diversity and soil chemical properties to our model increased the explained variance of microbial respiration (*R*^2^_with_ = 68% in Fig. 5 vs. *R*^2^_without_ = 57% in Fig. 4) and explained part of soil microbial biomass variance (*R*^2^_microbial biomass_ = 46% Fig. 5, Supplementary S11). Soil chemical properties (i.e., soil carbon, nitrogen, and phosphorus contents, soil pH, and humidity) affected all soil microbial properties and their interrelationships (microbial biomass, taxonomic and functional profiles, physiological potential, and microbial respiration) with the strongest effect on soil microbial biomass (total effect on microbial biomass: 1.474, total effect on taxonomic profile: 0.199, no effect on functional profile, total effect on physiological potential: 0.799, total effect on microbial respiration: 0.312; Fig. 5, Supplementary S11). TOC was the most important aspect of soil quality with a total effect of 1.383, while the total effect of all other soil properties together reached 1.400 (Fig. 5). Moreover, TOC and pH affected most of the microbial facets, while the other soil chemical properties affected only one or a few of the microbial facets (Fig. 5). For example, soil humidity increased microbial respiration but decreased total microbial biomass (0.312 ± 0.054, *p*-value < 0.001 and −0.234, *p*-value < 0.001, respectively); while, carbon to phosphorus ratio only increased SIR range (0.269 ± 0.098, *p*-value = 0.006, Fig. 5, Supplementary S11).

### Tree diversity effects on soil microbial respiration are mediated by the microbial community facets

In addition, tree species richness affected soil microbial biomass and taxonomic profile, and the community physiological potential with a positive effect on total microbial biomass (0.173 ± 0.063, *p*-value = 0.006), bacterial diversity (0.164 ± 0.082, *p*-value = 0.045), and SIR efficiency (0.152 ± 0.073, *p*-value = 0.038, Fig. 5, Supplementary S11). By increasing microbial biomass and physiological potential, tree species richness indirectly increased microbial respiration (indirect effect: 0.014).

## Discussion

Our results show a positive effect of tree diversity on the measured soil microbial community facets and functions (H1). By integrating soil microbial biomass, taxonomic and functional profiles into a single framework, our analyses show how these different facets of the soil microbial community are linked to each other (H2) and mediate the effect of tree diversity and soil chemical properties on microbial respiration (H3–H4). Our results highlight that soil microbial biomass and physiological potential are the main drivers of microbial respiration (H3). In turn, the microbial physiological potential is strongly affected by microbial biomass and functional gene evenness. Our results suggest that the relationship between soil microbial facets and realized functions are dependent on soil biochemistry. Taken together, our study presents a comprehensive framework of tree diversity effects on microbial community facets and functioning, providing novel insights into the most crucial variables for modeling changes in microbe-driven ecosystem functioning. For example, focusing our future investigations on tree species richness, soil carbon content, pH, and moisture will allow us to better predict soil microbial biomass as well as functioning.

### Soil microbial community facets drives soil microbial functions

Our analyses showed strong positive effects of active microbial biomass and the functional gene evenness on microbial physiological potential and microbial respiration, as expected based on previous studies [11, 19, 37]. Increasing microbial biomass per se increases the number of cells processing substrates and breathing, which results in enhanced total microbial respiration. We found that fungal diversity reduced microbial respiration, which contrasts with previous findings which suggest a strong positive effect of fungal diversity on microbial respiration [8]. Potentially, high fungal diversity coincided with or was related to low availability of easily degradable substrates and dominance of more recalcitrant carbon sources [67], but see [68].

In addition, we found that microbial physiology had a positive effect on microbial respiration by mediating functional gene evenness and part of microbial biomass effects on microbial respiration. Substrate-induced respiration methods like MicroResp.^®^ introduce to the microbial community a range of substrates which target different oxidation pathways [63, 67] in order to quantify the community’s physiological profile [65]. This method provides an overview of the microbial community potential under resource-rich conditions, and may also not adequately reflect microbial respiration in situ, where different oxidation pathways may not be evenly activated. However, in longer physiological processes, such as litter decomposition, where litter chemical composition is changing with time [69, 70], several oxidation pathways are successively activated. Therefore, information on the community’s potential to evenly cover a large range of physiological pathways (i.e., provided by MicroResp^®^ measurements) may become critical.

By bringing together the different facets of the microbial community, we showed the complementary effects of these microbial community facets on microbial realized functions, the significance of microbial biomass to explain microbial respiration, and the mediation of microbial community facets effects on microbial respiration by the microbial physiological potential. This new insight on the links between microbial community facets and realized functions would now need to be considered in future efforts to model microbial processes in soils [46, 49, 71].

### Soil chemical properties drive the soil microbial community—microbial functions relationships

We found that soil chemical properties were the strongest drivers of linkages between the soil microbial community and soil functioning by affecting all facets of the microbial community and microbial respiration. Soil organic carbon content had strong positive effects on both microbial biomass and microbial physiological potential, while soil pH affected microbial biomass, taxonomic profile and physiological potential; however, the soil chemical properties (i.e., soil carbon to phosphorus ratio, and soil humidity) had less pronounced effects on fewer facets. For example, soil humidity decreased microbial biomass but increased microbial respiration, while soil C:P ratio only increased substrate-induced respiration response range. These inconsistent effects of soil chemistry on the different facets of the microbial community were expected from previous studies showing different soil variables and selection mechanisms for microbial taxonomic and functional profiles e.g., [8, 36, 37]. However, our analyses highlighted soil carbon content as the main driver of the microbial community, affecting microbial biomass, taxonomic profiles, and physiological potential. Together, these effects enhanced microbial respiration. The major significance of soil carbon in structuring soil microbial communities is well known and supported by many previous local- e.g., [41, 44] to global-scale studies e.g., [12, 46].

Consequently, one might expect a negative feedback effect of soil microbial respiration on organic carbon content, due to the increase of soil carbon mineralization by the microbial community. However, high microbial respiration and microbial biomass are two strong indicators of microbial transformation of plant residues and soil organic carbon to microbial necromass [4, 19, 20, 27, 72]. This transformation of easily decomposable plant material to microbial necromass may increase soil carbon residency time, and therefore soil carbon storage [49]. Our results provide novel insights on a positive tree diversity-induced feedback of soil carbon content on soil carbon storage by increasing soil microbial biomass and functioning. However, further empirical and theoretical studies are needed to mechanically test the effects of soil carbon chemical pools on soil bioprocesses as well as soil carbon sequestration. This requires a better description and measurement of the soil carbon chemical pools [49, 72]. Furthermore, mechanistic and dynamic models need to be built and calibrated on temporal data to predict soil carbon dynamics [49, 71], and to consider the context-dependency of the microbial processes to biotic and abiotic environmental conditions [44, 71, 73, 74].

### Tree diversity effects on soil respiration mediated via changes in the soil microbial community

We observed a positive effect of tree species richness on the different facets of the microbial community and its functions. Our results demonstrate that tree species richness drives soil microbial functions, such as microbial respiration, by modifying the soil microbial community: microbial biomass and diversity. Such positive effects of tree diversity on microbial biomass were shown in the past across biomes. They were explained by an increase of tree productivity and thus of tree carbon release into the soil e.g., root exudation, [21], litter production, [22, 75]. Additionally, tree diversity is expected to increase substrate diversity available to soil microorganisms [21, 25, 76, 77]. Such an increase in substrate diversity could explain the enhancement of substrate-induced respiration efficiency observed by selecting microbial communities adapted to diverse substrate inputs [78]. These results suggest a double effect of tree diversity on the microbial community. On the one hand, tree diversity maintains higher microbial biomass by increasing tree productivity and carbon inputs into the soil. On the other hand, tree diversity increases the heterogeneity of the organic inputs [79], and maintains a higher level of functioning by increasing microbial physiological potential. In this study, the positive effect of tree diversity on microbial respiration was mostly driven by enhanced microbial biomass.

## Conclusion

In conclusion, we showed that tree diversity and soil carbon content drive microbial respiration through their effects on the different soil microbial community facets. We identified microbial biomass as the main predictor of microbial respiration, by incorporating the different soil microbial community facets and their drivers in a common framework. These results suggest a positive tree diversity-induced feedback of soil carbon content on soil carbon storage by increasing soil microbial biomass and respiration. These novel insights should be considered in efforts to model soil carbon dynamics and feedbacks to atmospheric carbon concentrations [46] as well as the ecosystem consequences of reforestation approaches [80–83].

## Supplementary information

supplemental-data S1

supplemental-data S2

supplemental-data S3

supplemental-data S4

supplemental-data S5

supplemental-data S6

supplemental-data S7

supplemental-data S8

supplemental-data S9

supplemental-data S10

supplemental-data S11

## References

[1] Davidson EA, Janssens IA (2006). Temperature sensitivity of soil carbon decomposition and feedbacks to climate change. Nature.

[2] Stocker TF, et al. IPCC, 2013: Climate Change 2013: The Physical Science Basis. Contribution of Working Group I to the Fifth Assessment Report of the Intergovernmental Panel on Climate Change. Cambridge University Press, Cambridge, United Kingdom and New York, NY, USA; 2013.

[3] Lal R (2004). Soil carbon sequestration impacts on global climate change and food security. Science.

[4] Trumbore SE (1997). Potential responses of soil organic carbon to global environmental change. Proc Natl Acad Sci USA.

[5] Schlesinger WH, Andrews JA (2000). Soil respiration and the global carbon cycle. Biogeochemistry.

[6] Singh BK, Bardgett RD, Smith P, Reay DS (2010). Microorganisms and climate change: terrestrial feedbacks and mitigation options. Nat Rev Microbiol.

[7] Delgado-Baquerizo M, Maestre FT, Reich PB, Jeffries TC, Gaitan JJ, Encinar D (2016). Microbial diversity drives multifunctionality in terrestrial ecosystems. Nat Commun.

[8] Liu Y-R, Delgado-Baquerizo M, Wang JT, Hu HW, Yang Z, He JZ. New insights into the role of microbial community composition in driving soil respiration rates. Soil Biol Biochem. 2018;118:35–41. 10.1016/j.soilbio.2017.12.003.

[9] McGuire KL, Treseder KK. Microbial communities and their relevance for ecosystem models: Decomposition as a case study. Soil Biol Biochem. 2010;42:529–35. 10.1016/j.soilbio.2009.11.016.

[10] Monson RK, Lipson DL, Burns SP, Turnipseed AA, Delany AC, Williams MW (2006). Winter forest soil respiration controlled by climate and microbial community composition. Nature.

[11] Wieder WR, Bonan GB, Allison SD. Global soil carbon projections are improved by modelling microbial processes. Nature Clim Change. 2013;3:909–12. 10.1038/nclimate1951.

[12] Delgado‐Baquerizo M, Maestre FT, Reich PB, Trivedi P, Osanai Y, Liu YR (2016). Carbon content and climate variability drive global soil bacterial diversity patterns. Ecol Monogr.

[13] Maaroufi NI, Long JR de. Global change impacts on forest soils: linkage between soil biota and carbon-nitrogen-phosphorus stoichiometry. Front For Glob Change. 2020;3. 10.3389/ffgc.2020.00016.

[14] Gottschall F, Davids S, Newiger-Dous TE, Auge H, Cesarz S, Eisenhauer N (2019). Tree species identity determines wood decomposition via microclimatic effects. Ecol Evol.

[15] Durán J, Delgado-Baquerizo M (2020). Vegetation structure determines the spatial variability of soil biodiversity across biomes. Sci Rep.

[16] Beugnon R, et al. Abiotic and biotic drivers of scale-dependent tree trait effects on soil microbial biomass and soil carbon concentration (in press).

[17] Pei Z, Eichenberg D, Bruelheide H, Kröber W, Kühn P, Li Y, et al. Soil and tree species traits both shape soil microbial communities during early growth of Chinese subtropical forests. Soil Biol Biochem. 2016;96:180–90. 10.1016/j.soilbio.2016.02.004.

[18] Xu S, Eisenhauer N, Ferlian O, Zhang J, Zhou G, Lu X (2020). Species richness promotes ecosystem carbon storage: evidence from biodiversity-ecosystem functioning experiments. Proc Biol Sci.

[19] Lange M, Eisenhauer N, Sierra CA, Bessler H, Engels C, Griffiths RI (2015). Plant diversity increases soil microbial activity and soil carbon storage. Nat Commun.

[20] Schmidt MW, Torn MS, Abiven S, Dittmar T, Guggenberger G, Janssens IA (2011). Persistence of soil organic matter as an ecosystem property. Nature.

[21] Eisenhauer N, Lanoue A, Strecker T, Scheu S, Steinauer K, Thakur MP (2017). Root biomass and exudates link plant diversity with soil bacterial and fungal biomass. Sci Rep.

[22] Huang Y, Ma Y, Zhao K, Niklaus PA, Schmid B, He JS (2017). Positive effects of tree species diversity on litterfall quantity and quality along a secondary successional chronosequence in a subtropical forest. J Plant Ecol.

[23] Fornara DA, Tilman D (2008). Plant functional composition influences rates of soil carbon and nitrogen accumulation. J Ecol.

[24] Chen C, Chen HYH, Chen X, Huang Z (2019). Meta-analysis shows positive effects of plant diversity on microbial biomass and respiration. Nat Commun.

[25] Thoms C, Gattinger A, Jacob M, Thomas FM, Gleixner G (2010). Direct and indirect effects of tree diversity drive soil microbial diversity in temperate deciduous forest. Soil Biol Biochem.

[26] Rousk J, Brookes PC, Bååth E. Investigating the mechanisms for the opposing pH relationships of fungal and bacterial growth in soil. Soil Biol Biochem. 2010;42:926–34. 10.1016/j.soilbio.2010.02.009.

[27] Miltner A, Bombach P, Schmidt-Brücken B, Kästner M (2012). SOM genesis: microbial biomass as a significant source. Biogeochemistry.

[28] Delgado-Baquerizo M, Reich PB, Khachane AN, Campbell CD, Thomas N, Freitag TE (2017). It is elemental: soil nutrient stoichiometry drives bacterial diversity. Environ Microbiol.

[29] Fanin N, Barantal S, Fromin N, Schimann H, Schevin P, Hättenschwiler S (2012). Distinct microbial limitations in litter and underlying soil revealed by carbon and nutrient fertilization in a tropical rainforest. PLoS ONE.

[30] Louca S, Parfrey LW, Doebeli M (2016). Decoupling function and taxonomy in the global ocean microbiome. Science.

[31] Cao J, Jia X, Pang S, Hu Y, Li Y, Wang Q (2020). Functional structure, taxonomic composition and the dominant assembly processes of soil prokaryotic community along an altitudinal gradient. Appl Soil Ecol.

[32] Bao Y, Guo Z, Chen R, Wu M, Li Z, Lin X (2020). Functional community composition has less environmental variability than taxonomic composition in straw-degrading bacteria. Biol Fertil Soils.

[33] Galand PE, Pereira O, Hochart C, Auguet JC, Debroas D (2018). A strong link between marine microbial community composition and function challenges the idea of functional redundancy. ISME J.

[34] Kuang J, Huang L, He Z, Chen L, Hua Z, Jia P (2016). Predicting taxonomic and functional structure of microbial communities in acid mine drainage. ISME J.

[35] Jurburg SD, Salles JF. Functional Redundancy and Ecosystem Function—The Soil Microbiota as a Case Study. In: Lo Y-H, Blanco JA, Roy S, editors. Biodiversity in Ecosystems—Linking Structure and Function. Rijeka, Croatia, InTech; 2015.

[36] Chen Q-L, Ding J, Li CY, Yan ZZ, He JZ, Hu HW (2020). Microbial functional attributes, rather than taxonomic attributes, drive top soil respiration, nitrification and denitrification processes. Sci Total Environ.

[37] Trivedi P, Delgado-Baquerizo M, Trivedi C, Hu H, Anderson IC, Jeffries TC (2016). Microbial regulation of the soil carbon cycle: evidence from gene-enzyme relationships. ISME J.

[38] Hale L, Feng W, Yin H, Guo X, Zhou X, Bracho R (2019). Tundra microbial community taxa and traits predict decomposition parameters of stable, old soil organic carbon. ISME J.

[39] Chen J, Sinsabaugh RL (2021). Linking microbial functional gene abundance and soil extracellular enzyme activity: Implications for soil carbon dynamics. Glob Change Biol.

[40] Allison SD, Wallenstein MD, Bradford MA. Soil-carbon response to warming dependent on microbial physiology. Nat Geosci. 2010;3:336–40. 10.1038/ngeo846.

[41] Eisenhauer N, Bessler H, Engels C, Gleixner G, Habekost M, Milcu A (2010). Plant diversity effects on soil microorganisms support the singular hypothesis. Ecology.

[42] Bonner MT, Shoo LP, Brackin R, Schmidt S (2018). Relationship between microbial composition and substrate use efficiency in a tropical soil. Geoderma.

[43] Bárány A, Szili-Kovács T, Krett G, Füzy A, Márialigeti K, Borsodi AK (2014). Metabolic activity and genetic diversity of microbial communities inhabiting the rhizosphere of halophyton plants. Acta Microbiol Immunol Hung.

[44] Chodak M, Klimek B, Niklińska M (2016). Composition and activity of soil microbial communities in different types of temperate forests. Biol Fertil Soils.

[45] Lagomarsino A, Knapp BA, Moscatelli MC, De Angelis P, Grego S, Insam H. Structural and functional diversity of soil microbes is affected by elevated [CO2] and N addition in a poplar plantation. J Soils Sediments. 2007;7:399–405. 10.1065/jss2007.04.223.

[46] Crowther TW, et al. The global soil community and its influence on biogeochemistry. Science. 2019;365. 10.1126/science.aav0550. 10.1126/science.aav0550 31439761

[47] Hall EK, Bernhardt ES, Bier RL, Bradford MA, Boot CM, Cotner JB (2018). Understanding how microbiomes influence the systems they inhabit. Nat Microbiol.

[48] Malik AA, Martiny J, Brodie EL, Martiny AC, Treseder KK, Allison SD (2020). Defining trait-based microbial strategies with consequences for soil carbon cycling under climate change. ISME J.

[49] Sainte-Marie J, Barrandon M, Saint-André L, Gelhaye E, Martin F, Derrien D (2021). C-STABILITY an innovative modeling framework to leverage the continuous representation of organic matter. Nat Commun.

[50] Bruelheide H, Nadrowski K, Assmann T, Bauhus J, Both S, Buscot F (2014). Designing forest biodiversity experiments: general considerations illustrated by a new large experiment in subtropical C hina. Methods Ecol Evol.

[51] Yu G, Chen Z, Piao S, Peng C, Ciais P, Wang Q (2014). High carbon dioxide uptake by subtropical forest ecosystems in the East Asian monsoon region. Proc Natl Acad Sci USA.

[52] Bradstreet RB (1954). Determination of Nitro Nitrogen by Kjeldahl Method. Anal Chem.

[53] Frostegård Å, Tunlid A, Bååth E. Microbial biomass measured as total lipid phosphate in soils of different organic content. J Microbiol Methods. 1991;14:151–63. 10.1016/0167-7012(91)90018-L.

[54] Ruess L, Chamberlain PM. The fat that matters: Soil food web analysis using fatty acids and their carbon stable isotope signature. Soil Biol Biochem. 2010;42:1898–910. 10.1016/j.soilbio.2010.07.020.

[55] Scheu S (1992). Automated measurement of the respiratory response of soil microcompartments: active microbial biomass in earthworm faeces. Soil Biol Biochem.

[56] Schöps R, Goldmann K, Herz K, Lentendu G, Schöning I, Bruelheide H (2018). Land-use intensity rather than plant functional identity shapes bacterial and fungal rhizosphere communities. Front Microbiol.

[57] Nawaz A, et al. DNA- and RNA- Derived Fungal Communities in Subsurface Aquifers Only Partly Overlap but React Similarly to Environmental Factors. Microorganisms. 2019;7. 10.3390/microorganisms7090341. 10.3390/microorganisms7090341 PMC6780912 31514383

[58] Bolyen E, Rideout JR, Dillon MR, Bokulich NA, Abnet CC, Al-Ghalith GA (2019). Reproducible, interactive, scalable and extensible microbiome data science using QIIME 2. Nat Biotechnol.

[59] Cutadapt removes adapter sequences from high-throughput sequencing reads. EMBnet j. 2011;17:10. 10.14806/ej.17.1.200.

[60] Callahan BJ, McMurdie PJ, Rosen MJ, Han AW, Johnson AJ, Holmes SP (2016). DADA2: High-resolution sample inference from Illumina amplicon data. Nat Methods.

[61] McMurdie PJ, Holmes S (2013). phyloseq: an R package for reproducible interactive analysis and graphics of microbiome census data. PLoS ONE.

[62] Lahti L, Shetty S, Blake T, Salojarvi J. Microbiome R package. Tools Microbiome Anal R. 2017;1:504.

[63] Liang Y, Liu X, Singletary MA, Wang K, Mattes TE (2017). Relationships between the Abundance and Expression of Functional Genes from Vinyl Chloride (VC)-Degrading Bacteria and Geochemical Parameters at VC-Contaminated Sites. Environ Sci Technol.

[64] Zheng B, Zhu Y, Sardans J, Peñuelas J, Su J (2018). QMEC: a tool for high-throughput quantitative assessment of microbial functional potential in C, N, P, and S biogeochemical cycling. Sci China Life Sci.

[65] Campbell CD, Chapman SJ, Cameron CM, Davidson MS, Potts JM (2003). A rapid microtiter plate method to measure carbon dioxide evolved from carbon substrate amendments so as to determine the physiological profiles of soil microbial communities by using whole soil. Appl Environ Microbiol.

[66] Rosseel Y (2012). Lavaan: An R package for structural equation modeling and more. Version 0.5–12 (BETA). J Stat Softw.

[67] Paterson E, Osler G, Dawson LA, Gebbing T, Sim A, Ord B. Labile and recalcitrant plant fractions are utilised by distinct microbial communities in soil: Independent of the presence of roots and mycorrhizal fungi. Soil Biol Biochem. 2008;40:1103–13. 10.1016/j.soilbio.2007.12.003.

[68] Kramer S, Dibbern D, Moll J, Huenninghaus M, Koller R, Krueger D (2016). Resource Partitioning between Bacteria, Fungi, and Protists in the Detritusphere of an Agricultural Soil. Front Microbiol.

[69] Berg B (2000). Litter decomposition and organic matter turnover in northern forest soils. Forest Ecol Manag.

[70] Moretto AS, Distel RA, Didoné NG (2001). Decomposition and nutrient dynamic of leaf litter and roots from palatable and unpalatable grasses in a semi-arid grassland. Appl Soil Ecol.

[71] Kyker-Snowman E, Wieder WR, Frey SD, Grandy AS (2020). Stoichiometrically coupled carbon and nitrogen cycling in the MIcrobial-MIneral Carbon Stabilization model version 1.0 (MIMICS-CN v1.0). Geosci Model Dev.

[72] Buckeridge KM, Mason KE, McNamara NP, Ostle N, Puissant J, Goodall T (2020). Environmental and microbial controls on microbial necromass recycling, an important precursor for soil carbon stabilization. Commun Earth Environ.

[73] Cesarz S, Craven D, Auge H, Bruelheide H, Castagneyrol B, Hector A, et al.. Biotic and abiotic drivers of soil microbial functions across tree diversity experiments. bioRXiv 2020. 10.1101/2020.01.30.927277.

[74] Tedersoo L, Bahram M, Cajthaml T, Põlme S, Hiiesalu I, Anslan S (2016). Tree diversity and species identity effects on soil fungi, protists and animals are context dependent. ISME J.

[75] Huang Y, Chen Y, Castro-Izaguirre N, Baruffol M, Brezzi M, Lang A (2018). Impacts of species richness on productivity in a large-scale subtropical forest experiment. Science.

[76] Chapman SK, Newman GS, Hart SC, Schweitzer JA, Koch GW (2013). Leaf litter mixtures alter microbial community development: mechanisms for non-additive effects in litter decomposition. PLoS ONE.

[77] Eisenhauer N, Dobies T, Cesarz S, Hobbie SE, Meyer RJ, Worm K (2013). Plant diversity effects on soil food webs are stronger than those of elevated CO2 and N deposition in a long-term grassland experiment. Proc Natl Acad Sci USA.

[78] Brandt BW, Kelpin FDL, van Leeuwen IMM, Kooijman SALM (2004). Modelling microbial adaptation to changing availability of substrates. Water Res.

[79] Hooper DU, BIGNELL DE, BROWN VK, BRUSSARD L, MARK DANGERFIELD J, WALL DH (2000). Interactions between Aboveground and Belowground Biodiversity in Terrestrial Ecosystems: Patterns, Mechanisms, and Feedbacks. BioScience.

[80] Domke GM, Oswalt SN, Walters BF, Morin RS (2020). Tree planting has the potential to increase carbon sequestration capacity of forests in the United States. Proc Natl Acad Sci USA.

[81] Tong X, Brandt M, Yue Y, Ciais P, Rudbeck Jepsen M, Penuelas J (2020). Forest management in southern China generates short term extensive carbon sequestration. Nat Commun.

[82] Veldkamp E, Schmidt M, Powers JS, Corre MD (2020). Deforestation and reforestation impacts on soils in the tropics. Nat Rev Earth Environ.

[83] Lewis SL, Wheeler CE, Mitchard ETA, Koch A (2019). Restoring natural forests is the best way to remove atmospheric carbon. Nature.

