# Supplementary material for: Tree diversity and soil chemical properties drive the linkages between soil microbial community and ecosystem functioning
Source: ISME Commun. 2021 Aug 23;1:41. doi: 10.1038/s43705-021-00040-0 (PMC9723754; doi:10.1038/s43705-021-00040-0)
Supplement: Supplementary file 1 — supplemental-data S1 [file 43705_2021_40_MOESM1_ESM.docx]

# Materials and methods

## Study site, study design, and sampling

Our study site was located in south-east China in the Jiangxi province (29.08-29.11° N, 117.90-117.93° E). The region is characterized by a subtropical climate with warm, rainy summers and cold, dry winters (mean temperature of 16.7°C and mean rainfall of 1821 mm) [1]. Soils in the region are Cambisols and Cambisol derivatives, with Regosol on ridges and crests [2]. The natural vegetation consists of species-rich broad-leaved forests dominated by *Quercus glauca, Castanopsis eyrei, Daphniphyllum oldhamii,* and *Lithocarpus glaber* [3, 4]. Sampling took place in BEF China, a tree diversity experiment, including tree species mixture plots (1, 2, 4, 8, and 16 tree species per plot), was planted in 2009 after clear-cutting the original forest (Fig. 1) [4]. To account for the role of tree diversity and soil quality, we collected 150 soil samples across different levels of tree diversity randomly distributed in the landscape (Fig. 1, Suppl. S2). We sampled from mid-August to late-September 2018, before the litterfall season. To avoid spatio-temporal autocorrelation, the daily sample location was chosen randomly, and to control for the distance to the trees, each sample was extracted on the transect between two trees. For each pair of trees, we extracted four soil cores (5 cm diameter; 10 cm depth), 5 cm and 20 cm away from the centerpoint between the tree pair (Fig. 1). A composite sample was built from these four cores by homogenizing with a 2 mm sieve.

## Soil quality analyses

Soil moisture was measured from 25 g of soil by drying at 40°C for two days. A subsample was used to measure soil pH in a 1:2.5 soil-water solution. Soil total organic carbon (TOC) was measured by a TOC Analyzer (Liqui TOC II; Elementar Analysensysteme GmbH, Hanau, Germany). Soil total nitrogen (TN) was measured on an auto-analyzer (SEAL Analytical GmbH, Norderstedt, Germany) using the Kjeldahl method [5]. Soil total phosphorus (TP) concentration was measured after wet digestion with H_2_SO_4_ and HClO_4_ by a UV-VIS spectrophotometer (UV2700, SHIMADZU, Japan). Carbon to nitrogen and carbon to phosphorus ratios were calculated as TOC:TN and TOC:TP, respectively.

## Soil microbial biomass

Microbial biomass was measured using phospholipid fatty acid (PLFA) analysis. PLFAs were extracted from 5 g of frozen soil following Frostegård et al. (1991) [6]. Biomarkers were assigned to microbial functional groups according to Ruess et al. (2010) [7]. These markers targeted bacteria (gram-positive bacteria: i15:0, a15:0, i16:0, i17:0; gram-negative bacteria: cy17:0, cy19:0; general bacterial markers: 16:1ω5; 16:1ω7), arbuscular mycorrhizal fungi (20:1ω9), and saprophytic and ectomycorrhizal association fungi (18:1ω9 and 18:2ω6,9, see Suppl. S3). Total microbial biomass was calculated as the sum of biomasses of all microbial groups. The ratio of bacteria to fungi (B:F) was calculated as the ratio of the sum of all bacterial biomasses to the sum of all fungal biomasses.

Active microbial biomass was measured using the substrate-induced respiration method [8]. About 6 g of soil was used to determine soil active microbial biomass, and 8 mg of glucose per gram of dry soil was added to saturated the soil micro-organism catabolism enzymes. O_2_ respiration was measured based on electrolyte O_2_ micro-compensation using an automated respirometer. Active microbial biomass was calculated from the maximum initial respiratory response after induction (MIRR).

## Soil microbial taxonomic profile

Microbial DNA was extracted from freeze-dried soil samples using a PowerSoil DNA Isolation Kit (MO BIO Laboratories Inc., Carlsbad, CA, United States). DNA concentrations were checked with a NanoDrop spectrophotometer (Thermo Fisher Scientific, Dreieich, Germany), and the extracts were adjusted to 10–15 ng/ul. The bacterial and fungal amplicon libraries were prepared following Schöps et al. (2018) [9] and Nawaz et al. (2019) [10]. Briefly, bacterial and fungal amplicon libraries were built separately using 16S rRNA gene and ITS2 rDNA regions, respectively. The bacterial 16S rRNA gene was amplified with universal primers 515f and 806r [11] with Illumina adapter sequence overhangs. The fungal ITS2 rDNA region was amplified by performing a semi-nested PCR using the initial primer combination of ITS1F [12] and ITS4 [13]hr followed by the primer pair fITS7 [14] and ITS4 containing the Illumina adapter sequences. The amplicon libraries were indexed, purified, quantified, and pooled equimolarly to a final concentration of 4nM which was then mixed in 1:3 ratio to make the final sequencing library. Paired-end sequencing of 2x300 bp was performed on an Illumina MiSeq platform (Illumina Inc., San Diego, CA, United States) using the MiSeq Reagent kit v3 at the Department of Environmental Microbiology, UFZ.

Bioinformatic analysis was performed using the Quantitative Insights into Microbial Ecology – QIIME 2 2020.2 [15]. The forward and reverse reads were demultiplexed, primer sequences were trimmed, denoised, and grouped into Amplicon Sequence Variants (ASVs) using cut-adapt for chimeria removal (q2-cutadapt) [16] and DADA2 for non-target taxa removal (via q2‐dada2) [17]. ASV tables were imported into R with the 'phyloseq' package [18]. The fungal and bacterial ASVs were rarefied to 16,542 and 28,897 reads per sample respectively. OTU richness, Shannon diversity, and Pielou evenness were calculated using the 'microbiome' package [19]. We inspected the correlations between these indices and focused our analyses on Shannon diversity index (Suppl. S4).

## Soil microbial functional profile

DNA was extracted with the FastDNA Spin Kit for Soil (MP Biomedicals, USA) following the manufacturer's instructions. DNA concentrations were checked with a NanoDrop spectrophotometer (Thermo Fisher Scientific, Dreieich, Germany), and DNA concentrations were quantified with the QuantiFluor dsDNA kit (Promega, USA) and a microplate reader (SpectraMax M5, Molecular Devices). DNA was diluted to 50 ng µl^−1^ with sterile water and stored at −20 °C.

Microbial functional genes coding for enzymes involved in carbon anabolism and catabolism processes, which are central to soil carbon cycling (complete list in Suppl. S5) [20], were quantified using a high-throughput quantitative-PCR-based chip (HT-qPCR; SmartChip Real-time PCR system, WaferGen Biosystems, Fremont, USA). This chip contained 72 primer pairs: 36 designed pairs, 35 published pairs, and the bacterial 16S rRNA gene, which allows to quantify 72 DNA genes in parallel [21]. PCR reaction conditions were as follows: initial denaturation of 10 min at 95°C, and 40 cycles of denaturation at 95°C for 30 s, annealing 30 s at 58°C and extension at 72°C for another 30 s. The melting curve was automatically generated by the WaferGen software. Three replicates for each sample were analyzed. Results with multiple melting peaks or with amplification efficiencies less than 80% and over 120% were excluded. Only results with a threshold cycle (C_T_) less than 31 (the detection limit for this method) were used for further analysis. The relative copy number of each functional gene was calculated as shown in eq. 1 [22]. Then, the relative abundance of a given functional gene was defined as the proportion of the relative copy number of a functional gene to the relative copy number of the 16S rRNA gene.

Gene relative copy number (GR):   GR = (31-C_T_) x (10/3) / GR_16S_                      (1)

To compare abundance patterns across functional genes, we scaled each functional gene abundance between 0 and 1 across all samples using the z-transformation, and we summed the scaled abundance of functional genes related to carbon catabolism (i.e. "Cata", Suppl. S5). To quantify the evenness of the functional gene abundances, the functional gene Pielou evenness was calculated using the R 'diversity' from the 'vegan' package ("FG evenness", respectively).

## Soil microbial physiological potential

Microbial physiological potential indices were calculated from substrate-induced respiration assays using the Microresp.® method [23]. Fourteen substrates from three chemical classes (i.e. saccharides, amino-acid, and carboxylic acids) were selected to create a gradient of molecular weights (ranging from 89 to 221 g.mol^-1^ ), and a gradient of carbon oxidation states (ranging from -2 to 3 e^-^ , Suppl. S5). Ten g of soil was evenly distributed on the half of 96 deep-well plate and incubated at 25°C for five days. For each substrate, 30 mg of substrate per gram of soil water was added to three wells. CO_2_ production of the wells was fixed in agar – cresol red gel during the six following hours. Total CO_2_ production of the wells was measured by colorimetry using a photo-spectrometer. Two indices were calculated from these CO_2_ measurements: substrate-use efficiency and substrate-use range. Substrate-use efficiency was calculated as the Pielou evenness (from R 'diversity' function package 'vegan') of the CO_2_ production of all substrates. Substrate-use range was defined as the difference in CO2 production between oxalic acid and alanine, the two substrates on the upper and lower extremes of carbon oxidation. We performed sensitivity analyses to explore the effects of substrate selection on these indices, which showed that substrate selection did not alter our results and conclusions (Suppl. S6).

## Soil microbial respiration

Soil microbial respiration was measured on 6 g of fresh soil following Scheu *et al*. (1992) [8] without adding any substrate or water, thereby reflecting the actual respiration at the site. During 24 hours, O_2_ consumption was continuously measured using an automated respirometer based on electrolytic O_2_ micro-compensation [8]. Soil microbial respiration was calculated as the mean of O_2_ consumption between the 14 to 24 hours after starting the measurement. Active microbial biomass (with substrate addition) and microbial respiration (without substrate addition) were measured on the same sample and machine. To test the robustness of our results, all following analyses were run with and without active microbial biomass.

## Statistical analyses

All data handling and statistical analyses were performed using the R statistical software version 4.0.3, and all R scripts used for this study can be found in our GitHub repository (<https://github.com/remybeugnon/Beugnon-Du_et_al_2021_Microbial_community_and_functions>). All metrics inferred from soil measurements are summarized in the Suppl. S4. In order to avoid any model-fit deviation due to scale differences between variables, all explanatory variables were centered and divided by two standard deviations for our analyses using the R 'rescale' function from the 'arm' package. For each analysis, we compared the drivers' effect sizes defined as the standardized estimate of a given variable in the model where the response variable was centered and divided by two standard deviations.

### Tree diversity effects on soil microbial community facets and functions

We used linear multivariate models and normal distribution assumptions to test the effects of tree species richness on soil microbial biomass (total and active microbial biomass), taxonomic profile (B:F ratio and Shannon diversity of bacteria and fungi), functional profile (catabolic functional gene abundance and evenness), physiological potential (substrate-use efficiency and range), and microbial respiration. All previous linear multivariate models were tested in R using the ‘lm’ function and statistical hypotheses of the following linear models were tested in Suppl. S7 using the 'model_check' function from the 'performance' package in R.

### Effects of soil microbial facets on microbial functions

We tested the correlation between the microbial facets – soil microbial biomass, taxonomic and functional profiles – using Pearson correlation tests. We used linear multivariate models and normal distribution assumptions to test the effects of microbial biomass (total and active microbial biomass), taxonomic profile (B:F ratio and Shannon diversity of bacteria and fungi) and functional profile (catabolic functional gene abundance, and evenness) on soil microbial physiological potential (substrate-use efficiency and range), and soil microbial respiration. Explanatory variables (microbial biomasses, taxonomic and functional profile indices) were selected using forward and backward step selection based on AIC (i.e., R 'step' function from 'stats' package). A variance partitioning analysis was performed on the final set of variables to disentangle the effects of microbial biomass and taxonomic profile using the R 'varpart' function from the 'vegan' package. All previous linear multivariate models were tested in R using the ‘lm’ function and statistical hypotheses of the following linear models were tested in Suppl. S8 using the 'model_check' function from the 'performance' package in R

### Cascading effects of the different soil microbial community facets on microbial physiological potential and microbial respiration

We tested the relationships between soil microbial biomass, taxonomic and functional profiles, physiological potential, and respiration using a Structural Equation Modeling (SEM) framework. Microbial biomass, taxonomic and functional profiles were linked to each other by correlations, and their effects on physiological potential indices and soil microbial respiration were modeled with causal relations (directed paths). Our SEM was fitted using the R ‘sem’ function from the ‘lavaan’ package [24]. The model fit to our data, and model quality were estimated using three complementary indices: (i) the root mean square error of approximation (RMSEA), (ii) the comparative fit index (CFI), and (iii) the standardized root mean squared residuals (SRMR). Model fits were considered acceptable when RMSEA < 0.10, CFI > 0.9 and SRMR < 0.08. All statistical hypotheses and complete outputs can be found in Suppl. S9.

### Effects of tree species richness and soil quality on relationships between the soil microbial community and their functions

To test the effects of tree species richness and soil quality on the relationship between the soil microbial community facets and microbial respiration, we added the causal effects of soil quality indices and tree species richness onto the variables of our previous SEM model. To assess which group of response variables was the most affected by soil quality and tree species richness, the effects of soil quality and tree species richness were summarized by a group of response variables (soil microbial biomass, taxonomic profile, functional profile, physiological potential, and microbial respiration). For each group of response variables, we summed all the absolute standardized effects of soil quality or tree species richness on each of the response variables. Additionally, to assess the importance of soil quality indices and tree species richness for microbial community facets and microbial functions, we summed the absolute standardized effects of each soil quality index and tree species richness. All statistical hypotheses and complete outputs can be found in Suppl. S10.

References

[1] Yang X*, et al.* Establishment success in a forest biodiversity and ecosystem functioning experiment in subtropical China (BEF-China). Eur J Forest Res 2013; 132(4): 593–606
[https://doi.org/10.1007/s10342-013-0696-z]

[2] Geißler C*, et al.* Splash erosion potential under tree canopies in subtropical SE China. CATENA 2012; 91: 85–93
[https://doi.org/10.1016/j.catena.2010.10.009]

[3] Bruelheide H*, et al.* Community assembly during secondary forest succession in a Chinese subtropical forest. Ecological Monographs 2011; 81(1): 25–41
[https://doi.org/10.1890/09-2172.1]

[4] Bruelheide H*, et al.* Designing forest biodiversity experiments: general considerations illustrated by a new large experiment in subtropical C hina. Methods Ecol Evol 2014; 5(1): 74–89
[https://doi.org/10.1111/2041-210X.12126]

[5] Bradstreet RB. Determination of Nitro Nitrogen by Kjeldahl Method. Analytical chemistry 1954; 26(1): 235–6.

[6] Frostegård Å, Tunlid A, Bååth E. Microbial biomass measured as total lipid phosphate in soils of different organic content. Journal of Microbiological Methods 1991; 14(3): 151–63
[https://doi.org/10.1016/0167-7012(91)90018-L]

[7] Ruess L, Chamberlain PM. The fat that matters: Soil food web analysis using fatty acids and their carbon stable isotope signature. Soil Biology and Biochemistry 2010; 42(11): 1898–910
[https://doi.org/10.1016/j.soilbio.2010.07.020]

[8] Scheu S. Automated measurement of the respiratory response of soil microcompartments: Active microbial biomass in earthworm faeces. Soil Biology and Biochemistry 1992; 24(11): 1113–8
[https://doi.org/10.1016/0038-0717(92)90061-2]

[9] Schöps R*, et al.* Land-Use Intensity Rather Than Plant Functional Identity Shapes Bacterial and Fungal Rhizosphere Communities. Front Microbiol 2018; 9: 2711
[https://doi.org/10.3389/fmicb.2018.02711][PMID: 30515138]

[10] Nawaz A*, et al.* DNA- and RNA- Derived Fungal Communities in Subsurface Aquifers Only Partly Overlap but React Similarly to Environmental Factors. Microorganisms 2019; 7(9)
[https://doi.org/10.3390/microorganisms7090341][PMID: 31514383]

[11] Caporaso JG*, et al.* Global patterns of 16S rRNA diversity at a depth of millions of sequences per sample. Proc Natl Acad Sci U S A 2011; 108 Suppl 1: 4516–22
[https://doi.org/10.1073/pnas.1000080107][PMID: 20534432]

[12] Gardes M, Bruns TD. ITS primers with enhanced specificity for basidiomycetes--application to the identification of mycorrhizae and rusts. Mol Ecol 1993; 2(2): 113–8
[https://doi.org/10.1111/j.1365-294x.1993.tb00005.x][PMID: 8180733]

[13] White TJ, Bruns T, Lee SJ, Taylor J. Amplification and direct sequencing of fungal ribosomal RNA genes for phylogenetics. PCR protocols: a guide to methods and applications. 1990; 18(1): 315–22.

[14] Ihrmark K*, et al.* New primers to amplify the fungal ITS2 region--evaluation by 454-sequencing of artificial and natural communities. FEMS Microbiol Ecol 2012; 82(3): 666–77
[https://doi.org/10.1111/j.1574-6941.2012.01437.x][PMID: 22738186]

[15] Bolyen E*, et al.* Reproducible, interactive, scalable and extensible microbiome data science using QIIME 2. Nat Biotechnol 2019; 37(8): 852–7
[https://doi.org/10.1038/s41587-019-0209-9][PMID: 31341288]

[16] Martin M. Cutadapt removes adapter sequences from high-throughput sequencing reads. EMBnet j. 2011; 17(1): 10
[https://doi.org/10.14806/ej.17.1.200]

[17] Callahan BJ*, et al.* DADA2: High-resolution sample inference from Illumina amplicon data. Nat Methods 2016; 13(7): 581–3
[https://doi.org/10.1038/nmeth.3869][PMID: 27214047]

[18] McMurdie PJ, Holmes S. phyloseq: an R package for reproducible interactive analysis and graphics of microbiome census data. PLoS ONE 2013; 8(4): e61217
[https://doi.org/10.1371/journal.pone.0061217][PMID: 23630581]

[19] Lahti L, Shetty S, Blake T, Salojarvi J. Microbiome R package. Tools Microbiome Anal R 2017.

[20] Liang Y, Liu X, Singletary MA, Wang K, Mattes TE. Relationships between the Abundance and Expression of Functional Genes from Vinyl Chloride (VC)-Degrading Bacteria and Geochemical Parameters at VC-Contaminated Sites. Environ Sci Technol 2017; 51(21): 12164–74
[https://doi.org/10.1021/acs.est.7b03521][PMID: 28981261]

[21] Zheng B, Zhu Y, Sardans J, Peñuelas J, Su J. QMEC: a tool for high-throughput quantitative assessment of microbial functional potential in C, N, P, and S biogeochemical cycling. Sci China Life Sci 2018; 61(12): 1451–62
[https://doi.org/10.1007/s11427-018-9364-7][PMID: 30136056]

[22] Looft T*, et al.* In-feed antibiotic effects on the swine intestinal microbiome. Proc Natl Acad Sci U S A 2012; 109(5): 1691–6
[https://doi.org/10.1073/pnas.1120238109][PMID: 22307632]

[23] Campbell CD, Chapman SJ, Cameron CM, Davidson MS, Potts JM. A rapid microtiter plate method to measure carbon dioxide evolved from carbon substrate amendments so as to determine the physiological profiles of soil microbial communities by using whole soil. Appl Environ Microbiol 2003; 69(6): 3593–9
[https://doi.org/10.1128/aem.69.6.3593-3599.2003][PMID: 12788767]

[24] Rosseel Y. Lavaan: An R package for structural equation modeling and more. Version 0.5–12 (BETA). Journal of statistical software 2012; 48(2): 1–36.
