# Supplementary material for: Tree diversity and soil chemical properties drive the linkages between soil microbial community and ecosystem functioning
Source: ISME Commun. 2021 Aug 23;1:41. doi: 10.1038/s43705-021-00040-0 (PMC9723754; doi:10.1038/s43705-021-00040-0)
Supplement: Supplementary file 2 — supplemental-data S2 [file 43705_2021_40_MOESM2_ESM.pdf]

Supplementary material S2

List of tree species building the pairs of tree species in the different plots of Site A (BEF China experiment)

Species	Leaf persistence
Castanea henryi	deciduous
Castanopsis sclerophylla	evergreen
Choerospondias axillaris	deciduous
Cyclobalanopsis glauca	evergreen
Koelreuteria bipinnata	deciduous
Liquidambar formosana	deciduous
Lithocarpus glaber	evergreen
Nyssa sinensis	deciduous
Quercus fabri	deciduous
Quercus serrata	deciduous
Sapindus mukorossi	deciduous
Sapium sebiferum	deciduous

Sampling point description and attributes (paragraphs were added for readability)

Code	Site	Plot	Diversity level	Species 1	Species 2
26-E24	A	E24	1	*Liquidambar formosana*	*Liquidambar formosana*
33-E31	A	E31	1	*Quercus fabri*	*Quercus fabri*
34-E31	A	E31	1	*Quercus fabri*	*Quercus fabri*
27-E33	A	E33	1	*Lithocarpus glaber*	*Lithocarpus glaber*
28-E33	A	E33	1	*Lithocarpus glaber*	*Lithocarpus glaber*
1-E34	A	E34	1	*Castanea henryi*	*Castanea henryi*
2-E34	A	E34	1	*Castanea henryi*	*Castanea henryi*
37-F21	A	F21	1	*Quercus serrata*	*Quercus serrata*
38-F21	A	F21	1	*Quercus serrata*	*Quercus serrata*
10-G17	A	G17	1	*Castanopsis sclerophylla*	*Castanopsis sclerophylla*
29-G22	A	G22	1	*Lithocarpus glaber*	*Lithocarpus glaber*
22-G24	A	G24	1	*Koelreuteria bipinnata*	*Koelreuteria bipinnata*
23-G24	A	G24	1	*Koelreuteria bipinnata*	*Koelreuteria bipinnata*
36-G33	A	G33	1	*Quercus serrata*	*Quercus serrata*
30-H25	A	H25	1	*Nyssa sinensis*	*Nyssa sinensis*
3-I12	A	I12	1	*Castanea henryi*	*Castanea henryi*
24-I28	A	I28	1	*Liquidambar formosana*	*Liquidambar formosana*
25-I28	A	I28	1	*Liquidambar formosana*	*Liquidambar formosana*
14-K9	A	K9	1	*Cyclobalanopsis glauca*	*Cyclobalanopsis glauca*
8-L11	A	L11	1	*Castanopsis sclerophylla*	*Castanopsis sclerophylla*
9-L11	A	L11	1	*Castanopsis sclerophylla*	*Castanopsis sclerophylla*
13-L23	A	L23	1	*Choerospondias axillaris*	*Choerospondias axillaris*
43-N11	A	N11	1	*Sapindus mukorossi*	*Sapindus mukorossi*
46-N13	A	N13	1	*Sapium sebiferum*	*Sapium sebiferum*

(continued)

Code	Site	Plot	Diversity level	Species 1	Species 2
47-N13	A	N13	1	*Sapium sebiferum*	*Sapium sebiferum*
11-O27	A	O27	1	*Choerospondias axillaris*	*Choerospondias axillaris*
21-Q13	A	Q13	1	*Koelreuteria bipinnata*	*Koelreuteria bipinnata*
r-21-Q13	A	Q13	1	*Koelreuteria bipinnata*	*Koelreuteria bipinnata*
35-Q16	A	Q16	1	*Quercus fabri*	*Quercus fabri*
15-R14	A	R14	1	*Cyclobalanopsis glauca*	*Cyclobalanopsis glauca*
16-R14	A	R14	1	*Cyclobalanopsis glauca*	*Cyclobalanopsis glauca*
44-R17	A	R17	1	*Sapindus mukorossi*	*Sapindus mukorossi*
45-W13	A	W13	1	*Sapium sebiferum*	*Sapium sebiferum*
32-W14	A	W14	1	*Nyssa sinensis*	*Nyssa sinensis*
51-C32	A	C32	2	*Castanea henryi*	*Castanea henryi*
52-C32	A	C32	2	*Castanea henryi*	*Nyssa sinensis*
96-C32	A	C32	2	*Castanea henryi*	*Nyssa sinensis*
95-C32	A	C32	2	*Nyssa sinensis*	*Nyssa sinensis*
97-C32	A	C32	2	*Nyssa sinensis*	*Nyssa sinensis*
53-F22	A	F22	2	*Castanea henryi*	*Castanea henryi*
54-F22	A	F22	2	*Castanea henryi*	*Castanea henryi*
55-F22	A	F22	2	*Castanea henryi*	*Nyssa sinensis*
98-F22	A	F22	2	*Nyssa sinensis*	*Nyssa sinensis*
87-H31	A	H31	2	*Liquidambar formosana*	*Liquidambar formosana*
86-H31	A	H31	2	*Liquidambar formosana*	*Sapindus mukorossi*
113-H31	A	H31	2	*Sapindus mukorossi*	*Liquidambar formosana*
112-H31	A	H31	2	*Sapindus mukorossi*	*Sapindus mukorossi*
118-I27	A	I27	2	*Sapium sebiferum*	*Sapium sebiferum*
81-J21	A	J21	2	*Koelreuteria bipinnata*	*Koelreuteria bipinnata*
82-J21	A	J21	2	*Koelreuteria bipinnata*	*Koelreuteria bipinnata*
83-J21	A	J21	2	*Koelreuteria bipinnata*	*Lithocarpus glaber*
92-J21	A	J21	2	*Lithocarpus glaber*	*Lithocarpus glaber*
72-K3	A	K3	2	*Cyclobalanopsis glauca*	*Cyclobalanopsis glauca*
73-K3	A	K3	2	*Cyclobalanopsis glauca*	*Cyclobalanopsis glauca*
75-K3	A	K3	2	*Cyclobalanopsis glauca*	*Quercus fabri*
64-O6	A	O6	2	*Castanopsis sclerophylla*	*Castanopsis sclerophylla*
65-O6	A	O6	2	*Castanopsis sclerophylla*	*Castanopsis sclerophylla*
66-O6	A	O6	2	*Castanopsis sclerophylla*	*Quercus serrata*
105-O6	A	O6	2	*Quercus serrata*	*Quercus serrata*
63-P26	A	P26	2	*Castanopsis sclerophylla*	*Castanopsis sclerophylla*
62-P26	A	P26	2	*Castanopsis sclerophylla*	*Quercus serrata*
102-P26	A	P26	2	*Quercus serrata*	*Quercus serrata*
103-P26	A	P26	2	*Quercus serrata*	*Quercus serrata*
104-P26	A	P26	2	*Quercus serrata*	*Quercus serrata*
74-Q21	A	Q21	2	*Cyclobalanopsis glauca*	*Cyclobalanopsis glauca*
76-Q21	A	Q21	2	*Cyclobalanopsis glauca*	*Quercus fabri*
77-Q21	A	Q21	2	*Cyclobalanopsis glauca*	*Quercus fabri*
100-Q21	A	Q21	2	*Quercus fabri*	*Quercus fabri*
101-Q21	A	Q21	2	*Quercus fabri*	*Quercus fabri*
84-Q7	A	Q7	2	*Koelreuteria bipinnata*	*Koelreuteria bipinnata*
85-Q7	A	Q7	2	*Koelreuteria bipinnata*	*Lithocarpus glaber*

(continued)

Code	Site	Plot	Diversity level	Species 1	Species 2
93-Q7	A	Q7	2	*Lithocarpus glaber*	*Lithocarpus glaber*
94-Q7	A	Q7	2	*Lithocarpus glaber*	*Lithocarpus glaber*
69-S18	A	S18	2	*Choerospondias axillaris*	*Choerospondias axillaris*
70-S18	A	S18	2	*Choerospondias axillaris*	*Sapium sebiferum*
71-S18	A	S18	2	*Choerospondias axillaris*	*Sapium sebiferum*
119-S18	A	S18	2	*Sapium sebiferum*	*Sapium sebiferum*
r-120-S18	A	S18	2	*Sapium sebiferum*	*Sapium sebiferum*
88-T17	A	T17	2	*Liquidambar formosana*	*Liquidambar formosana*
89-T17	A	T17	2	*Liquidambar formosana*	*Liquidambar formosana*
90-T17	A	T17	2	*Liquidambar formosana*	*Sapindus mukorossi*
115-T17	A	T17	2	*Sapindus mukorossi*	*Sapindus mukorossi*
130-F27	A	F27	4	*Castanopsis sclerophylla*	*Castanopsis sclerophylla*
131-F27	A	F27	4	*Choerospondias axillaris*	*Castanopsis sclerophylla*
153-F27	A	F27	4	*Quercus serrata*	*Choerospondias axillaris*
161-F27	A	F27	4	*Sapium sebiferum*	*Choerospondias axillaris*
162-F27	A	F27	4	*Sapium sebiferum*	*Sapium sebiferum*
139-F28	A	F28	4	*Koelreuteria bipinnata*	*Koelreuteria bipinnata*
132-N20	A	N20	4	*Choerospondias axillaris*	*Choerospondias axillaris*
154-N20	A	N20	4	*Quercus serrata*	*Castanopsis sclerophylla*
155-N20	A	N20	4	*Quercus serrata*	*Quercus serrata*
156-N20	A	N20	4	*Quercus serrata*	*Sapium sebiferum*
163-N20	A	N20	4	*Sapium sebiferum*	*Castanopsis sclerophylla*
133-N8	A	N8	4	*Cyclobalanopsis glauca*	*Cyclobalanopsis glauca*
149-N8	A	N8	4	*Quercus fabri*	*Cyclobalanopsis glauca*
125-P19	A	P19	4	*Castanea henryi*	*Castanea henryi*
126-P19	A	P19	4	*Castanea henryi*	*Nyssa sinensis*
143-P19	A	P19	4	*Liquidambar formosana*	*Sapindus mukorossi*
148-P19	A	P19	4	*Nyssa sinensis*	*Sapindus mukorossi*
160-P19	A	P19	4	*Sapindus mukorossi*	*Sapindus mukorossi*
141-P29	A	P29	4	*Liquidambar formosana*	*Liquidambar formosana*
142-P29	A	P29	4	*Liquidambar formosana*	*Nyssa sinensis*
147-P29	A	P29	4	*Nyssa sinensis*	*Castanea henryi*
159-P29	A	P29	4	*Sapindus mukorossi*	*Castanea henryi*
146-W12/X12	A	W12/X12	4	*Lithocarpus glaber*	*Lithocarpus glaber*
176-P27	A	P27	8	*Cyclobalanopsis glauca*	*Quercus fabri*
181-P27	A	P27	8	*Koelreuteria bipinnata*	*Lithocarpus glaber*
166-R16	A	R16	8	*Castanea henryi*	*Liquidambar formosana*
171-R16	A	R16	8	*Castanopsis sclerophylla*	*Castanopsis sclerophylla*
175-R16	A	R16	8	*Choerospondias axillaris*	*Sapium sebiferum*
190-R16	A	R16	8	*Nyssa sinensis*	*Castanea henryi*
193-R16	A	R16	8	*Quercus serrata*	*Castanopsis sclerophylla*
194-R16	A	R16	8	*Quercus serrata*	*Quercus serrata*
198-R16	A	R16	8	*Sapindus mukorossi*	*Sapindus mukorossi*
199-R16	A	R16	8	*Sapindus mukorossi*	*Sapindus mukorossi*
200-R16	A	R16	8	*Sapium sebiferum*	*Quercus serrata*
201-R16	A	R16	8	*Sapium sebiferum*	*Sapium sebiferum*
165-S10	A	S10	8	*Castanea henryi*	*Castanea henryi*
170-S10	A	S10	8	*Castanopsis sclerophylla*	*Sapium sebiferum*

(continued)

Code	Site	Plot	Diversity level	Species 1	Species 2
173-S10	A	S10	8	*Choerospondias axillaris*	*Castanopsis sclerophylla*
174-S10	A	S10	8	*Choerospondias axillaris*	*Choerospondias axillaris*
186-S10	A	S10	8	*Liquidambar formosana*	*Liquidambar formosana*
185-S10	A	S10	8	*Liquidambar formosana*	*Nyssa sinensis*
188-S10	A	S10	8	*Nyssa sinensis*	*Nyssa sinensis*
189-S10	A	S10	8	*Nyssa sinensis*	*Sapindus mukorossi*
197-S10	A	S10	8	*Sapindus mukorossi*	*Castanea henryi*
178-S14	A	S14	8	*Cyclobalanopsis glauca*	*Cyclobalanopsis glauca*
183-S15	A	S15	8	*Koelreuteria bipinnata*	*Koelreuteria bipinnata*
r-216-S15	A	S15	8	*Koelreuteria bipinnata*	*Lithocarpus glaber*
184-S15	A	S15	8	*Koelreuteria bipinnata*	*Quercus fabri*
191-T15	A	T15	8	*Quercus fabri*	*Quercus fabri*
220-L21	A	L21	16	*Liquidambar formosana*	*Choerospondias axillaris*
203-L22	A	L22	16	*Castanea henryi*	*Nyssa sinensis*
204-L22	A	L22	16	*Castanea henryi*	*Sapindus mukorossi*
217-L22	A	L22	16	*Liquidambar formosana*	*Castanea henryi*
219-L22	A	L22	16	*Liquidambar formosana*	*Liquidambar formosana*
218-L22	A	L22	16	*Liquidambar formosana*	*Nyssa sinensis*
221-L22	A	L22	16	*Lithocarpus glaber*	*Lithocarpus glaber*
222-L22	A	L22	16	*Quercus fabri*	*Quercus fabri*
230-L22	A	L22	16	*Sapium sebiferum*	*Castanopsis sclerophylla*
226-M21	A	M21	16	*Quercus serrata*	*Sapium sebiferum*
r-213-U10	A	U10	16	*Cyclobalanopsis glauca*	*Quercus fabri*
225-U10	A	U10	16	*Quercus serrata*	*Quercus serrata*
229-U10	A	U10	16	*Sapindus mukorossi*	*Sapindus mukorossi*
231-U10	A	U10	16	*Sapium sebiferum*	*Sapium sebiferum*
232-N9	A	N9	24	*Castanea henryi*	*Castanea henryi*
236-N9	A	N9	24	*Cyclobalanopsis glauca*	*Cyclobalanopsis glauca*
238-N9	A	N9	24	*Koelreuteria bipinnata*	*Koelreuteria bipinnata*
241-N9	A	N9	24	*Sapindus mukorossi*	*Nyssa sinensis*
234-R18	A	R18	24	*Castanopsis sclerophylla*	*Quercus serrata*
235-R18	A	R18	24	*Choerospondias axillaris*	*Quercus serrata*
239-R18	A	R18	24	*Nyssa sinensis*	*Nyssa sinensis*
