# Supplementary material for: Tree diversity and soil chemical properties drive the linkages between soil microbial community and ecosystem functioning
Source: ISME Commun. 2021 Aug 23;1:41. doi: 10.1038/s43705-021-00040-0 (PMC9723754; doi:10.1038/s43705-021-00040-0)
Supplement: Supplementary file 3 — supplemental-data S3 [file 43705_2021_40_MOESM3_ESM.docx]

Supplementary material S3

PLFA biomarkers used to identify soil microbes’ functional groups

| Fatty acid | Lipid fraction | Predominant origin | Literature |
| --- | --- | --- | --- |
| i15:0 | PLFA | Gram-positive bacteria | [1-2] |
| a15:0 | PLFA | Gram-positive bacteria | [1-2] |
| i16:0 | PLFA | Gram-positive bacteria | [1-2] |
| i17:0 | PLFA | Gram-positive bacteria | [1-2] |
| 16:1n7 | PLFA | Bacteria widespread | [2-3] |
| 16:1n-5 | PLFA | General bacteria | [1-4] |
| cy17:0 | PLFA | Gram-negative bacteria | [1-2] |
| 18:1n9 | PLFA | Fungi (saprophytic, EM) | [2,5-8] |
| cy19:0 | PLFA | Gram-negative bacteria | [1-2] |
| 18:2n6c | PLFA | Fungi (saprophytic, EM) | [2,9] |
| 20:1 | PLFA | AM fungi (Gigaspora) | [10] |

**Cited literature**

[1] Zelles, L., *et al.* (1997). Changes in soil microbial properties and phospholipid fatty acid fractions after chloroform fumigation. Soil Biology and Biochemistry, 29(9-10), 1325-1336.

[2] Zelles, L. (1999). Fatty acid patterns of phospholipids and lipopolysaccharides in the characterisation of

microbial communities in soil: a review. Biology and fertility of soils, 29(2), 111-129.

[3] Guckert, J. B., Ringelberg, D. B., White, D. C., Hanson, R. S., & Bratina, B. J. (1991). Membrane fatty acids as phenotypic markers in the polyphasic taxonomy of methylotrophs within the Proteobacteria. Microbiology, 137(11), 2631-2641.

[4] Nichols, P. D., *et al.* (1987). Detection of a microbial consortium, including type II methanotrophs, by use of phospholipid fatty acids in an aerobic halogenated hydrocarbon-degrading soil column enriched with natural gas. Environmental Toxicology and Chemistry: An International Journal, 6(2), 89-97.

[5] Baath, E., & Anderson, T. H. (2003). Comparison of soil fungal/bacterial ratios in a pH gradient using physiological and PLFA-based techniques. Soil Biology and Biochemistry, 35(7), 955-963.

[6] Vestal, J. R., & White, D. C. (1989). Lipid analysis in microbial ecology. Bioscience, 39(8), 535-541.1

[7] Harwood, J. L., & Russell, N. J. (1984). Distribution of lipids. In Lipids in plants and microbes (pp. 35-70). Springer, Dordrecht.

[8] Ruess, L., & Chamberlain, P. M. (2010). The fat that matters: soil food web analysis using fatty acids and their carbon stable isotope signature. Soil Biology and Biochemistry, 42(11), 1898-1910.

[9] Frostegard, A., & Baath, E. (1996). The use of phospholipid fatty acid analysis to estimate bacterial and fungal biomass in soil. Biology and Fertility of soils, 22(1-2), 59-65.

[10] Sakamoto, K., Iijima, T., & Higuchi, R. (2004). Use of specific phospholipid fatty acids for identifying and quantifying the external hyphae of the arbuscular mycorrhizal fungus Gigaspora rosea. Soil Biology and Biochemistry, 36(11), 1827-1834.
