# Supplementary material for: Tree diversity and soil chemical properties drive the linkages between soil microbial community and ecosystem functioning
Source: ISME Commun. 2021 Aug 23;1:41. doi: 10.1038/s43705-021-00040-0 (PMC9723754; doi:10.1038/s43705-021-00040-0)
Supplement: Supplementary file 4 — supplemental-data S4 [file 43705_2021_40_MOESM4_ESM.docx]

**Supplementary material S4**

1. **Correlations between the microbial variable**


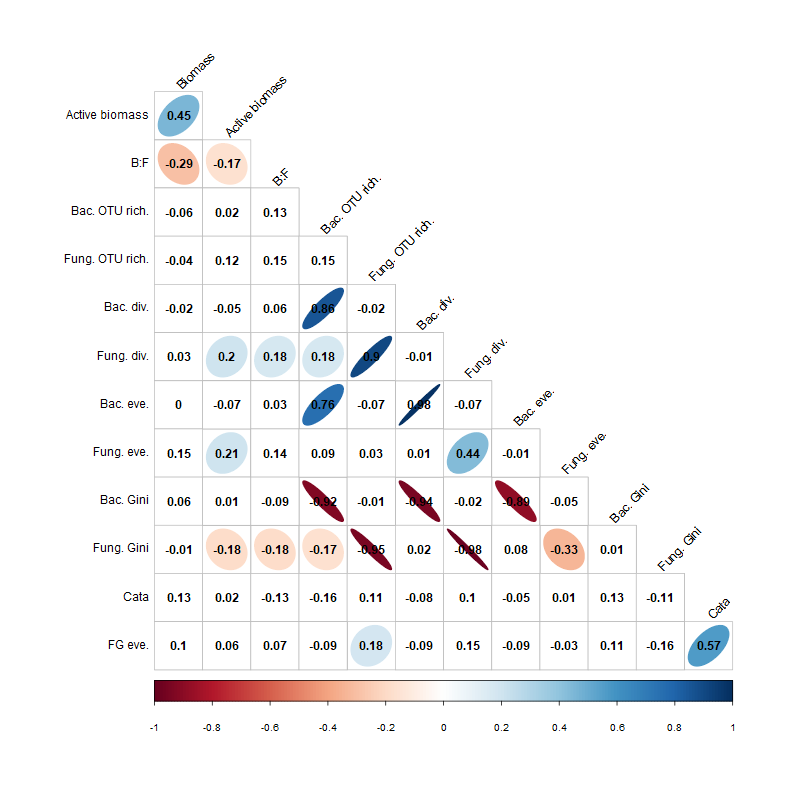


1. **The structured list of variables used in the analyses.**

| Variable group | Variable name (acronyms) | | Definition and measurement method | Hypotheses and analyses referring to it |
| --- | --- | --- | --- | --- |
| Soil chemical properties | Soil carbon content (TOC) | | Measured on top soil (0-10 cm) using TOC analyzer | Hypothesis 4, Fig. 5, Suppl. S10 |
|  | Soil carbon to nitrogen ratio (C:N) | | Calculated on top soil (0-10 cm) measurements | Hypothesis 4, Fig. 5, Suppl. S10 |
|  | Soil carbon to nitrogen ratio (C:P) | | Calculated on top soil (0-10 cm) measurements | Hypothesis 4, Fig. 5, Suppl. S10 |
|  | Soil pH (pH) | | Measured on top soil (0-10 cm) using 1:2.5 soil - water solution | Hypothesis 4, Fig. 5, Suppl. S10 |
|  | Soil water content (RH) | | Measured on 25 g of top soil (0-10 cm) air-dryed at 40 °C | Hypothesis 4, Fig. 5, Suppl. S10 |
| Tree Species Richness | Tree species richness | | Number of tree species per plot | Hypothesis 1&4, Fig. 1&5, Suppl. S7&10 |
| Soil microbial community facets | Microbial biomass | Total microbial biomass (Biomass) | Total microbial biomass calculated from PLFA markers measurements | Hypotheses 1-4, Fig. 1-5, Suppl. S3, S7-10 |
|  |  | Active microbial biomass (Active biomass) | The active fraction of the total microbial biomass calculated from substrate-induced respiration (SIR, Scheu 1992) | Hypotheses 1-4, Fig. 1-5, Suppl. S3, S7-S10 |
|  | Taxonomic profile | Bacteria to fungi ratio (B:F) | Bacteria to fungi ratio was calculated using microbial functional groups biomass measured by PLFA analyses | Hypotheses 1-4, Fig. 1-5, Suppl. S3, S6-S10 |
|  |  | Bacteria Shannon diversity (Bac. div.) | Baterial community Shannon diversity calculated from 16S sequencing data | Hypotheses 1-4, Fig. 1-5, Suppl. S3, S6-S10 |
|  |  | Fungi Shannon diversity (Fung. div.) | Fungi community Shannon diversity calculated from ITS sequencing data | Hypotheses 1-4, Fig. 1-5, Suppl. S3, S6-10 |
|  | Functional profile | FG evenness | The absolute or relative abundance of functional genes measured by qPCR. See Suppl. S5 for a complete list of measured functional genes | Hypotheses 1-4, Fig. 1-4, Suppl. S5 & S7-9 |
|  |  | Catabolism functional genes (Cata) | Sum of the abundance of functional genes involved in carbon catabolism. The variables can be calculated on the absolute or relative abundance of the functional genes and will be specified. | Hypotheses 1-4, Fig. 1-4, Suppl. S5 & S7-9 |
| Soil physiological potential | Substrate induced-respiration (SIR) | | Substrate-induced respiration (i.e. CO_2_ production during six hours after substrate addition) of fourteen substrates (i.e. 5 saccharides, 4 amino-acids, and 5 carboxylic-acids) measured with the Microresp® method. | Hypotheses 1-4, Fig. 1-5, Suppl. S5 & S7-9 |
|  | Substrate-induced respiration efficiency (SIR efficiency) | | Pielou evenness of the substrate-induced respiration (i.e. CO_2_ production during six hours after substrate addition) of fourteen substrates (i.e. complete list in Suppl. S3) | Hypotheses 1-4, Fig. 1-5, Suppl. S5 & S7-9 |
|  | Substrate-induced respiration response range (SIR range) | | The absolute difference of CO_2_ production between alamine induced respiration and oxalic-acid induced respiration measured with the Microresp® method. | Hypotheses 1-4, Fig. 1-5, Suppl. S5 & S7-9 |
| Ecosystem function | Microbial respiration (M. resp.) | | Soil basal respiration measured (SIR, Scheu 1992) | Hypotheses 1-4, Fig. 1-5, Suppl. S7-9 |
