# Supplementary material for: Tree diversity and soil chemical properties drive the linkages between soil microbial community and ecosystem functioning
Source: ISME Commun. 2021 Aug 23;1:41. doi: 10.1038/s43705-021-00040-0 (PMC9723754; doi:10.1038/s43705-021-00040-0)
Supplement: Supplementary file 5 — supplemental-data S5 [file 43705_2021_40_MOESM5_ESM.docx]

**Supplementary material S4**

List of functional genes and their functional attributes

| Function in the carbon cycle | Functional gene name | Specific functional gene function |
| --- | --- | --- |
| Carbon catabolism | abfA | Hemicellulose |
|  | apu | Starch |
|  | cex | Cellulose |
|  | chiA | Chitin |
|  | ipu | Starch |
|  | lig | Lignin |
|  | manB | Hemicellulose |
|  | mnp | Lignin |
|  | mxaF | Methane production |
|  | naglu | Cellulose |
|  | pox | Lignin |
|  | pqq-mdh | Methane production |
|  | sga | Starch |
|  | xylA | Hemicellulose |
