# Supplementary material for: Tree diversity and soil chemical properties drive the linkages between soil microbial community and ecosystem functioning
Source: ISME Commun. 2021 Aug 23;1:41. doi: 10.1038/s43705-021-00040-0 (PMC9723754; doi:10.1038/s43705-021-00040-0)

**Supplementary material S5**

1. **List of substrates used in substrate-induced respiration measurements (i.e. Microresp® method) and chemical attributes.**

| Full name | Chemical group | Formula | Molecular Weight | Mean carbon oxidation state |
| --- | --- | --- | --- | --- |
| L-Alamine | Amino acid | C_3_H_7_NO_2_ | 89.094 | -2 |
| γ-Aminobutyric acid | Amino acid | C_4_H_9_NO_2_ | 103.121 | -2 |
| L-Cysteine-HCl | Amino acid | C_3_H_8_ClNO_2_S | 157.612 | -1.33 |
| L-Arginine | Amino acid | C_6_H_14_N_4_O_2_ | 174.204 | -1 |
| L-Lysine-HCl | Amino acid | C_6_H_15_ClN_2_O_2_ | 182.648 | -1 |
| Oxalic acid | Carboxylic acid | (COOH)_2_ | 90.034 | 3 |
| L-Malic acid | Carboxylic acid | C_4_H_6_O_5_ | 134.087 | 1 |
| α-Ketoglutaric acid | Carboxylic acid | C_5_H_6_O_5_ | 146.11 | 0.8 |
| Citric acid | Carboxylic acid | C_6_H_8_O_7_ | 192.123 | 1 |
| L-(+)-Arabinose | Sugar | C_5_H_10_O_5_ | 150.13 | 0 |
| D-(-)-Fructose | Sugar | C_6_H_12_O_6_ | 180.156 | 0 |
| D-(+)-Galactose | Sugar | C_6_H_12_O_6_ | 180.156 | 0 |
| D-(+)-Glucose | Sugar | C_6_H_12_O_6_ | 180.156 | 0 |
| N-Acetyl glucosamine | Sugar | C_8_H_15_NO_6_ | 221.209 | -1 |

1. **CO_2_ production during the six hours following the substrate addition in the Microresp. ® measurements.** CO_2_ production against substrate molecular weight (**A.**) or against mean carbon oxidation state (**B.**).


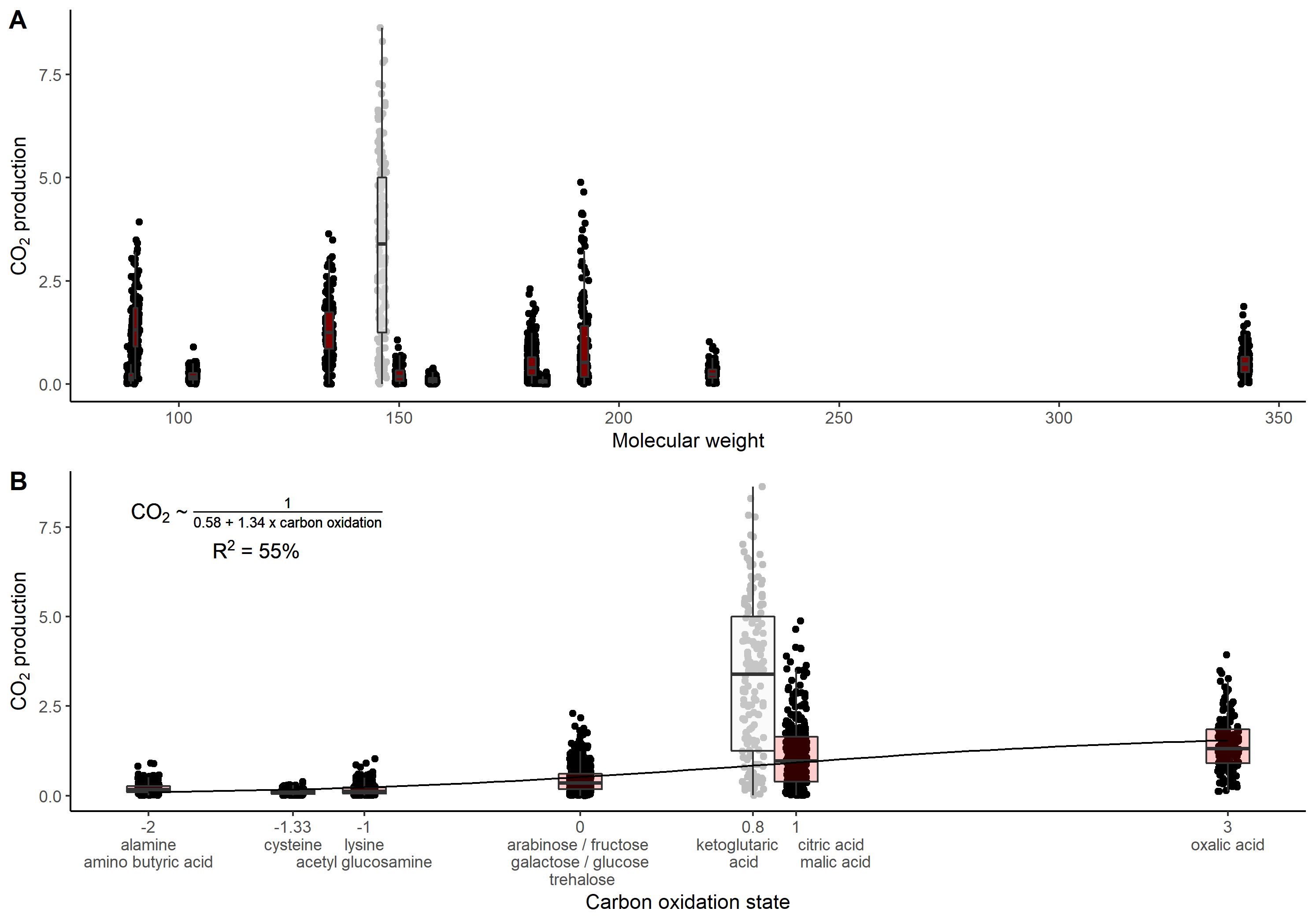


1. **Effect of (A.) substrate removal and (B.) change of induction range definition on indices values**


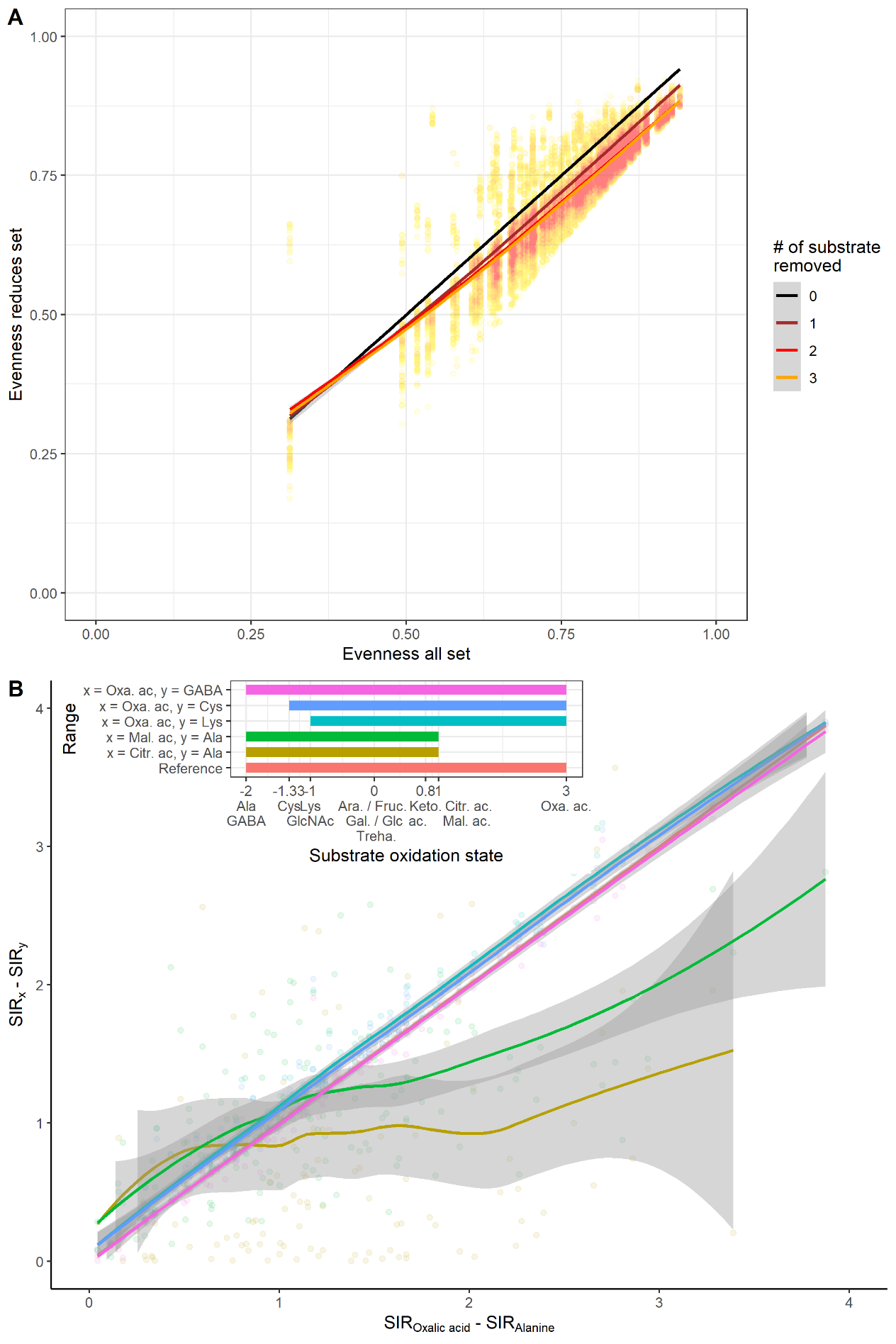

Supplement: Supplementary file 6 — supplemental-data S6 [file 43705_2021_40_MOESM6_ESM.docx]
