# Supplementary material for: Tree diversity and soil chemical properties drive the linkages between soil microbial community and ecosystem functioning
Source: ISME Commun. 2021 Aug 23;1:41. doi: 10.1038/s43705-021-00040-0 (PMC9723754; doi:10.1038/s43705-021-00040-0)
Supplement: Supplementary file 7 — supplemental-data S7 [file 43705_2021_40_MOESM7_ESM.pdf]

Supplementary material S7

Statistical analyses and R outputs related to the main figure Fig. 2

A. Model shape selection

Tree species richness effect on soil microbial facets and functions. For each soil microbial facets and functions, we tested the shape of the relationship using the 'lm' function and the following relations: linear (i.e. y ~ x), quadratic (i.e. y ~ x^2), polynomial (i.e. y ~ x + x^2 + x^3) and logarithmic (i.e. y ~ log(x)). The models were ordered by AIC and considered different when the difference of AIC was higher than 4. When several models had a comparable fit (difference of AIC below 4) the simplest model was chosen (i.e. linear < logarithmic < quadratic < polynomial).

Microbial biomass

Total microbial biomass

Name	Model	AIC	BIC	R2	R2_adjusted	RMSE	Sigma
polynomial	y ~ poly(x, degree = 3)	2886	2901	0.098	0.079	4013.930	4069.297
linear	y ~ x	2889	2898	0.056	0.050	4105.131	4133.153
quadratic	y ~ x^2	2889	2898	0.056	0.050	4105.131	4133.153
log	y ~ log(x)	2893	2902	0.027	0.021	4167.715	4196.163

Active microbial biomass

Name	Model	AIC	BIC	R2	R2_adjusted	RMSE	Sigma
polynomial	y ~ poly(x, degree = 3)	1768	1783	0.049	0.029	91.784	93.050
linear	y ~ x	1770	1779	0.010	0.003	93.621	94.260
quadratic	y ~ x^2	1770	1779	0.010	0.003	93.621	94.260
log	y ~ log(x)	1771	1780	0.001	-0.006	94.060	94.702

Microbial taxonomic profile

Bacteria to fungi ratio

Name	Model	AIC	BIC	R2	R2_adjusted	RMSE	Sigma
log	y ~ log(x)	207	216	0.016	0.010	0.477	0.480
linear	y ~ x	207	216	0.015	0.008	0.477	0.480
quadratic	y ~ x^2	207	216	0.015	0.008	0.477	0.480
polynomial	y ~ poly(x, degree = 3)	211	226	0.017	-0.004	0.476	0.483

Bacterial Shannon diversity

Name	Model	AIC	BIC	R2	R2_adjusted	RMSE	Sigma
log	y ~ log(x)	388	397	0.060	0.053	0.881	0.887
polynomial	y ~ poly(x, degree = 3)	391	406	0.069	0.050	0.876	0.888
linear	y ~ x	391	400	0.044	0.037	0.888	0.894
quadratic	y ~ x^2	391	400	0.044	0.037	0.888	0.894

Fungal Shannon diversity

Name	Model	AIC	BIC	R2	R2_adjusted	RMSE	Sigma
log	y ~ log(x)	-98	-89	0.000	-0.006	0.170	0.171
linear	y ~ x	-98	-89	0.000	-0.007	0.170	0.171
quadratic	y ~ x^2	-98	-89	0.000	-0.007	0.170	0.171
polynomial	y ~ poly(x, degree = 3)	-97	-82	0.014	-0.006	0.169	0.171

Microbial functional profile

Catabolism functional genes

Name	Model	AIC	BIC	R2	R2_adjusted	RMSE	Sigma
log	y ~ log(x)	566	575	0.010	0.003	1.604	1.615
linear	y ~ x	566	575	0.007	0.000	1.607	1.618
quadratic	y ~ x^2	566	575	0.007	0.000	1.607	1.618
polynomial	y ~ poly(x, degree = 3)	569	584	0.014	-0.007	1.601	1.624

Functional genes evenness

Name	Model	AIC	BIC	R2	R2_adjusted	RMSE	Sigma
log	y ~ log(x)	-455	-446	0.003	-0.003	0.051	0.051
linear	y ~ x	-455	-446	0.003	-0.004	0.051	0.051
quadratic	y ~ x^2	-455	-446	0.003	-0.004	0.051	0.051
polynomial	y ~ poly(x, degree = 3)	-452	-437	0.011	-0.010	0.051	0.051

Microbial physiological potential

Substrate-induced respiration efficiency

Name	Model	AIC	BIC	R2	R2_adjusted	RMSE	Sigma
linear	y ~ x	41	50	0.071	0.064	0.272	0.274
quadratic	y ~ x^2	41	50	0.071	0.064	0.272	0.274
log	y ~ log(x)	42	51	0.063	0.057	0.274	0.275
polynomial	y ~ poly(x, degree = 3)	45	60	0.072	0.053	0.272	0.276

Substrate-induced respiration response range

Name	Model	AIC	BIC	R2	R2_adjusted	RMSE	Sigma
log	y ~ log(x)	340	349	0.005	-0.002	0.749	0.754
linear	y ~ x	341	350	0.003	-0.003	0.750	0.755
quadratic	y ~ x^2	341	350	0.003	-0.003	0.750	0.755
polynomial	y ~ poly(x, degree = 3)	345	360	0.004	-0.017	0.749	0.760

Microbial respiration

Name	Model	AIC	BIC	R2	R2_adjusted	RMSE	Sigma
linear	y ~ x	149	157	0.023	0.017	0.392	0.394
quadratic	y ~ x^2	149	157	0.023	0.017	0.392	0.394
log	y ~ log(x)	150	159	0.015	0.009	0.393	0.396
polynomial	y ~ poly(x, degree = 3)	151	166	0.035	0.015	0.389	0.395

B. Model quality check

Tree species richness effect on soil microbial facets and functions. The relation was tested using the “lm” function in R. Model statistical assumptions were tested using the “check_model” function from the R package “performance”.

Microbial biomass

Total microbial biomass

.

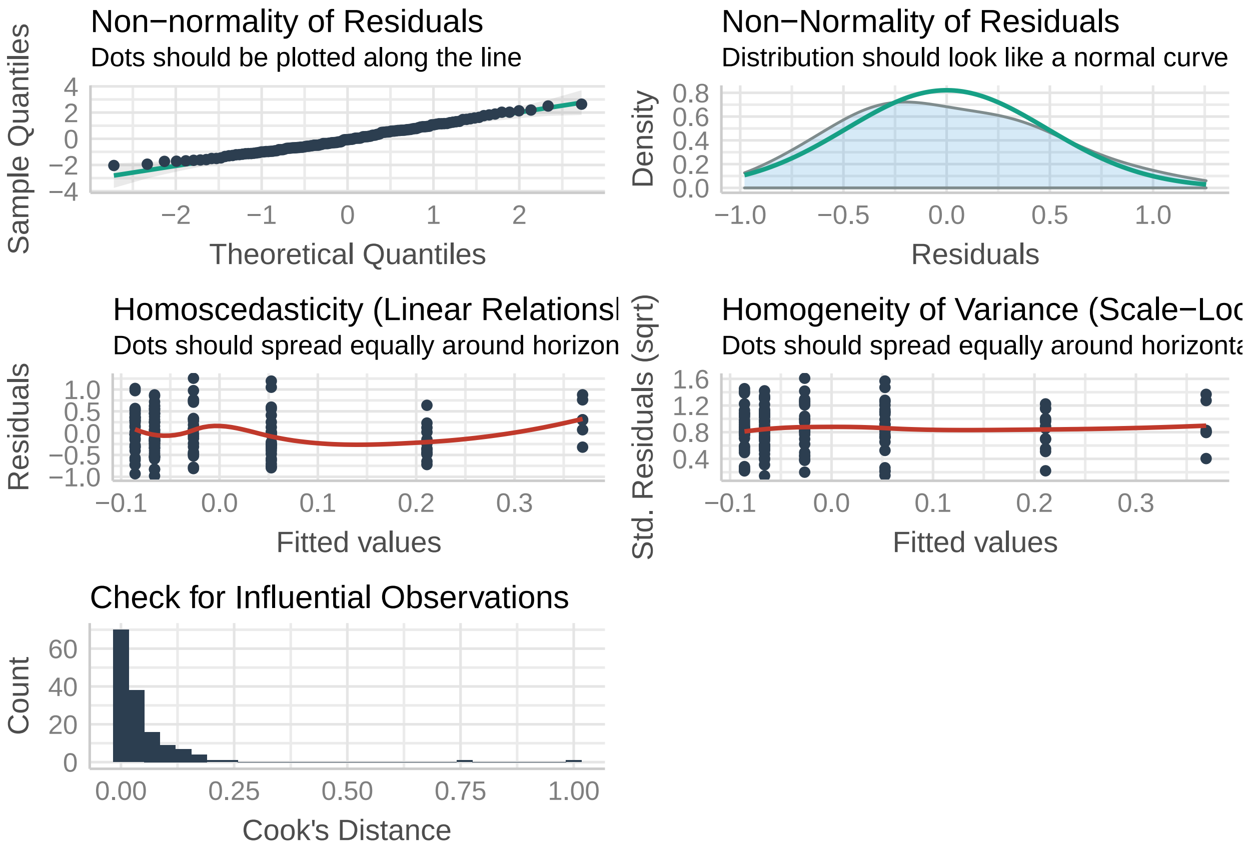

Explanatory	Estimate	SE	t.value	p.value
(Intercept)	-0.106	0.054	-1.96	0.051
Tree species richness	0.02	0.007	2.95	0.004

Active microbial biomass

.

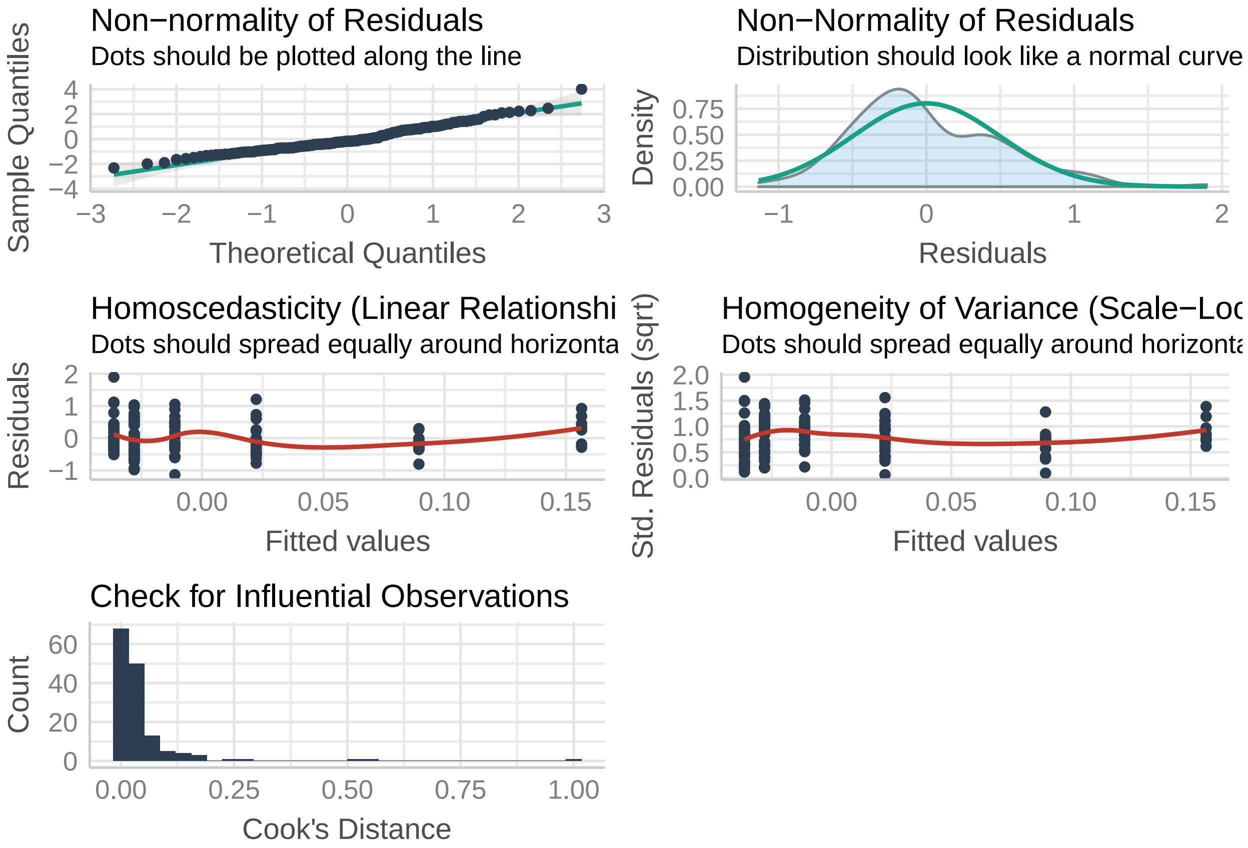

Explanatory	Estimate	SE	t.value	p.value
(Intercept)	-0.045	0.055	-0.81	0.417
Tree species richness	0.008	0.007	1.22	0.224

Microbial taxonomic profile

Bacteria to fungi ratio

.

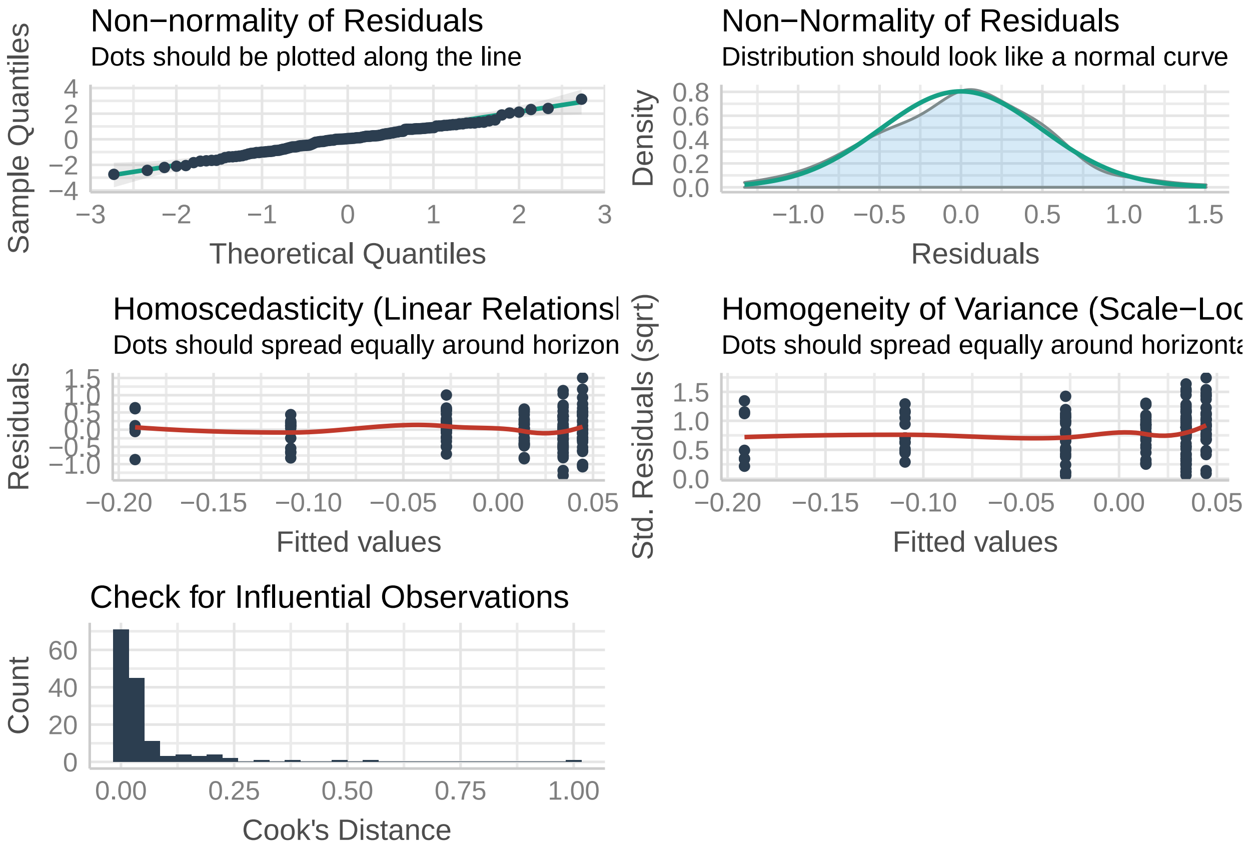

Explanatory	Estimate	SE	t.value	p.value
(Intercept)	0.055	0.055	1	0.32
Tree species richness	-0.01	0.007	-1.5	0.137

Bacterial Shannon diversity

.

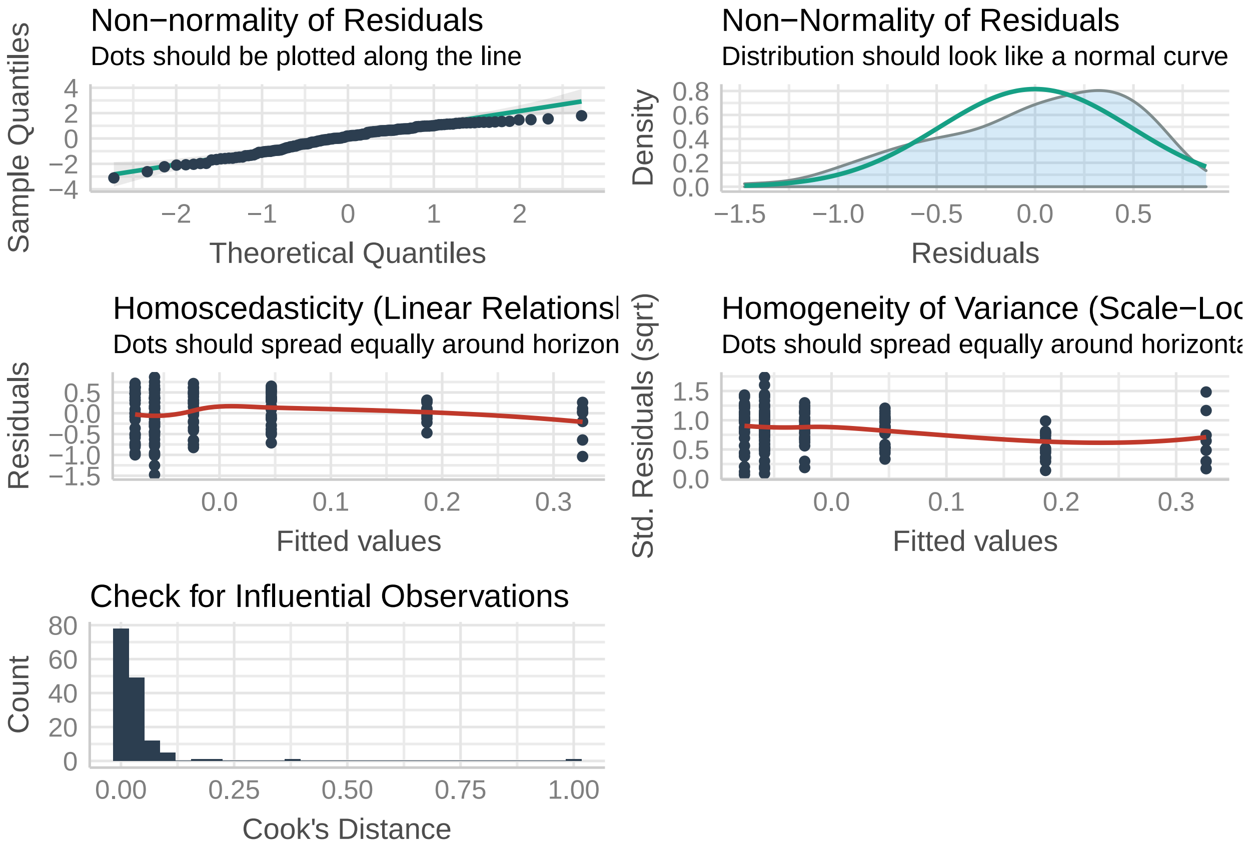

Explanatory	Estimate	SE	t.value	p.value
(Intercept)	-0.093	0.054	-1.72	0.087
Tree species richness	0.017	0.007	2.59	0.011

Fungal Shannon diversity

.

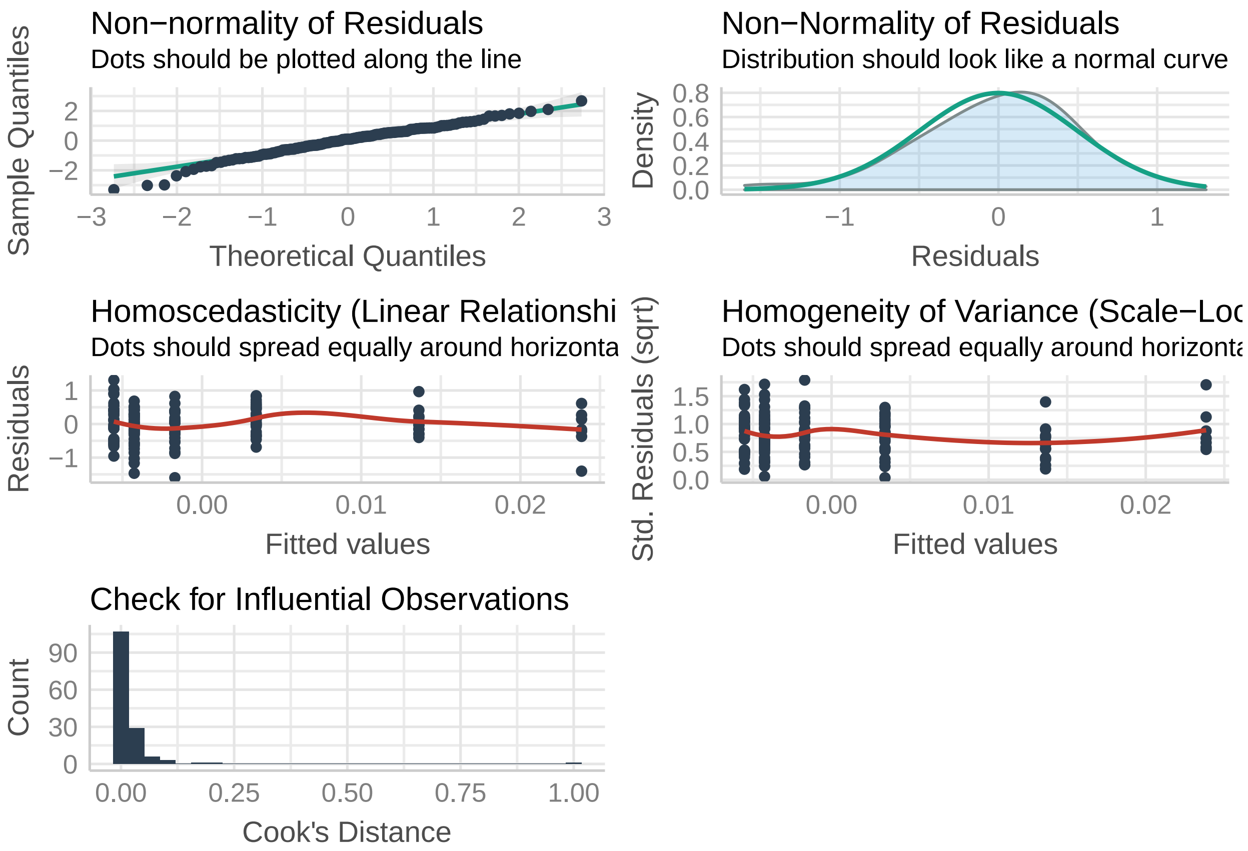

Explanatory	Estimate	SE	t.value	p.value
(Intercept)	-0.007	0.055	-0.12	0.902
Tree species richness	0.001	0.007	0.18	0.854

Microbial functional profile

Catabolism functional genes

.

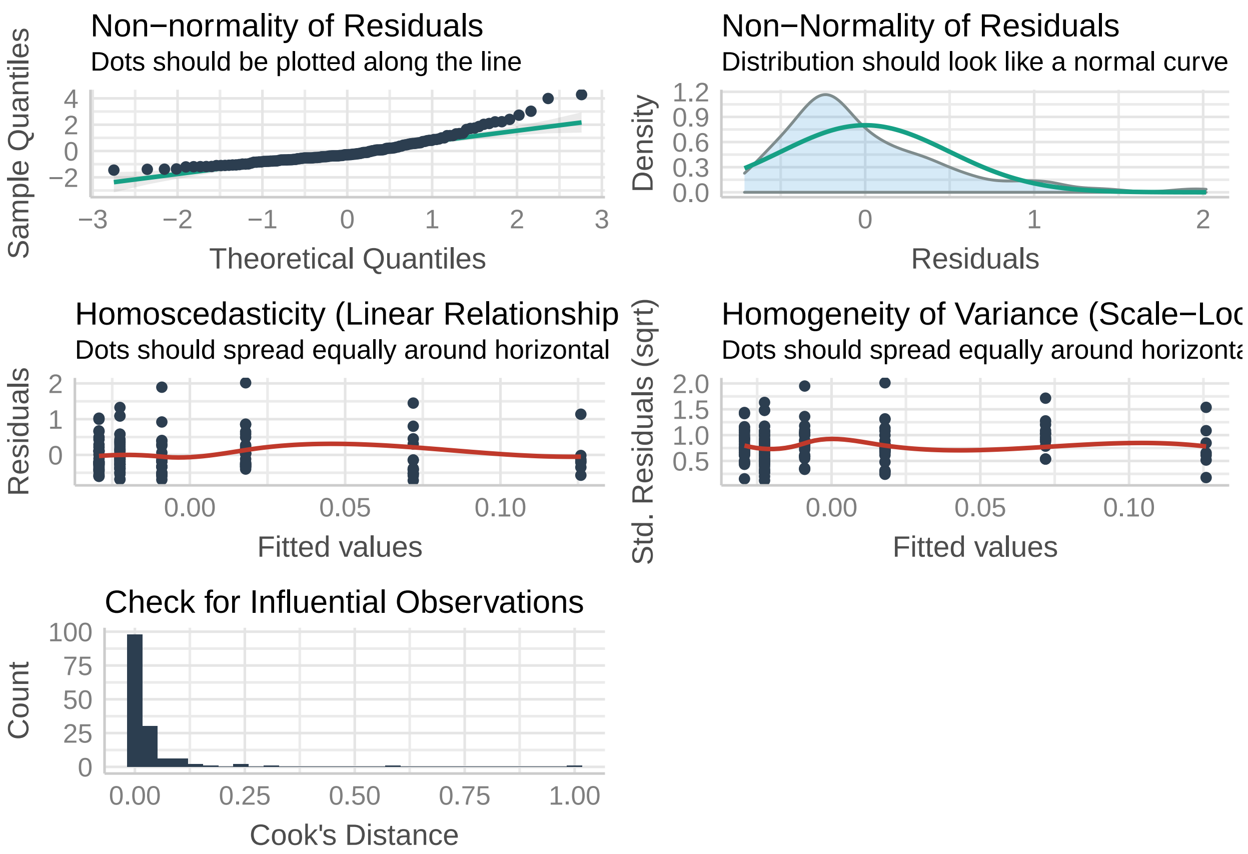

Explanatory	Estimate	SE	t.value	p.value
(Intercept)	-0.036	0.055	-0.65	0.515
Tree species richness	0.007	0.007	0.98	0.329

Functional genes evenness

.

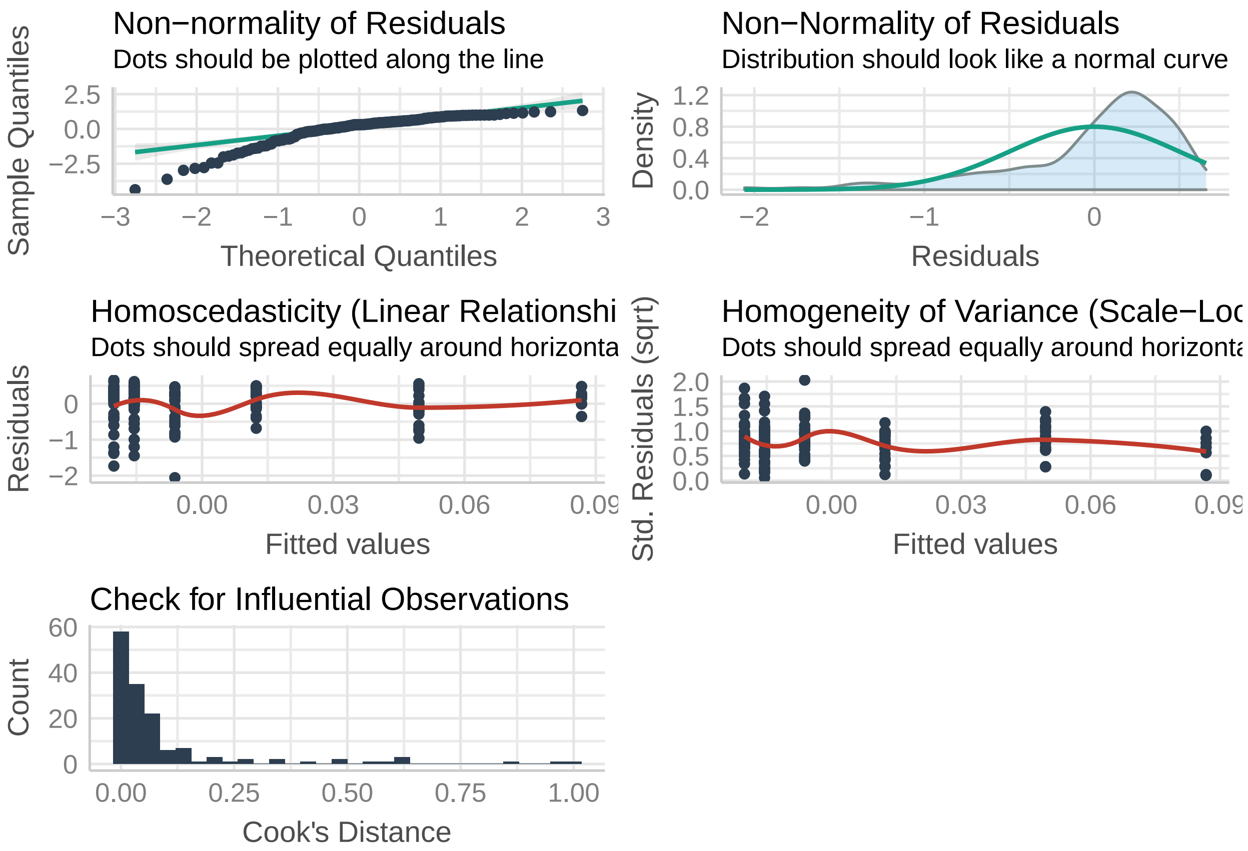

Explanatory	Estimate	SE	t.value	p.value
(Intercept)	-0.025	0.055	-0.45	0.654
Tree species richness	0.005	0.007	0.67	0.501

Microbial physiological potential

Substrate-induced respiration efficiency

.

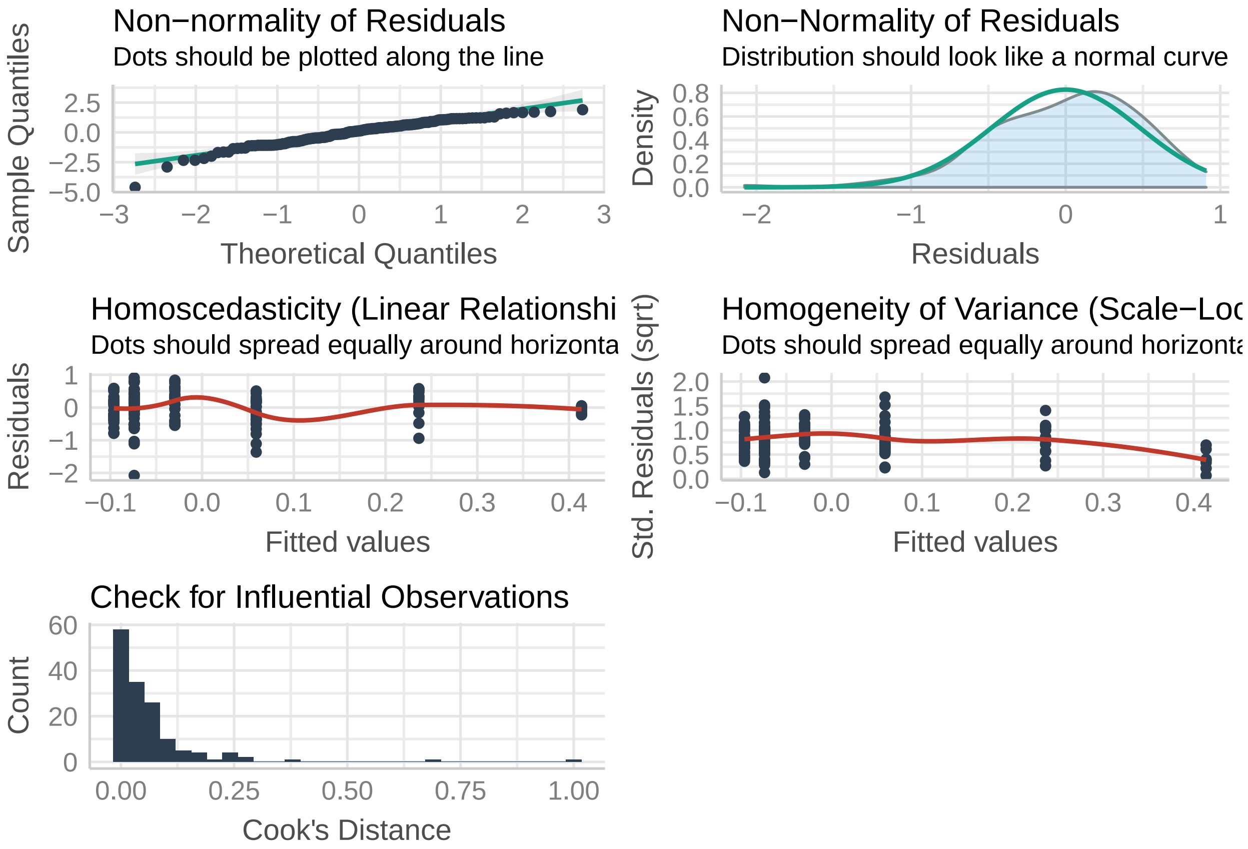

Explanatory	Estimate	SE	t.value	p.value
(Intercept)	-0.118	0.053	-2.22	0.028
Tree species richness	0.022	0.007	3.33	0.001

Substrate-induced respiration response range

.

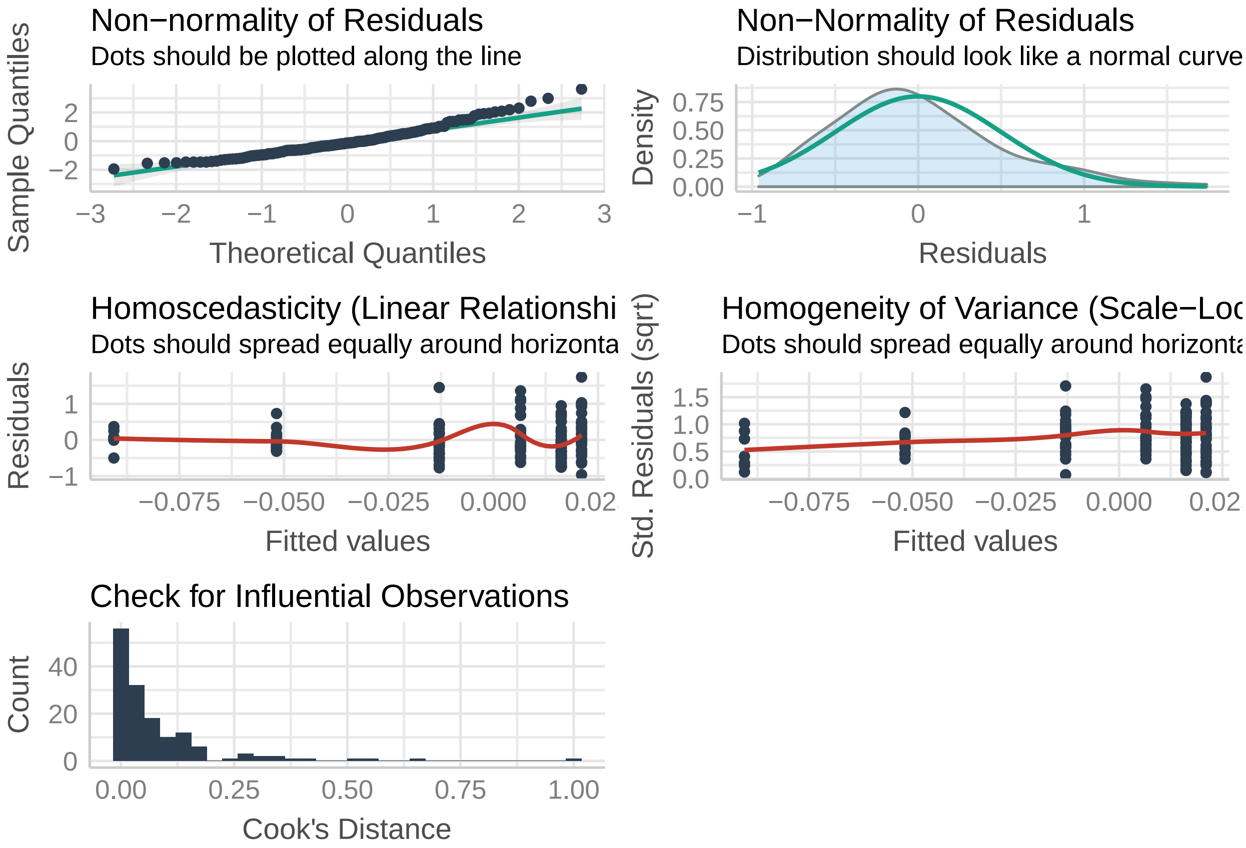

Explanatory	Estimate	SE	t.value	p.value
(Intercept)	0.026	0.055	0.47	0.639
Tree species richness	-0.005	0.007	-0.7	0.482

Microbial respiration

.

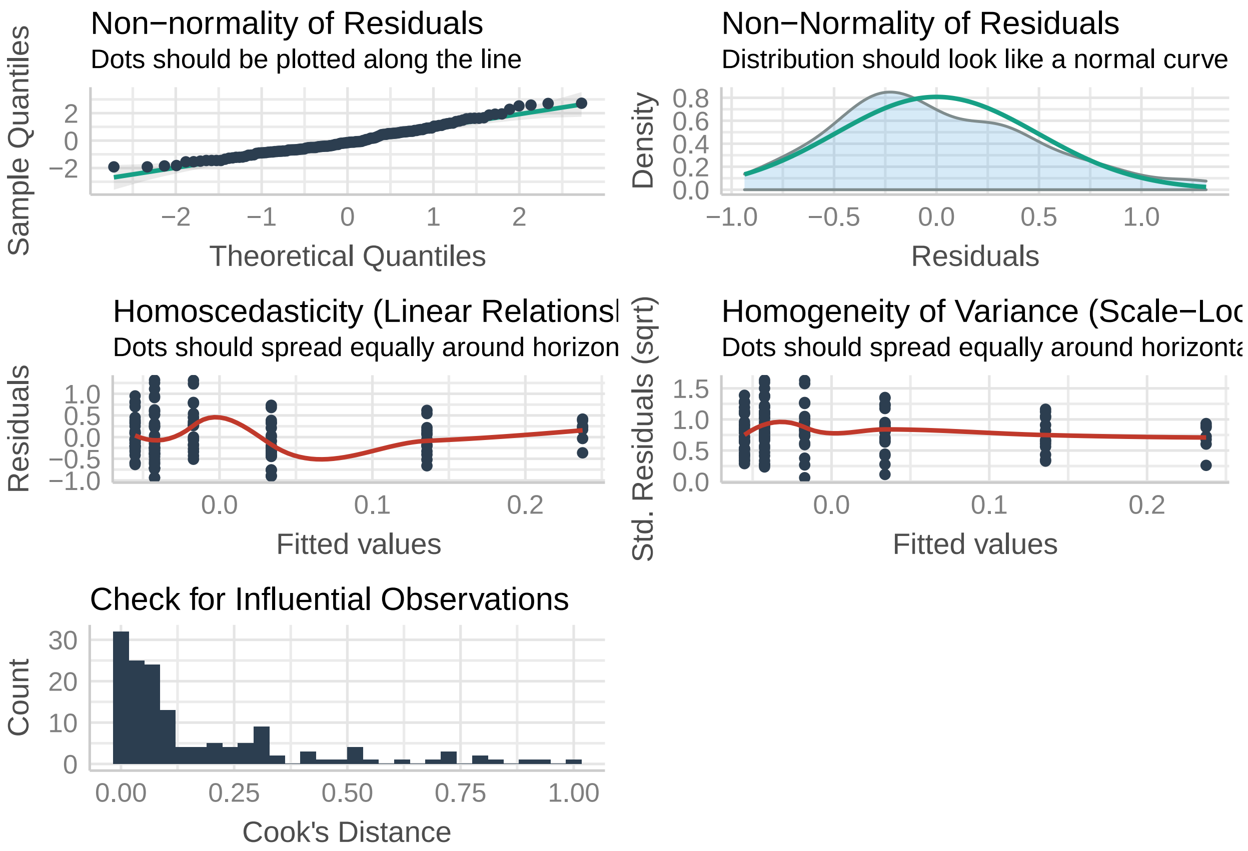

Explanatory	Estimate	SE	t.value	p.value
(Intercept)	-0.068	0.055	-1.24	0.216
Tree species richness	0.013	0.007	1.86	0.064
