# Supplementary material for: Tree diversity and soil chemical properties drive the linkages between soil microbial community and ecosystem functioning
Source: ISME Commun. 2021 Aug 23;1:41. doi: 10.1038/s43705-021-00040-0 (PMC9723754; doi:10.1038/s43705-021-00040-0)
Supplement: Supplementary file 8 — supplemental-data S8 [file 43705_2021_40_MOESM8_ESM.pdf]

Supplementary material S8

Statistical analyses and R outputs related to the main figure Fig. 3

Correlation matrix between the microbial facets

Pearson correlation coefficients

	Total biomass	Active biomass	B:F	Bacteria diversity	Fungi diversity	Cata	FG evenness
Total biomass	1.000	0.455	-0.290	-0.016	0.029	0.132	0.102
Active biomass	0.455	1.000	-0.167	-0.055	0.201	0.019	0.062
B:F	-0.290	-0.167	1.000	0.059	0.179	-0.133	0.070
Bacteria diversity	-0.016	-0.055	0.059	1.000	-0.014	-0.083	-0.093
Fungi diversity	0.029	0.201	0.179	-0.014	1.000	0.100	0.150
Cata	0.132	0.019	-0.133	-0.083	0.100	1.000	0.569
FG evenness	0.102	0.062	0.070	-0.093	0.150	0.569	1.000

Pearson correlation p-value

	Total biomass	Active biomass	B:F	Bacteria diversity	Fungi diversity	Cata	FG evenness
Total biomass	0.00e+00	6.46e-09	3.48e-04	8.49e-01	7.27e-01	1.09e-01	2.18e-01
Active biomass	6.46e-09	0.00e+00	4.24e-02	5.07e-01	1.45e-02	8.22e-01	4.56e-01
B:F	3.48e-04	4.24e-02	0.00e+00	4.79e-01	2.92e-02	1.07e-01	3.96e-01
Bacteria diversity	8.49e-01	5.07e-01	4.79e-01	0.00e+00	8.71e-01	3.19e-01	2.61e-01
Fungi diversity	7.27e-01	1.45e-02	2.92e-02	8.71e-01	0.00e+00	2.27e-01	6.97e-02
Cata	1.09e-01	8.22e-01	1.07e-01	3.19e-01	2.27e-01	0.00e+00	4.42e-14
FG evenness	2.18e-01	4.56e-01	3.96e-01	2.61e-01	6.97e-02	4.42e-14	0.00e+00

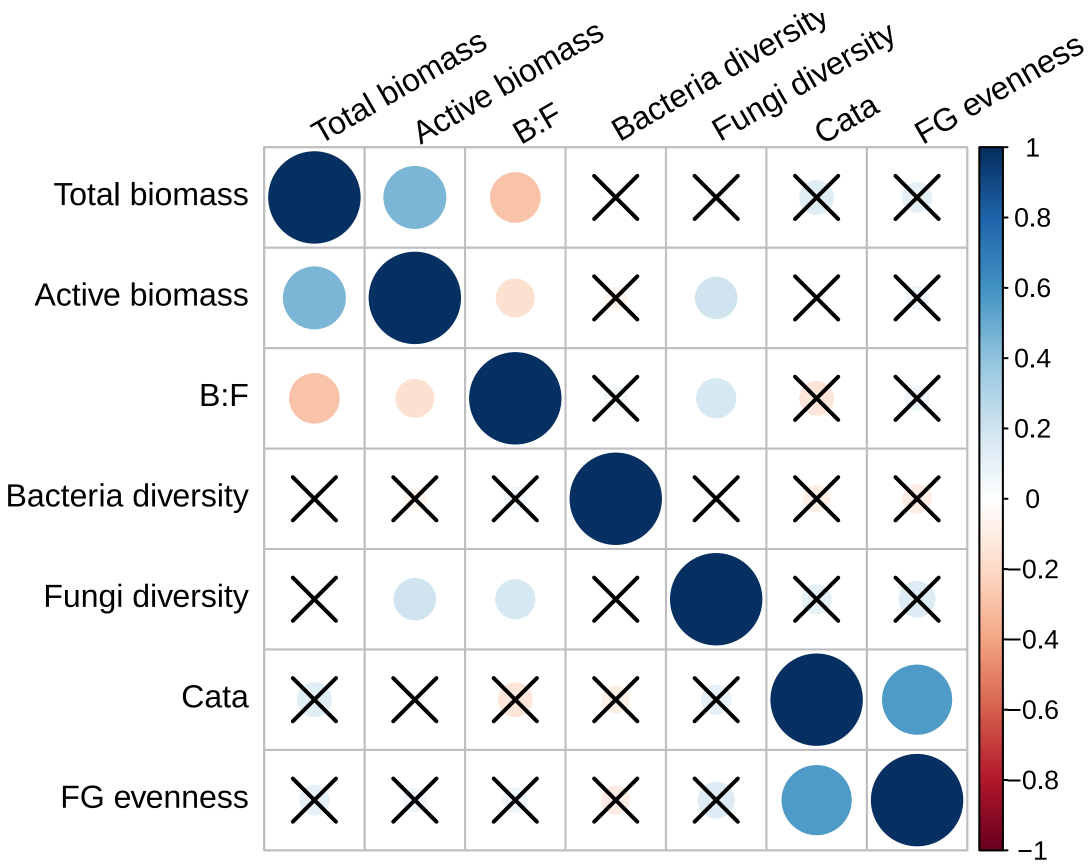

Correlation matrix between the microbial facets, non-significant correlation (Pearson correlation p-value > 0.05) were crossed.

Effect of soil microbial facets on microbial function

Microbial physiological potential

Substrate-induces respiration efficiency

Model statistical assumptions .

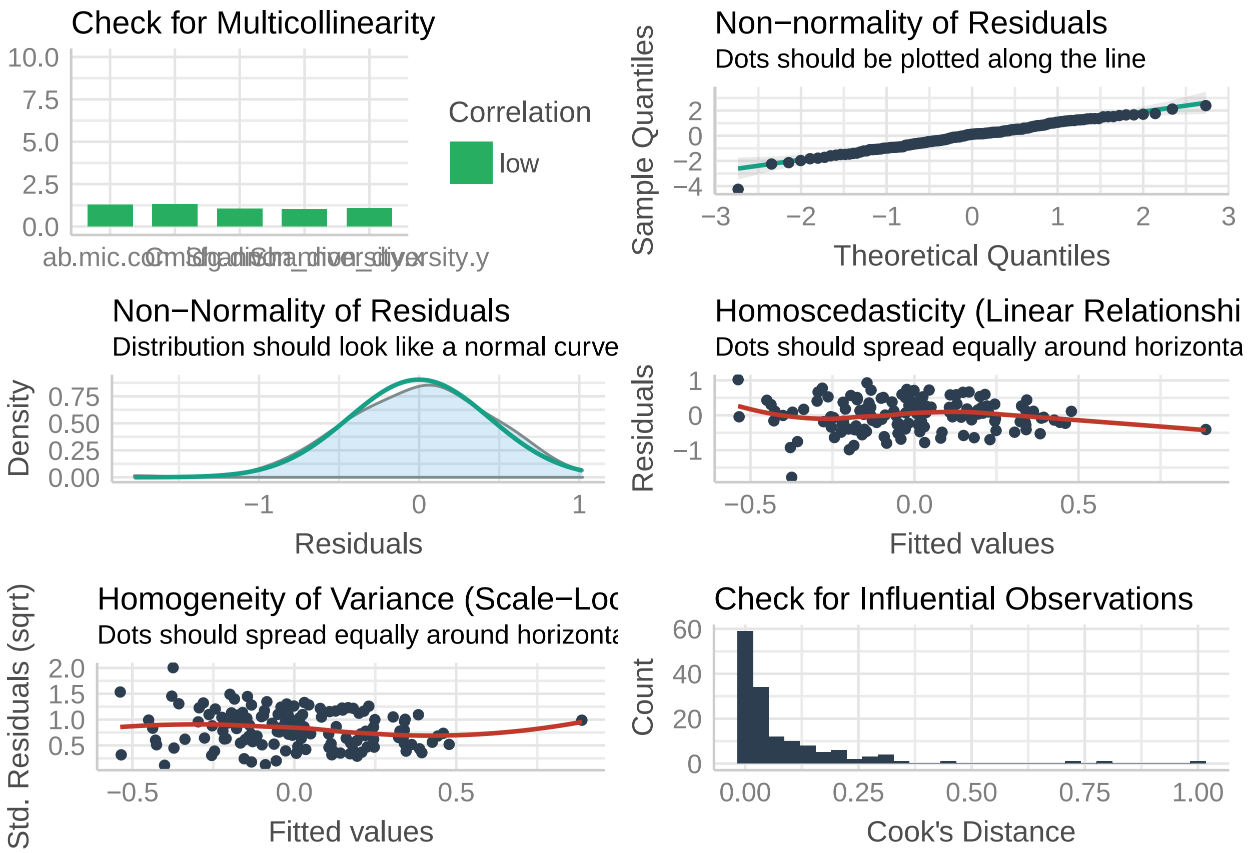

.

Model fit .

Explanatory	Estimate	SE	t.value	p.value
(Intercept)	0	0.037	0	1
Total biomass	0.238	0.084	2.84	0.005
Active biomass	0.264	0.085	3.09	0.002
Bacteria diversity	0.108	0.074	1.46	0.147
Fungi diversity	-0.152	0.077	-1.98	0.05
FG evenness	-0.166	0.076	-2.2	0.029

Variance partitioning .

Explanatory	Df	R squared	Ajusted R squared
Microbial biomass	2	0.150	0.138
Taxonomic profile	2	0.025	0.012
Functional profile	1	0.025	0.018
Microbial biomass + Taxonomic profile	4	0.195	0.172
Microbial biomass + Functional profile	3	0.189	0.172
Taxonomic profile + Functional profile	3	0.043	0.023
All	5	0.221	0.194

.

Substrate-induced respiration response range

Model statistical assumptions .

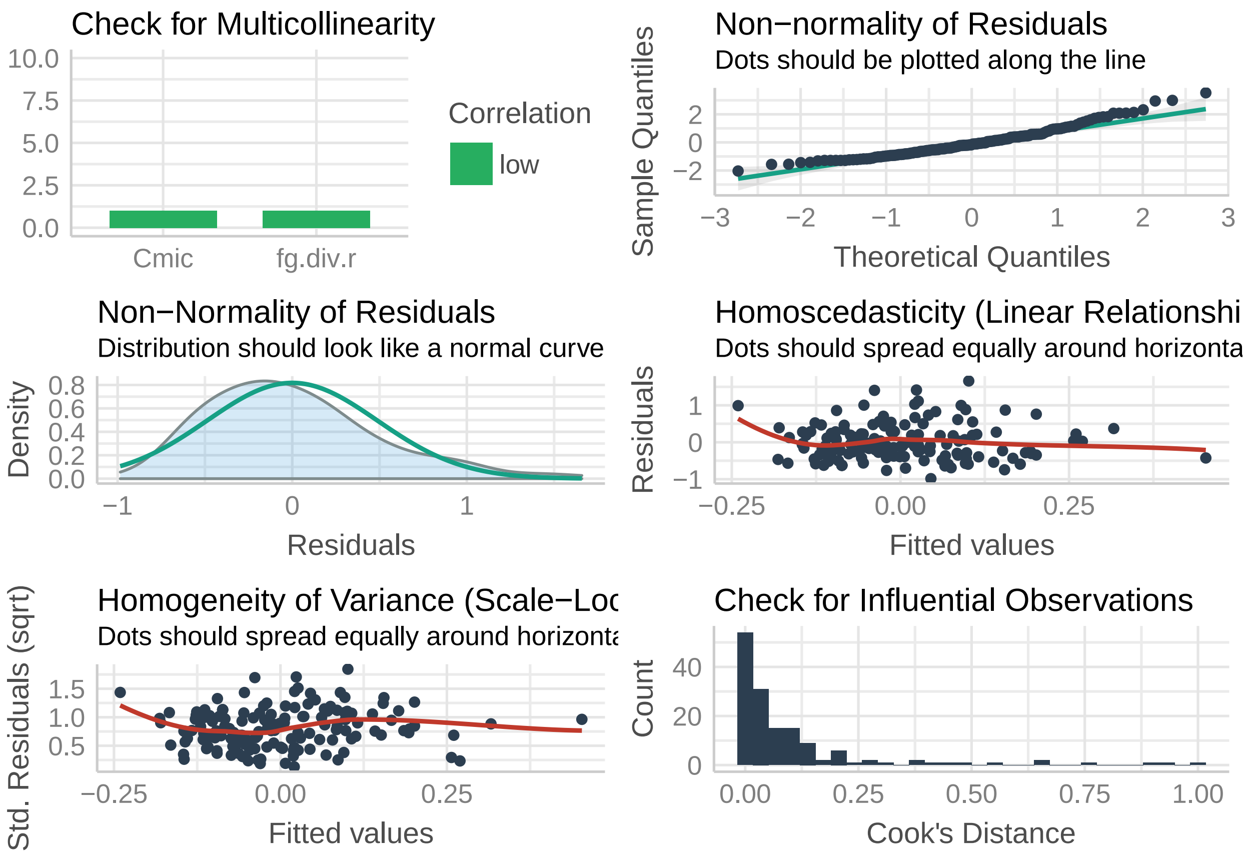

.

Model fit .

Explanatory	Estimate	SE	t.value	p.value
(Intercept)	0	0.04	0	1
Active biomass	0.175	0.081	2.16	0.033
FG evenness	-0.143	0.081	-1.77	0.079

.

Variance partitioning .

Explanatory	Df	R squared	Ajusted R squared
Microbial biomass	1	0.028	0.021
Functional profile	1	0.018	0.011
All	2	0.048	0.035

Microbial respiration

Model statistical assumptions .

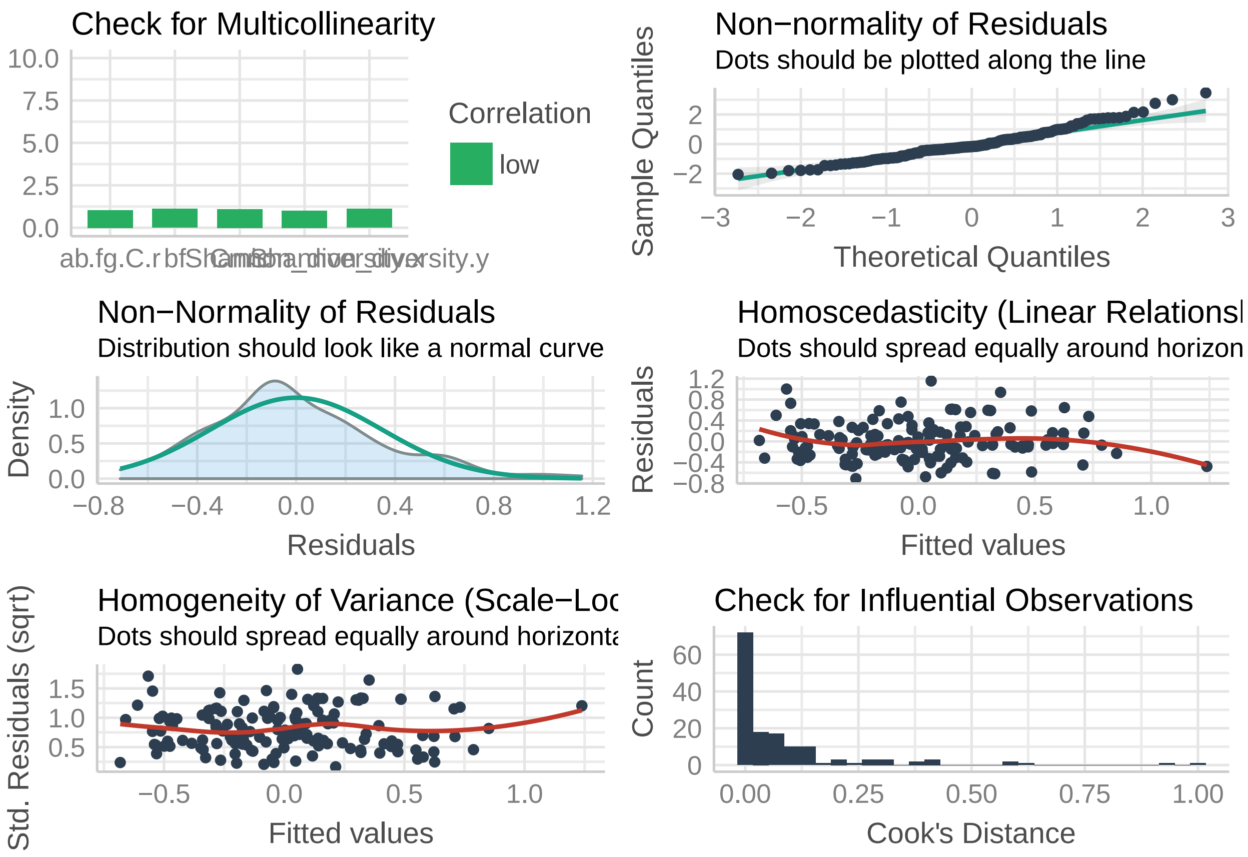

.

Model fit .

Explanatory	Estimate	SE	t.value	p.value
(Intercept)	0	0.029	0	1
Active biomass	0.675	0.061	11.07	0
B:F	-0.146	0.061	-2.37	0.019
Bacteria diversity	0.092	0.059	1.57	0.12
Fungi diversity	-0.175	0.062	-2.85	0.005
Cata	-0.132	0.059	-2.21	0.029

.

Variance partitioning .

Explanatory	Df	R squared	Ajusted R squared
Microbial biomass	1	0.431	0.427
Taxonomic profile	3	0.078	0.059
Functional profile	1	0.016	0.009
Microbial biomass + Taxonomic profile	4	0.501	0.487
Microbial biomass + Functional profile	2	0.450	0.442
Taxonomic profile + Functional profile	4	0.101	0.076
All	5	0.518	0.501

.

VIF analysis

```
Active microbial biomass                    B:F       Bacteria diversity
                    1.09                   1.11                     1.01
         Fungi diversity                   Cata
                    1.11                   1.04
```
