# Supplementary material for: Tree diversity and soil chemical properties drive the linkages between soil microbial community and ecosystem functioning
Source: ISME Commun. 2021 Aug 23;1:41. doi: 10.1038/s43705-021-00040-0 (PMC9723754; doi:10.1038/s43705-021-00040-0)
Supplement: Supplementary file 9 — supplemental-data S9 [file 43705_2021_40_MOESM9_ESM.docx]

# **Supplementary material S9 – SEMs hypotheses and rationales**

## **Expected causal relationships**

| Response variable | Explanatory variable | Hypothesis [Reference from the main text] |
| --- | --- | --- |
| Basal respiration | Total microbial biomass | Increasing soil microbial biomass should increase basal respiration [19] |
| Basal respiration | Active microbial biomass | Increasing active soil microbial biomass should increase basal respiration [19] |
| Basal respiration | B:F | Increasing B:F is expected to increase microbial community activity and thereafter, respiration [7-10] |
| Basal respiration | Bacteria diversity | Bacteria diversity should increase microbial respiration by increasing resource use [7-10] |
| Basal respiration | Fungi diversity | Fungi diversity should increase microbial respiration by increasing resource use [7-10] |
| Basal respiration | Cata | Increasing catabolism functional genes abundance (i.e. Cata) should increase microbial respiration by increasing the genetic material supporting the catabolism processes [30, 36] |
| Basal respiration | FG evenness | Increasing catabolism functional gene evenness should increase microbial respiration by increasing the physiological pathways supported by the genetic material [30, 36] |
| Basal respiration | SIR efficiency | Increasing microbial SIR efficiency should increase microbial respiration due to a higher number of physiological pathways supported [40 - 41] |
| Basal respiration | SIR range | Increasing microbial SIR range should increase microbial respiration due to a stronger response of the microbial community to complex substrates with longer pathways [40 - 41] |
| Basal respiration | TOC | Soil chemical properties affect soil functions by changing resource limitations and physiological processes [12, 13, 25, 26, 46] |
| Basal respiration | C:N | Soil chemical properties affect soil functions by changing resource limitations and physiological processes [12, 13, 25, 26, 46] |
| Basal respiration | C:P | Soil chemical properties affect soil functions by changing resource limitations and physiological processes [12, 13, 25, 26, 46] |
| Basal respiration | pH | Soil chemical properties affect soil functions by changing resource limitations and physiological processes [12, 13, 25, 26, 46] |
| Basal respiration | RH | Soil chemical properties affect soil functions by changing resource limitations and physiological processes [12, 13, 25, 26, 46] |
| Basal respiration | Tree species richness | Increasing tree species richness should increase microbial respiration by providing a higher amount and diversity of substrates [11, 21-22, 24] |
| SIR efficiency | Biomass | Increasing microbial biomass should increase SIR efficiency by reducing microbial lag time before the exponential growth [19, 45] |
| SIR efficiency | Active microbial biomass | Increasing microbial biomass should increase SIR efficiency by reducing microbial lag time before the exponential growth [19, 45] |
| SIR efficiency | B:F | Changes in microbial community composition are expected to affect microbial processes [42 - 44] |
| SIR efficiency | Bacteria diversity | Changes in microbial community composition are expected to affect microbial processes [42 - 44] |
| SIR efficiency | Fungi diversity | Changes in microbial community composition are expected to affect microbial processes [42 - 44] |
| SIR efficiency | Cata | Increasing catabolism functional genes should increase SIR efficiency by reducing microbial lag time before the exponential growth [37, 39] |
| SIR efficiency | FG evenness | Increasing catabolism functional gene evenness should increase SIR efficiency by optimizing all physiological pathways [37, 39] |
| SIR efficiency | TOC | Soil chemical properties affect soil functions by changing resource limitations and physiological processes [12, 13, 25, 26, 27] |
| SIR efficiency | C:N | Soil chemical properties affect soil functions by changing resource limitations and physiological processes [12, 13, 25, 26] |
| SIR efficiency | C:P | Soil chemical properties affect soil functions by changing resource limitations and physiological processes [12, 13, 25, 26] |
| SIR efficiency | pH | Soil chemical properties affect soil functions by changing resource limitations and physiological processes [12, 13, 25, 26] |
| SIR efficiency | RH | Soil chemical properties affect soil functions by changing resource limitations and physiological processes [12, 13, 25, 26] |
| SIR efficiency | Tree species richness | Increasing tree species richness should increase microbial physiological potential by providing a higher amount and diversity of substrates [11, 21-22, 24] |
| SIR range | Biomass | Increasing microbial biomass should increase SIR range by reducing microbial lag time before the exponential growth and favor long physiological pathways [19, 45] |
| SIR range | Active microbial biomass | Increasing microbial biomass should increase SIR efficiency by reducing microbial lag time before the exponential growth [19, 45] |
| SIR range | B:F | Changes in microbial community composition are expected to affect microbial processes [42 - 44] |
| SIR range | Bacteria diversity | Changes in microbial community composition are expected to affect microbial processes [42 - 44] |
| SIR range | Fungi diversity | Changes in microbial community composition are expected to affect microbial processes [42 - 44] |
| SIR range | Cata | Increasing catabolism functional genes should increase SIR range by reducing microbial lag before the exponential growth and favor long physiological pathways [37, 39] |
| SIR range | FG evenness | Increasing catabolism functional gene evenness should increase SIR range by optimizing all physiological pathways [37, 39] |
| SIR range | TOC | Soil chemical properties affect soil microbial functions (such as microbial growth) by changing resource limitations and physiological processes [13] |
| SIR range | C:N | Soil chemical properties affect soil microbial functions (such as microbial growth) by changing resource limitations and physiological processes [13] |
| SIR range | C:P | Soil chemical properties affect soil microbial functions (such as microbial growth) by changing resource limitations and physiological processes [13] |
| SIR range | pH | Soil chemical properties affect soil microbial functions (such as microbial growth) by changing resource limitations and physiological processes [13] |
| SIR range | RH | Soil chemical properties affect soil microbial functions (such as microbial growth) by changing resource limitations and physiological processes [13] |
| SIR range | Tree species richness | Increasing tree species richness should increase microbial physiological potential by providing a higher amount and diversity of substrates [11, 21-22, 24] |
| Biomass | TOC | Soil chemical properties affect soil microbial functions (such as microbial growth) by affecting resource limitations and physiological processes [13] |
| Biomass | C:N | Soil chemical properties affect soil microbial functions (such as microbial growth) by affecting resource limitations and physiological processes [13] |
| Biomass | C:P | Soil chemical properties affect soil microbial functions (such as microbial growth) by affecting resource limitations and physiological processes [13] |
| Biomass | pH | Soil chemical properties affect soil microbial functions (such as microbial growth) by affecting resource limitations and physiological processes [13] |
| Biomass | RH | Soil chemical properties affect soil microbial functions (such as microbial growth) by affecting resource limitations and physiological processes [13] |
| Biomass | Tree species richness | Increase of tree species richness should increase substrate abundance and therefore the system’s carrying capacity [16, 21-22, 24] |
| Active microbial biomass | TOC | Soil chemical properties affect soil microbial functions (such as microbial growth) by changing resource limitations and physiological processes [13] |
| Active microbial biomass | C:N | Soil chemical properties affect soil microbial functions (such as microbial growth) by changing resource limitations and physiological processes [13] |
| Active microbial biomass | C:P | Soil chemical properties affect soil microbial functions (such as microbial growth) by changing resource limitations and physiological processes [13] |
| Active microbial biomass | pH | Soil chemical properties affect soil microbial functions (such as microbial growth) by changing resource limitations and physiological processes [13] |
| Active microbial biomass | RH | Soil chemical properties affect soil microbial functions (such as microbial growth) by changing resource limitations and physiological processes [13] |
| Active microbial biomass | Tree species richness | Increase of tree species richness increases substrate abundance and therefore the system’s carrying capacity [21-22] |
| B:F | TOC | Soil chemical properties shape microbial community structure [13, 16] |
| B:F | C:N | Soil chemical properties shape microbial community structure [13, 16] |
| B:F | C:P | Soil chemical properties shape microbial community structure [13, 16] |
| B:F | pH | Soil chemical properties shape microbial community structure [13, 16] |
| B:F | RH | Soil chemical properties shape microbial community structure [13, 16] |
| B:F | Tree species richness | Tree species richness should increase bacteria to fungi ratio [21] |
| Bacteria diversity | TOC | Soil chemical properties shape microbial community structure [13, 16] |
| Bacteria diversity | C:N | Soil chemical properties shape microbial community structure [13, 16] |
| Bacteria diversity | C:P | Soil chemical properties shape microbial community structure [13, 16] |
| Bacteria diversity | pH | Soil chemical properties shape microbial community structure [13, 16] |
| Bacteria diversity | RH | Soil chemical properties shape microbial community structure [13, 16] |
| Bacteria diversity | Tree species richness | Increase of tree species richness increases substrate diversity and therefore functional niche complementarity [21-22] |
| Fungi diversity | TOC | Soil chemical properties shape microbial community structure [13, 16] |
| Fungi diversity | C:N | Soil chemical properties shape microbial community structure [13, 16] |
| Fungi diversity | C:P | Soil chemical properties shape microbial community structure [13, 16] |
| Fungi diversity | pH | Soil chemical properties shape microbial community structure [13, 16] |
| Fungi diversity | RH | Soil chemical properties shape microbial community structure [13, 16] |
| Fungi diversity | Tree species richness | Increase of tree species richness increases substrate diversity and therefore functional niche complementarity [21-22] |
| Cata | TOC | Soil chemical properties shape microbial community structure [12, 13, 25, 26, 30-32] |
| Cata | C:N | Soil chemical properties shape microbial community structure [12, 13, 25, 26, 30-32] |
| Cata | C:P | Soil chemical properties shape microbial community structure [12, 13, 25, 26, 30-32] |
| Cata | pH | Soil chemical properties shape microbial community structure [12, 13, 25, 26, 30-32] |
| Cata | RH | Soil chemical properties shape microbial community structure [12, 13, 25, 26, 30-32] |
| Cata | Tree species richness | Increase of tree species richness increases substrate diversity and therefore functional niche complementarity [21-22] |
| FG evenness | TOC | Soil chemical properties affect soil microbial community composition by changing resource limitations and therefore species selection [12, 13, 25, 26, 30-32] |
| FG evenness | C:N | Soil chemical properties affect soil microbial community composition by changing resource limitations and therefore species selection [12, 13, 25, 26, 30-32] |
| FG evenness | C:P | Soil chemical properties affect soil microbial community composition by changing resource limitations and therefore species selection [12, 13, 25, 26, 30-32] |
| FG evenness | pH | Soil chemical properties affect soil microbial community composition by changing resource limitations and therefore species selection [12, 13, 25, 26, 30-32] |
| FG evenness | RH | Soil chemical properties affect soil microbial community composition by changing resource limitations and therefore species selection [12, 13, 25, 26, 30-32] |
| FG evenness | Tree species richness | Increasing tree species richness increases substrate diversity and therefore functional niche complementarity [21-22] |

## **Correlations (relationships where directionality of effects is not clear from the literature)**

| First variable | Second variable | Hypothesis [Reference from the main text] |
| --- | --- | --- |
| Biomass | Active microbial biomass | We expect the biomass of active microbes to increase with increasing total microbial biomass |
| Biomass | B:F | We expect the B:F ratio to positively correlate with the microbial biomass |
| Biomass | Bacteria diversity | We expect a positive biomass ~ diversity relationship |
| Biomass | Fungi diversity | We expect a positive biomass ~ diversity relationship |
| Biomass | Cata | The number of genes copies is expected to increase with the number of cells |
| Biomass | FG evenness | We expect a positive biomass ~ diversity relationship |
| Active microbial biomass | B:F | We expect the B:F ratio to positively correlate with the microbial biomass |
| Active microbial biomass | Bacteria diversity | We expect a positive biomass ~ diversity relationship |
| Active microbial biomass | Fungi diversity | We expect a positive biomass ~ diversity relationship |
| Active microbial biomass | Cata | The number of genes copies is expected to increase with the number of cells |
| Active microbial biomass | FG evenness | We expect a positive biomass ~ diversity relationship |
| B:F | Bacteria diversity | We expect a positive biomass ~ diversity relationship, which also implies a positive B:F ~ bacteria diversity relationship |
| B:F | Fungi diversity | We expect a positive biomass ~ diversity relationship, which also implies a positive B:F ~ bacteria diversity relationship |
| B:F | Cata | We expect a positive relationship, as most of the measured genes are bacterial |
| B:F | FG evenness | We expect a positive relationship, as most of the measured genes are bacterial |
| Bacteria diversity | Fungi diversity | We expect bacteria and fungi diversity to be positively correlated to each another as driven by similar processes |
| Bacteria diversity | Cata | We expect a positive biomass ~ diversity relationship [33] |
| Bacteria diversity | FG evenness | We expect taxonomic and functional diversity to be strongly positively correlated to each another as driven by similar processes [33] |
| Fungi diversity | Cata | We expect a positive biomass ~ diversity relationship [33] |
| Fungi diversity | FG evenness | We expect taxonomic and functional diversity to be strongly correlated to each another as driven by similar processes [33] |
| Cata | FG evenness | We expect a positive biomass ~ diversity relationship |
| SIR efficiency | SIR range | We expect SIR range and efficiency to be positively correlated |
| TOC | C:N | We expect soil chemical properties to be correlated [see Scholten et al. 2017] |
| TOC | C:P | We expect soil chemical properties to be correlated [see Scholten et al. 2017] |
| TOC | pH | We expect soil chemical properties to be correlated [see Scholten et al. 2017] |
| TOC | RH | We expect soil chemical properties to be correlated [see Scholten et al. 2017] |
| TOC | Tree species richness | We expect soil chemical properties and tree species richness may be correlated; while significant tree diversity effects on soil properties can be expected, initial plot selection could also have caused non-causal relationships |
| C:N | C:P | We expect soil chemical properties to be correlated [see Scholten et al. 2017] |
| C:N | pH | We expect soil chemical properties to be correlated [see Scholten et al. 2017] |
| C:N | RH | We expect soil chemical properties to be correlated [see Scholten et al. 2017] |
| C:N | Tree species richness | We expect soil chemical properties and tree species richness may be correlated; while significant tree diversity effects on soil properties can be expected, initial plot selection could also have caused non-causal relationships |
| C:P | pH | We expect soil chemical properties to be correlated [see Scholten et al. 2017] |
| C:P | RH | We expect soil chemical properties to be correlated [see Scholten et al. 2017] |
| C:P | Tree species richness | We expect soil chemical properties and tree species richness may be correlated; while significant tree diversity effects on soil properties can be expected, initial plot selection could also have caused non-causal relationships |
| pH | RH | We expect soil chemical properties to be correlated [see Scholten et al. 2017] |
| pH | Tree species richness | We expect soil chemical properties and tree species richness may be correlated; while significant tree diversity effects on soil properties can be expected, initial plot selection could also have caused non-causal relationships |
| RH | Tree species richness | We expect soil chemical properties and tree species richness may be correlated; while significant tree diversity effects on soil properties can be expected, initial plot selection could also have caused non-causal relationships |

References

**REFERENCES ARE FROM THE MAIN TEXT**

[Scholten et al 2017] Scholten T, *et al.* On the combined effect of soil fertility and topography on tree growth in subtropical forest ecosystems—a study from SE China. Journal of Plant Ecology 2017; 10(1): 111–27
[https://doi.org/10.1093/jpe/rtw065]
