# Supplementary material for: Tree diversity and soil chemical properties drive the linkages between soil microbial community and ecosystem functioning
Source: ISME Commun. 2021 Aug 23;1:41. doi: 10.1038/s43705-021-00040-0 (PMC9723754; doi:10.1038/s43705-021-00040-0)
Supplement: Supplementary file 10 — supplemental-data S10 [file 43705_2021_40_MOESM10_ESM.pdf]

Supplementary material S10

Contents

1 Introduction **1**

2 Model structure **2**

3 Model fit **3**

3.1 Fit quality 3

3.2 Explained variance 3

3.3 Model output 4

4 Model inflation by measurement methods **5**

4.1 Fit quality 5

4.2 Explained variance 5

4.3 Model output 6

1 Introduction

The following document will display the R summary after fitting the structural equation model displayed in figure Fig. 4. The model was fitted using the “lavaan” package. (See all hypotheses rational and references in S9)

2 Model structure

```
form =
'
# Causal relations

Basal respiration ~ Biomass + Active biomass +
                      B:F + Bacteria diversity + Fungi diversity +
                      Cata + FG evenness +
                      SIR efficiency + SIR range

SIR efficiency ~ Biomass + Active biomass +
                      B:F + Bacteria diversity + Fungi diversity +
                      Cata + FG evenness

SIR range ~ Biomass + Active biomass +
                      B:F + Bacteria diversity + Fungi diversity +
                      Cata + FG evenness

# Correlations

Biomass ~~ Active biomass
Biomass ~~ B:F
Biomass ~~ Bacteria diversity
Biomass ~~ Fungi diversity
Biomass ~~ Cata
Biomass ~~ FG evenness

Active biomass ~~ B:F
Active biomass ~~ Bacteria diversity
Active biomass ~~ Fungi diversity
Active biomass ~~ Cata
Active biomass ~~ FG evenness

B:F ~~ Bacteria diversity
B:F ~~ Fungi diversity
B:F ~~ Cata
B:F ~~ FG evenness

Bacteria diversity ~~ Fungi diversity
Bacteria diversity ~~ Cata
Bacteria diversity ~~ FG evenness

Fungi diversity ~~ Cata
Fungi diversity ~~ FG evenness

Cata ~~ FG evenness

SIR range ~~ SIR efficiency'
```

3 Model fit

3.1 Fit quality

.

Fit index	Value
cfi	1
rmsea	0
srmr	0

3.2 Explained variance

.

Variable	R.squared
Basal respiration	0.573
SIR eff.	0.232
SIR range	0.084

3.3 Model output

Response	Relation	Explanatory	Estimate	SE	p value
Basal respiration	~	Total biomass	-0.034	0.065	0.595
Basal respiration	~	Active biomass	0.590	0.060	< 0.001***
Basal respiration	~	B:F	-0.109	0.060	0.067
Basal respiration	~	Bacteria diversity	0.046	0.055	0.409
Basal respiration	~	Fungi diversity	-0.128	0.058	0.027 *
Basal respiration	~	Cata	-0.113	0.067	0.094
Basal respiration	~	FG evenness	0.020	0.068	0.771
Basal respiration	~	SIR eff.	0.176	0.062	0.005 **
Basal respiration	~	SIR range	0.213	0.057	< 0.001***
SIR eff.	~	Total biomass	0.209	0.083	0.012 *
SIR eff.	~	Active biomass	0.258	0.082	0.002 **
SIR eff.	~	B:F	-0.096	0.079	0.222
SIR eff.	~	Bacteria diversity	0.115	0.072	0.11
SIR eff.	~	Fungi diversity	-0.134	0.076	0.077
SIR eff.	~	Cata	0.037	0.090	0.685
SIR eff.	~	FG evenness	-0.179	0.089	0.045 *
SIR range	~	Total biomass	0.096	0.092	0.295
SIR range	~	Active biomass	0.148	0.091	0.103
SIR range	~	B:F	-0.047	0.086	0.586
SIR range	~	Bacteria diversity	0.111	0.079	0.16
SIR range	~	Fungi diversity	-0.099	0.083	0.233
SIR range	~	Cata	-0.039	0.099	0.694
SIR range	~	FG evenness	-0.101	0.098	0.302
Total biomass	~~	Active biomass	0.455	0.065	< 0.001***
Total biomass	~~	B:F	-0.290	0.075	< 0.001***
Total biomass	~~	Bacteria diversity	-0.016	0.082	0.847
Total biomass	~~	Fungi diversity	0.029	0.082	0.725
Total biomass	~~	Cata	0.132	0.081	0.101
Total biomass	~~	FG evenness	0.102	0.081	0.211
Active biomass	~~	B:F	-0.167	0.080	0.037 *
Active biomass	~~	Bacteria diversity	-0.055	0.082	0.503
Active biomass	~~	Fungi diversity	0.201	0.079	0.011 *
Active biomass	~~	Cata	0.019	0.082	0.82
Active biomass	~~	FG evenness	0.062	0.082	0.451
B:F	~~	Bacteria diversity	0.059	0.082	0.474
B:F	~~	Fungi diversity	0.179	0.080	0.024 *
B:F	~~	Cata	-0.133	0.081	0.1
B:F	~~	FG evenness	0.070	0.082	0.39
Bacteria diversity	~~	Fungi diversity	-0.014	0.082	0.869
Bacteria diversity	~~	Cata	-0.083	0.082	0.312
Bacteria diversity	~~	FG evenness	-0.093	0.081	0.254
Fungi diversity	~~	Cata	0.100	0.081	0.22
Fungi diversity	~~	FG evenness	0.150	0.080	0.063
Cata	~~	FG evenness	0.569	0.056	< 0.001***
SIR eff.	~~	SIR range	-0.185	0.079	0.02 *
Basal respiration	~~	Basal respiration	0.427	0.053	< 0.001***

(continued)

Response	Relation	Explanatory	Estimate	SE	p value
SIR eff.	~~	SIR eff.	0.768	0.061	< 0.001***
SIR range	~~	SIR range	0.916	0.044	< 0.001***

4 Model inflation by measurement methods

Active microbial biomass and microbial respiration were measured using the same machine and subsample. Therefore, we are testing the stability of our observation and results when removing microbial biomass.

4.1 Fit quality

.

Fit index	Value
cfi	1
rmsea	0
srmr	0

4.2 Explained variance

.

Variable	R.squared
Basal respiration	0.336
SIR eff.	0.182
SIR range	0.068

4.3 Model output

Response	Relation	Explanatory	Estimate	SE	p value
Basal respiration	~	Total biomass	0.156	0.076	0.039 *
Basal respiration	~	B:F	-0.141	0.074	0.056
Basal respiration	~	Bacteria diversity	-0.006	0.068	0.935
Basal respiration	~	Fungi diversity	0.013	0.070	0.855
Basal respiration	~	Cata	-0.170	0.083	0.041 *
Basal respiration	~	FG evenness	0.082	0.085	0.333
Basal respiration	~	SIR eff.	0.328	0.072	< 0.001***
Basal respiration	~	SIR range	0.304	0.068	< 0.001***
SIR eff.	~	Total biomass	0.320	0.075	< 0.001***
SIR eff.	~	B:F	-0.121	0.081	0.135
SIR eff.	~	Bacteria diversity	0.104	0.075	0.162
SIR eff.	~	Fungi diversity	-0.081	0.076	0.292
SIR eff.	~	Cata	0.010	0.093	0.911
SIR eff.	~	FG evenness	-0.167	0.092	0.07
SIR range	~	Total biomass	0.159	0.083	0.055
SIR range	~	B:F	-0.061	0.087	0.482
SIR range	~	Bacteria diversity	0.104	0.080	0.189
SIR range	~	Fungi diversity	-0.068	0.082	0.404
SIR range	~	Cata	-0.054	0.099	0.586
SIR range	~	FG evenness	-0.094	0.099	0.341
Total biomass	~~	B:F	-0.290	0.075	< 0.001***
Total biomass	~~	Bacteria diversity	-0.016	0.082	0.847
Total biomass	~~	Fungi diversity	0.029	0.082	0.725
Total biomass	~~	Cata	0.132	0.081	0.101
Total biomass	~~	FG evenness	0.102	0.081	0.211
B:F	~~	Bacteria diversity	0.059	0.082	0.474
B:F	~~	Fungi diversity	0.179	0.080	0.024 *
B:F	~~	Cata	-0.133	0.081	0.1
B:F	~~	FG evenness	0.070	0.082	0.39
Bacteria diversity	~~	Fungi diversity	-0.014	0.082	0.869
Bacteria diversity	~~	Cata	-0.083	0.082	0.312
Bacteria diversity	~~	FG evenness	-0.093	0.081	0.254
Fungi diversity	~~	Cata	0.100	0.081	0.22
Fungi diversity	~~	FG evenness	0.150	0.080	0.063
Cata	~~	FG evenness	0.569	0.056	< 0.001***
SIR eff.	~~	SIR range	-0.145	0.080	0.071
Basal respiration	~~	Basal respiration	0.664	0.063	< 0.001***
SIR eff.	~~	SIR eff.	0.818	0.057	< 0.001***
SIR range	~~	SIR range	0.932	0.040	< 0.001***

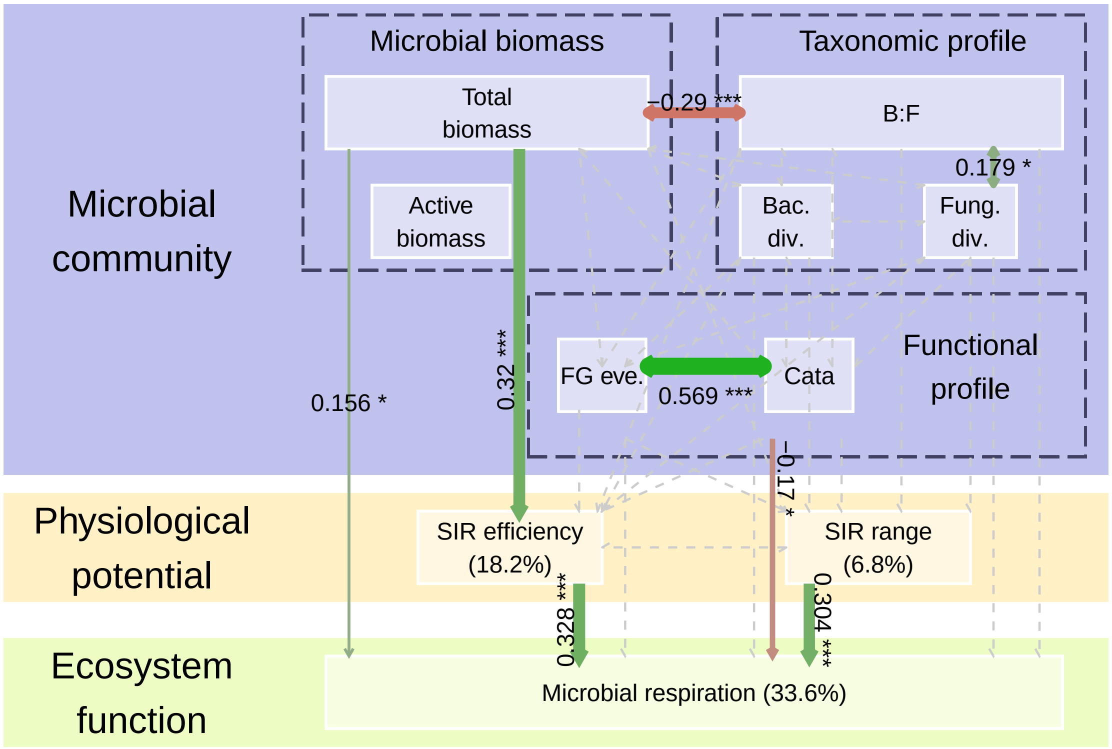

Microbial community
Physiological potential
Ecosystem function
Microbial biomass
Taxonomic profile
Total biomass
Active biomass
B:F
Bac. div.
Fung. div.
Functional profile
FG eve.
Cata
SIR efficiency (18.2%)
SIR range (6.8%)
Microbial respiration (33.6%)
−0.29 ***
0.179 *
0.569 ***
0.156 *
0.32 ***
−0.17 *
0.328 ***
0.304 ***
