# Supplementary material for: Tree diversity and soil chemical properties drive the linkages between soil microbial community and ecosystem functioning
Source: ISME Commun. 2021 Aug 23;1:41. doi: 10.1038/s43705-021-00040-0 (PMC9723754; doi:10.1038/s43705-021-00040-0)
Supplement: Supplementary file 11 — supplemental-data S11 [file 43705_2021_40_MOESM11_ESM.pdf]

Supplementary S11

Contents

1 Introduction **1**

2 Model structure **2**

3 Model fit **4**

3.1 Fit quality 4

4 Model output **4**

4.1 Explained variance 4

4.2 Summarized effects 5

4.2.1 Effect of soil and tree species richness 5

4.2.2 Link between the groups 5

4.3 Complete R summary 6

5 Model simplification **8**

5.1 Fit quality 10

5.2 Complete R summary 10

1 Introduction

The following document will display the R summary after fitting the structural equation model displayed in figure Fig. 5. The model was fitted using the “lavaan” package.(See all hypotheses rational and references in S9)

2 Model structure

```
form =
'
# Causal relations
## Ecosystem function
Basal respiration ~ Biomass + Active biomass +
                      B:F + Bacteria diversity + Fungi diversity +
                      Cata + FG evenness +
                      SIR efficiency + SIR range +
                      TOC + C:N + C:P + pH + RH + Tree.species.richness

## Physiological potentiel
SIR efficiency ~ Biomass + Active biomass +
                      B:F + Bacteria diversity + Fungi diversity +
                      Cata + FG evenness +
                      TOC + C:N + C:P + pH + RH + Tree.species.richness

SIR range ~ Biomass + Active biomass +
                      B:F + Bacteria diversity + Fungi diversity +
                      Cata + FG evenness +
                      TOC + C:N + C:P + pH + RH + Tree.species.richness

## Genetic potential
Cata ~ TOC + C:N + C:P + pH + RH + Tree.species.richness

FG evenness ~ TOC + C:N + C:P + pH + RH + Tree.species.richness

## Community structure
B:F ~ TOC + C:N + C:P + pH + RH + Tree.species.richness

Bacteria diversity ~ TOC + C:N + C:P + pH + RH + Tree.species.richness

Fungi diversity ~ TOC + C:N + C:P + pH + RH + Tree.species.richness

## Microbial biomass
Biomass ~ TOC + C:N + C:P + pH + RH + Tree.species.richness

Active biomass ~ TOC + C:N + C:P + pH + RH + Tree.species.richness

# Correlations
## Microbial community

Biomass ~~ Active biomass
Biomass ~~ B:F
Biomass ~~ Bacteria diversity
Biomass ~~ Fungi diversity
Biomass ~~ Cata
Biomass ~~ FG evenness

Active biomass ~~ B:F
Active biomass ~~ Bacteria diversity
Active biomass ~~ Fungi diversity
```

```
Active biomass ~~ Cata
Active biomass ~~ FG evenness

B:F ~~ Bacteria diversity
B:F ~~ Fungi diversity
B:F ~~ Cata
B:F ~~ FG evenness

Bacteria diversity ~~ Fungi diversity
Bacteria diversity ~~ Cata
Bacteria diversity ~~ FG evenness

Fungi diversity ~~ Cata
Fungi diversity ~~ FG evenness

Cata ~~ FG evenness

# Physiological potential
SIR range ~~ SIR efficiency

# Soil chemical properties & tree species richness
Tree.species.richness ~~ TOC
Tree.species.richness ~~ C:P
Tree.species.richness ~~ C:N
Tree.species.richness ~~ pH
Tree.species.richness ~~ RH

TOC ~~ C:P
TOC ~~ C:N
TOC ~~ pH
TOC ~~ RH

C:P ~~ C:N
C:P ~~ pH
C:P ~~ RH

C:N ~~ pH
C:N ~~ RH

pH ~~ RH'
```

3 Model fit

3.1 Fit quality

.

Fit index	Value
cfi	1
rmsea	0
srmr	0

4 Model output

4.1 Explained variance

.

Variable	R.squared
Basal respiration	0.68
SIR eff.	0.335
SIR range	0.172
Cata	0.037
FG evenness	0.045
Fungi diversity	0.055
Bacteria diversity	0.079
B:F	0.053
Active biomass	0.166
Total biomass	0.465

4.2 Summarized effects

4.2.1 Effect of soil and tree species richness

Total effects of soil chemical properties and tree species richness on microbial community facets and functions.

Variable	Total.effect
TOC	1.384
C:N	0.000
C:P	0.269
RH	0.546
pH	0.585
TreeD.	0.489

4.2.2 Link between the groups

Response	Relation	Explanatory	Total.effect
Microbial biomass	~	Soil chemical properties	1.474
Taxonomic profile	~	Soil chemical properties	0.199
Functional profile	~	Soil chemical properties	0.000
Physiological potential	~	Soil chemical properties	0.799
Microbial respiration	~	Soil chemical properties	0.312
Microbial biomass	~	Tree species richness	0.173
Taxonomic profile	~	Tree species richness	0.164
Functional profile	~	Tree species richness	0.000
Physiological potential	~	Tree species richness	0.152
Microbial respiration	~	Tree species richness	0.000
Taxonomic profile	~~	Microbial biomass	0.568
Functional profile	~~	Microbial biomass	0.000
Physiological potential	~	Microbial biomass	0.543
Microbial respiration	~	Microbial biomass	0.567
Functional profile	~~	Taxonomic profile	0.000
Physiological potential	~	Taxonomic profile	0.182
Microbial respiration	~	Taxonomic profile	0.138
Physiological potential	~	Functional profile	0.186
Microbial respiration	~	Functional profile	0.000
Microbial respiration	~	Physiological potential	0.175
Soil chemical properties	~~	Soil chemical properties	1.440
Microbial biomass	~~	Microbial biomass	0.334
Taxonomic profile	~~	Taxonomic profile	0.188
Functional profile	~~	Functional profile	0.554
Physiological potential	~~	Physiological potential	0.000

4.3 Complete R summary

Response	Relation	Explanatory	Estimate	SE	p value
Basal respiration	~	Total biomass	0.085	0.072	0.236
Basal respiration	~	Active biomass	0.567	0.057	< 0.001***
Basal respiration	~	B:F	-0.138	0.053	0.009 **
Basal respiration	~	Bacteria diversity	0.025	0.051	0.619
Basal respiration	~	Fungi diversity	-0.094	0.051	0.067
Basal respiration	~	Cata	-0.094	0.059	0.11
Basal respiration	~	FG evenness	0.011	0.060	0.859
Basal respiration	~	SIR eff.	0.090	0.058	0.12
Basal respiration	~	SIR range	0.175	0.052	< 0.001***
Basal respiration	~	TOC	-0.113	0.077	0.14
Basal respiration	~	C:P	0.096	0.064	0.134
Basal respiration	~	C:N	-0.046	0.049	0.348
Basal respiration	~	pH	-0.078	0.057	0.169
Basal respiration	~	RH	0.312	0.054	< 0.001***
Basal respiration	~	TreeD.	0.019	0.052	0.718
SIR eff.	~	Total biomass	0.093	0.101	0.356
SIR eff.	~	Active biomass	0.258	0.079	0.001 **
SIR eff.	~	B:F	-0.078	0.075	0.295
SIR eff.	~	Bacteria diversity	0.024	0.072	0.737
SIR eff.	~	Fungi diversity	-0.094	0.072	0.193
SIR eff.	~	Cata	0.044	0.084	0.603
SIR eff.	~	FG evenness	-0.186	0.085	0.028 *
SIR eff.	~	TOC	0.038	0.108	0.725
SIR eff.	~	C:P	0.113	0.089	0.204
SIR eff.	~	C:N	0.076	0.070	0.282
SIR eff.	~	pH	-0.205	0.078	0.009 **
SIR eff.	~	RH	0.042	0.075	0.578
SIR eff.	~	TreeD.	0.152	0.073	0.038 *
SIR range	~	Total biomass	0.285	0.111	0.01 *
SIR range	~	Active biomass	0.129	0.089	0.147
SIR range	~	B:F	-0.057	0.083	0.494
SIR range	~	Bacteria diversity	0.182	0.079	0.021 *
SIR range	~	Fungi diversity	-0.086	0.081	0.287
SIR range	~	Cata	-0.023	0.094	0.808
SIR range	~	FG evenness	-0.079	0.095	0.406
SIR range	~	TOC	-0.325	0.118	0.006 **
SIR range	~	C:P	0.269	0.098	0.006 **
SIR range	~	C:N	-0.051	0.078	0.518
SIR range	~	pH	0.168	0.088	0.056
SIR range	~	RH	0.151	0.083	0.069
SIR range	~	TreeD.	-0.104	0.082	0.207
Cata	~	TOC	0.089	0.103	0.391
Cata	~	C:P	-0.130	0.105	0.217
Cata	~	C:N	0.092	0.082	0.259
Cata	~	pH	-0.017	0.092	0.855
Cata	~	RH	-0.106	0.084	0.205

(continued)

Response	Relation	Explanatory	Estimate	SE	p value
Cata	~	TreeD.	0.073	0.084	0.388
FG evenness	~	TOC	0.058	0.103	0.57
FG evenness	~	C:P	-0.166	0.105	0.113
FG evenness	~	C:N	0.138	0.081	0.088
FG evenness	~	pH	-0.053	0.091	0.565
FG evenness	~	RH	-0.070	0.084	0.403
FG evenness	~	TreeD.	0.039	0.084	0.639
Fungi diversity	~	TOC	0.171	0.102	0.092
Fungi diversity	~	C:P	-0.110	0.104	0.292
Fungi diversity	~	C:N	-0.006	0.081	0.944
Fungi diversity	~	pH	0.199	0.090	0.026 *
Fungi diversity	~	RH	-0.017	0.083	0.839
Fungi diversity	~	TreeD.	0.043	0.084	0.607
Bacteria diversity	~	TOC	0.089	0.101	0.379
Bacteria diversity	~	C:P	-0.026	0.103	0.799
Bacteria diversity	~	C:N	0.100	0.080	0.212
Bacteria diversity	~	pH	-0.112	0.089	0.209
Bacteria diversity	~	RH	0.068	0.082	0.41
Bacteria diversity	~	TreeD.	0.164	0.082	0.045 *
B:F	~	TOC	-0.058	0.102	0.574
B:F	~	C:P	-0.042	0.105	0.69
B:F	~	C:N	-0.097	0.081	0.232
B:F	~	pH	0.068	0.091	0.451
B:F	~	RH	0.132	0.083	0.112
B:F	~	TreeD.	-0.108	0.083	0.194
Active biomass	~	TOC	0.407	0.092	< 0.001***
Active biomass	~	C:P	-0.033	0.098	0.74
Active biomass	~	C:N	0.037	0.076	0.631
Active biomass	~	pH	0.181	0.085	0.032 *
Active biomass	~	RH	0.067	0.078	0.391
Active biomass	~	TreeD.	0.086	0.078	0.275
Total biomass	~	TOC	0.652	0.069	< 0.001***
Total biomass	~	C:P	-0.072	0.079	0.36
Total biomass	~	C:N	0.106	0.061	0.082
Total biomass	~	pH	0.018	0.068	0.797
Total biomass	~	RH	-0.234	0.063	< 0.001***
Total biomass	~	TreeD.	0.173	0.063	0.006 **
Active biomass	~~	Total biomass	0.334	0.073	< 0.001***
B:F	~~	Total biomass	-0.244	0.077	0.002 **
Bacteria diversity	~~	Total biomass	-0.163	0.080	0.041 *
Fungi diversity	~~	Total biomass	-0.046	0.082	0.577
Cata	~~	Total biomass	0.095	0.081	0.242
FG evenness	~~	Total biomass	0.099	0.081	0.226
B:F	~~	Active biomass	-0.153	0.080	0.057
Bacteria diversity	~~	Active biomass	-0.119	0.081	0.143
Fungi diversity	~~	Active biomass	0.161	0.080	0.045 *
Cata	~~	Active biomass	0.002	0.082	0.977
FG evenness	~~	Active biomass	0.069	0.082	0.401

(continued)

Response	Relation	Explanatory	Estimate	SE	p value
Bacteria diversity	~~	B:F	0.104	0.081	0.202
Fungi diversity	~~	B:F	0.188	0.079	0.018 *
Cata	~~	B:F	-0.109	0.081	0.178
FG evenness	~~	B:F	0.097	0.081	0.235
Fungi diversity	~~	Bacteria diversity	-0.003	0.082	0.975
Cata	~~	Bacteria diversity	-0.107	0.081	0.189
FG evenness	~~	Bacteria diversity	-0.119	0.081	0.143
Cata	~~	Fungi diversity	0.084	0.082	0.305
FG evenness	~~	Fungi diversity	0.143	0.081	0.076
Cata	~~	FG evenness	0.554	0.057	< 0.001***
SIR eff.	~~	SIR range	-0.161	0.080	0.044 *
C:P	~~	TreeD.	-0.001	0.082	0.993
C:N	~~	TreeD.	0.008	0.082	0.922
pH	~~	TreeD.	-0.246	0.077	0.001 **
RH	~~	TreeD.	0.081	0.082	0.324
TOC	~~	TreeD.	0.132	0.081	0.102
TOC	~~	C:P	0.603	0.052	< 0.001***
TOC	~~	C:N	0.012	0.082	0.883
TOC	~~	pH	-0.263	0.077	< 0.001***
TOC	~~	RH	0.108	0.081	0.182
C:P	~~	C:N	-0.038	0.082	0.642
C:P	~~	pH	-0.328	0.073	< 0.001***
C:P	~~	RH	0.016	0.082	0.848
C:N	~~	pH	0.142	0.081	0.078
C:N	~~	RH	-0.123	0.081	0.129
pH	~~	RH	-0.246	0.077	0.001 **
Basal respiration	~~	Basal respiration	0.320	0.043	< 0.001***
SIR eff.	~~	SIR eff.	0.665	0.063	< 0.001***
SIR range	~~	SIR range	0.828	0.056	< 0.001***
Cata	~~	Cata	0.963	0.031	< 0.001***
FG evenness	~~	FG evenness	0.955	0.033	< 0.001***
Fungi diversity	~~	Fungi diversity	0.945	0.037	< 0.001***
Bacteria diversity	~~	Bacteria diversity	0.921	0.042	< 0.001***
B:F	~~	B:F	0.947	0.036	< 0.001***
Active biomass	~~	Active biomass	0.834	0.056	< 0.001***
Total biomass	~~	Total biomass	0.535	0.060	< 0.001***

5 Model simplification

In order to simplify our model, soil parameter have been added into a latent variable. The model fit was tested and the estimates were compared to the full model. The difference between the model output been neglectable, we favored the full model in our manuscript to leave the reader the opportunity to explore the different mechanisms. Below the simplified model and its outputs

```
form =
'
# Latent variable
```

```
fert =~ TOC + C:N + C:P + pH + RH

# Causal relations
## Ecosystem function
Basal respiration ~ Biomass + Active biomass +
                      B:F + Bacteria diversity + Fungi diversity +
                      Cata + FG evenness +
                      SIR efficiency + SIR range +
                      fert + Tree.species.richness

## Physiological potentiel
SIR efficiency ~ Biomass + Active biomass +
                      B:F + Bacteria diversity + Fungi diversity +
                      Cata + FG evenness +
                      fert + Tree.species.richness

SIR range ~ Biomass + Active biomass +
                      B:F + Bacteria diversity + Fungi diversity +
                      Cata + FG evenness +
                      fert + Tree.species.richness

## Genetic potential
Cata ~ fert + Tree.species.richness

FG evenness ~ fert + Tree.species.richness

## Community structure
B:F ~ fert + Tree.species.richness

Bacteria diversity ~ fert + Tree.species.richness

Fungi diversity ~ fert + Tree.species.richness

## Microbial biomass
Biomass ~ fert + Tree.species.richness

Active biomass ~ fert + Tree.species.richness

# Correlations
## Microbial community

Biomass ~~ Active biomass
Biomass ~~ B:F
Biomass ~~ Bacteria diversity
Biomass ~~ Fungi diversity
Biomass ~~ Cata
Biomass ~~ FG evenness

Active biomass ~~ B:F
Active biomass ~~ Bacteria diversity
Active biomass ~~ Fungi diversity
Active biomass ~~ Cata
Active biomass ~~ FG evenness
```

```
B:F ~~ Bacteria diversity
B:F ~~ Fungi diversity
B:F ~~ Cata
B:F ~~ FG evenness

Bacteria diversity ~~ Fungi diversity
Bacteria diversity ~~ Cata
Bacteria diversity ~~ FG evenness

Fungi diversity ~~ Cata
Fungi diversity ~~ FG evenness

Cata ~~ FG evenness

# Physiological potential
SIR range ~~ SIR efficiency

# Soil chemical properties & tree species richness
Tree.species.richness ~~ fert
'
```

5.1 Fit quality

.

Fit index	Value
cfi	0.82251
rmsea	0.11403
srmr	0.07261

5.2 Complete R summary

Response	Relation	Explanatory	Estimate	SE	p value
Basal respiration	~	Total biomass	-0.078	0.075	0.3
Basal respiration	~	Active biomass	0.587	0.060	< 0.001***
Basal respiration	~	B:F	-0.110	0.060	0.065
Basal respiration	~	Bacteria diversity	0.024	0.057	0.674
Basal respiration	~	Fungi diversity	-0.132	0.058	0.022 *
Basal respiration	~	Cata	-0.115	0.067	0.086
Basal respiration	~	FG evenness	0.020	0.068	0.765
Basal respiration	~	SIR eff.	0.156	0.063	0.014 *
Basal respiration	~	SIR range	0.224	0.057	< 0.001***
Basal respiration	~	fert	0.057	0.067	0.394
Basal respiration	~	TreeD.	0.068	0.058	0.245
SIR eff.	~	Total biomass	0.076	0.098	0.437
SIR eff.	~	Active biomass	0.239	0.080	0.003 **
SIR eff.	~	B:F	-0.096	0.077	0.214
SIR eff.	~	Bacteria diversity	0.052	0.073	0.479

(continued)

Response	Relation	Explanatory	Estimate	SE	p value
SIR eff.	~	Fungi diversity	-0.141	0.073	0.055
SIR eff.	~	Cata	0.027	0.087	0.757
SIR eff.	~	FG evenness	-0.171	0.087	0.05
SIR eff.	~	fert	0.162	0.088	0.065
SIR eff.	~	TreeD.	0.189	0.073	0.01 *
SIR range	~	Total biomass	0.212	0.107	0.049 *
SIR range	~	Active biomass	0.165	0.090	0.065
SIR range	~	B:F	-0.044	0.086	0.604
SIR range	~	Bacteria diversity	0.161	0.080	0.045 *
SIR range	~	Fungi diversity	-0.093	0.082	0.254
SIR range	~	Cata	-0.031	0.097	0.746
SIR range	~	FG evenness	-0.110	0.097	0.254
SIR range	~	fert	-0.153	0.097	0.112
SIR range	~	TreeD.	-0.134	0.082	0.1
Cata	~	fert	0.008	0.080	0.921
Cata	~	TreeD.	0.080	0.082	0.332
FG evenness	~	fert	-0.030	0.080	0.709
FG evenness	~	TreeD.	0.060	0.083	0.471
Fungi diversity	~	fert	0.062	0.080	0.443
Fungi diversity	~	TreeD.	0.007	0.083	0.929
Bacteria diversity	~	fert	0.102	0.078	0.193
Bacteria diversity	~	TreeD.	0.196	0.079	0.013 *
B:F	~	fert	-0.083	0.080	0.297
B:F	~	TreeD.	-0.112	0.081	0.168
Active biomass	~	fert	0.346	0.074	< 0.001***
Active biomass	~	TreeD.	0.056	0.077	0.469
Total biomass	~	fert	0.569	0.064	< 0.001***
Total biomass	~	TreeD.	0.164	0.065	0.012 *
Active biomass	~~	Total biomass	0.321	0.075	< 0.001***
B:F	~~	Total biomass	-0.274	0.075	< 0.001***
Bacteria diversity	~~	Total biomass	-0.159	0.080	0.047 *
Fungi diversity	~~	Total biomass	-0.012	0.081	0.885
Cata	~~	Total biomass	0.138	0.080	0.083
FG evenness	~~	Total biomass	0.134	0.080	0.094
B:F	~~	Active biomass	-0.137	0.080	0.089
Bacteria diversity	~~	Active biomass	-0.122	0.081	0.132
Fungi diversity	~~	Active biomass	0.191	0.079	0.016 *
Cata	~~	Active biomass	0.008	0.082	0.918
FG evenness	~~	Active biomass	0.071	0.081	0.382
Bacteria diversity	~~	B:F	0.096	0.081	0.236
Fungi diversity	~~	B:F	0.189	0.079	0.017 *
Cata	~~	B:F	-0.124	0.081	0.125
FG evenness	~~	B:F	0.076	0.082	0.354
Fungi diversity	~~	Bacteria diversity	-0.024	0.082	0.774
Cata	~~	Bacteria diversity	-0.103	0.081	0.203
FG evenness	~~	Bacteria diversity	-0.105	0.081	0.198
Cata	~~	Fungi diversity	0.099	0.081	0.225

(continued)

Response	Relation	Explanatory	Estimate	SE	p value
FG evenness	~~	Fungi diversity	0.151	0.080	0.06
Cata	~~	FG evenness	0.568	0.056	< 0.001***
SIR eff.	~~	SIR range	-0.148	0.080	0.065
fert	~~	TreeD.	0.129	0.079	0.103
TOC	~~	TOC	-0.054	0.122	0.656
C:P	~~	C:P	0.655	0.074	< 0.001***
C:N	~~	C:N	1.000	0.002	< 0.001***
pH	~~	pH	0.939	0.038	< 0.001***
RH	~~	RH	0.987	0.018	< 0.001***
Basal respiration	~~	Basal respiration	0.422	0.053	< 0.001***
SIR eff.	~~	SIR eff.	0.722	0.062	< 0.001***
SIR range	~~	SIR range	0.887	0.049	< 0.001***
Cata	~~	Cata	0.993	0.013	< 0.001***
FG evenness	~~	FG evenness	0.996	0.010	< 0.001***
Fungi diversity	~~	Fungi diversity	0.996	0.010	< 0.001***
Bacteria diversity	~~	Bacteria diversity	0.946	0.036	< 0.001***
B:F	~~	B:F	0.978	0.024	< 0.001***
Active biomass	~~	Active biomass	0.872	0.052	< 0.001***
Total biomass	~~	Total biomass	0.625	0.073	< 0.001***
fert	=~	TOC	1.027	0.059	< 0.001***
fert	=~	C:P	0.587	0.063	< 0.001***
fert	=~	C:N	0.009	0.080	0.905
fert	=~	pH	-0.246	0.077	0.001 **
fert	=~	RH	0.112	0.079	0.156
